# Oxidative Dearomative Cross-Dehydrogenative Coupling of Indoles with Diverse C-H Nucleophiles: Efficient Approach to 2,2-Disubstituted Indolin-3-ones

**DOI:** 10.3390/molecules25020419

**Published:** 2020-01-20

**Authors:** Xue Yan, Ying-De Tang, Cheng-Shi Jiang, Xigong Liu, Hua Zhang

**Affiliations:** 1 School of Chemistry and Chemical Engineering, University of Jinan, Jinan 250022, China; 2 School of Biological Science and Technology, University of Jinan, Jinan 250022, China; 3 School of Chemistry and Chemical Engineering, Shandong University, Jinan 250100, China

**Keywords:** cross coupling, dearomatization, C-H functionalization, indolin-3-ones, dimerization and trimerization of indoles

## Abstract

The oxidative, dearomative cross-dehydrogenative coupling of indoles with various C-H nucleophiles is developed. This process features a broad substrate scope with respect to both indoles and nucleophiles, affording structurally diverse 2,2-disubstituted indolin-3-ones in high yields (up to 99%). The oxidative dimerization and trimerization of indoles has also been demonstrated under the same conditions.

## 1. Introduction

Direct C-H functionalization has emerged as an elegant approach to the construction of C-C bonds [1,2,3,4,5,6,7]. Particularly, oxidative cross-dehydrogenative coupling (CDC) from two readily available C-H bonds features the advantage of high step- and atom-economy, as it does not require pre-functionalized substrates [8,9,10,11,12]. Over the past decades, oxidative CDC reactions have gained tremendous attention since the pioneering work of Li, and numerous oxidative systems have been successfully developed [13,14,15,16,17,18]. Under the developed oxidative conditions, indoles have been widely used as nucleophiles in a number of CDC reactions owing to the strong nucleophilicity of indole rings [19,20,21,22,23,24,25,26,27,28,29]. In contrast, reactions of indoles with other nucleophiles have not been well investigated [30,31,32,33,34,35]. Therefore, the development of CDC reactions from indoles with various C-H nucleophiles will provide straightforward access to structurally diverse indole derivatives and is thus highly desired.

**Figure 1:**
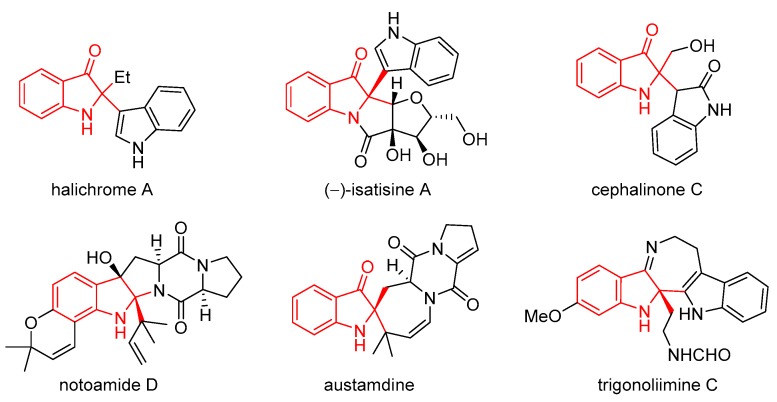
Representative bioactive natural products with 2,2-disubstituted indolin-3-one motif.

**Figure 2:**
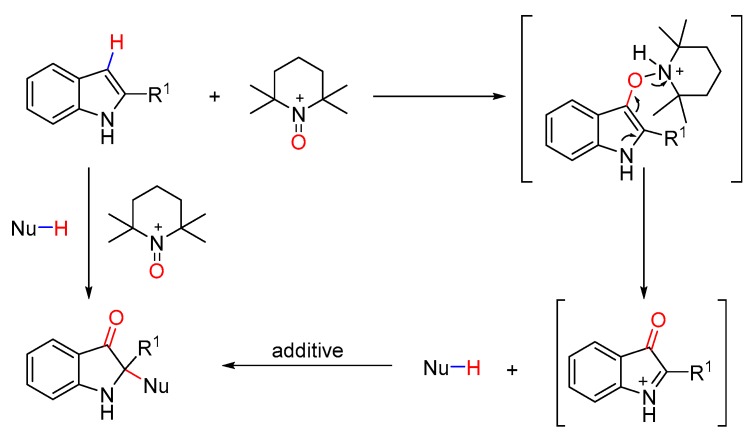
Oxidative dearomative cross-dehydrogenative coupling of indoles with various C-H nucleophiles.

As illustrated in Figure 1, 2,2-disubstituted indolin-3-ones are core scaffolds of a wide range of bioactive molecules [36,37,38,39,40,41,42], and have also been widely used as key intermediates in the total synthesis of a variety of natural products [43,44,45,46,47,48]. Therefore, great efforts have been devoted to the construction of these structures. Current syntheses are mainly based on four strategies, i.e., the oxidative rearrangement of 2,3-disubstituted indoles [49,50,51,52,53], cyclization reactions from acyclic starting materials [54,55,56,57,58,59,60,61,62], direct transformation from corresponding 3*H*-indol-3-ones or indolin-3-ones [63,64,65,66,67,68,69,70,71], and oxidative dearomatization of indoles [72,73,74,75,76]. Direct C-H functionalization of indoles with different C-H nucleophiles presents an atom-economic protocol without prior installation of activating groups and is thus very attractive. However, most of these reactions focus on the construction of di- or trimerization of indoles [50,77,78,79,80], and the reactions of indoles with dissimilar C-H nucleophiles are considerably rare [81,82,83]. Recently, we reported an efficient oxidative dearomatization reaction of indoles [84,85]. Encouraged by these results, we envisioned that oxidative dearomatization of indoles with C-H nucleophiles could be achieved under suitable conditions. Herein, we present an effective oxidative, dearomative cross-dehydrogenative coupling of indoles with a variety of C-H nucleophiles (Figure 2), affording structurally diverse 2,2-disubstituted indolin-3-ones in high yields.

## 2. Results and Discussion

The reaction of 2-phenyl-indole **1a** with diethyl malonate **2a** was initially selected to start our investigation in the presence of TEMPO^+^ClO_4_^−^ (TEMPO oxoammonium perchlorate) (Table 1). No expected product was observed when the reaction was conducted without any additive, while the dimerization product (**6a**) of **1a** was obtained in 96% yield (Table 1, entry 1). To improve the nucleophilicity of **2a**, various metal additives were applied to activate the 1,3-dicarbonyls. To our delight, the desired product **3a** was obtained in 79% yield using CuCl as additive (Table 1, entry 2). Further screening of additives revealed that this reaction proceeded more efficiently when a catalytic amount of Cu(OTf)_2_ was used, affording **3a** in 95% yield as the sole product (Table 1, entries 3–6). Next, different TEMPO oxoammonium salts were investigated (Table 1, entries 7–9), and the yield of product **3a** increased to 98% when TEMPO^+^BF_4_^−^ was used as oxidant. Notably, decreasing the amount of Cu(OTf)_2_ to 0.005 equivalent had no effect on the reactivity of the reaction (Table 1, entry 10). Moreover, under the optimized conditions, the dimer **6a** was obtained in 98% yield when no extra nucleophile was added (entry 11). Finally, the optimal conditions were established as: TEMPO^+^BF_4_^−^ (1.0 eq)/Cu(OTf)_2_ (0.005 eq)/THF.

**Figure 3:**
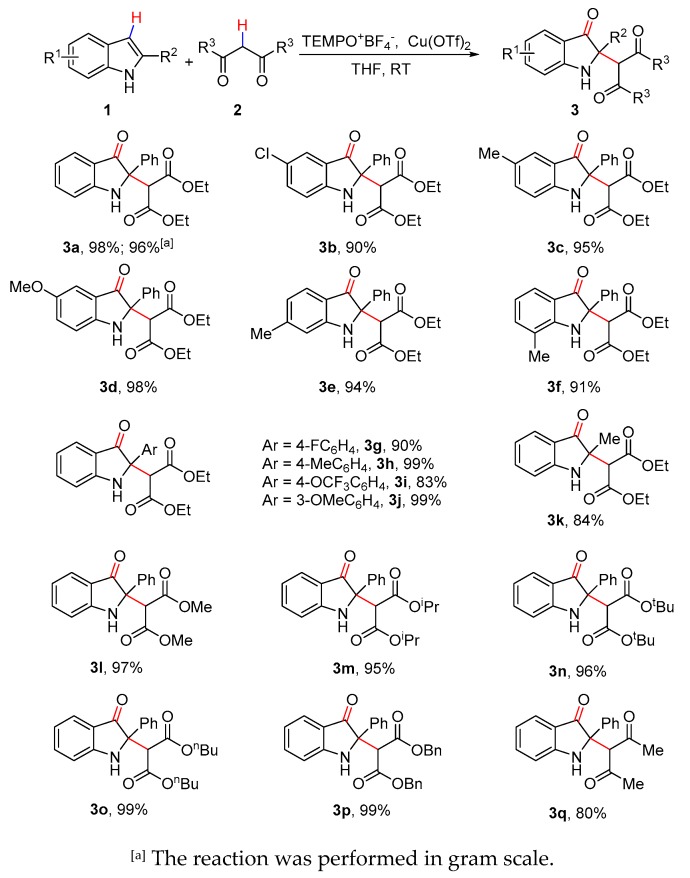
Cross-dehydrogenative coupling of indoles with 1,3-dicarbonyl compounds.

With the optimized conditions in hand, the scope with respect to both indoles (**1**) and dicarbonyl compounds (**2**) was explored (Figure 3). In general, structurally and electronically varied 2-phenyl indoles were compatible with the reaction conditions, affording the desired 2,2-disubstituted indolin-3-ones in excellent yields (**3a**–**3f**). Notably, when the reaction of **1a** and **2a** was performed in gram scale, the desired product was obtained in 96% yield. Moreover, 2-aryl indoles bearing either electron-donating or withdrawing functional groups on the aryl moiety participated in the reactions smoothly, giving indolin-3-ones **3g**–**3j** in high yields (83–99%). Electron-rich 2-aryl indoles like **1h** and **1j** afforded comparable results to that of 2-phenyl indole, while electron-deficient indoles like **1g** and **1i** gave slightly reduced yields. Excitingly, 2-methyl indole was also tolerated with the reaction conditions in good yield, which provided a straightforward approach to 2,2-dialkyl substituted indolin-3-ones. Furthermore, a variety of commercially available malonates, such as dimethyl, diisopropyl, ditert-butyl, dibutyl, and dibenzyl malonates, smoothly participated in the reaction, giving 2,2-disubstituted indolin-3-ones **3l**–**3p** in 95–99% yields. Additionally, acetylacetone was also a suitable substrate for the reaction, with only a moderately reduced yield (**3q**, 80%).

**Figure 4:**
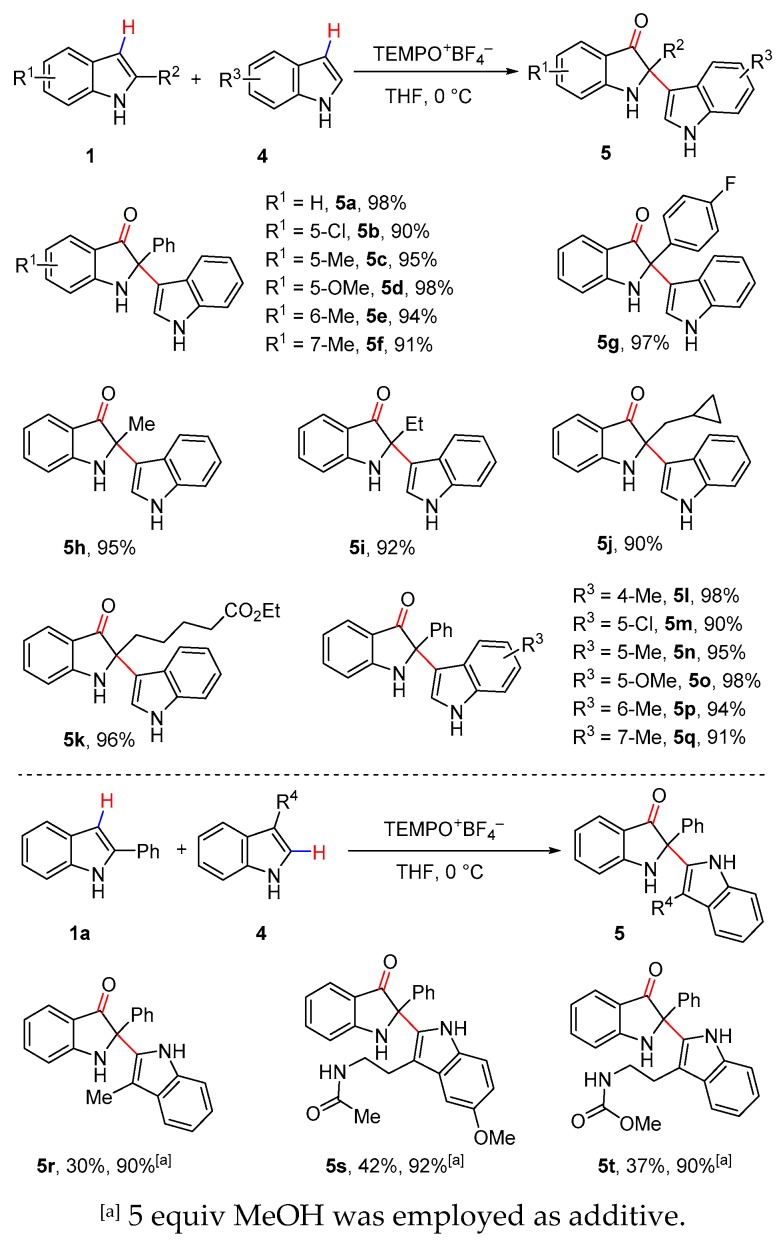
Cross-dehydrogenative coupling of indoles with dissimilar indole substrates.

Bisindole scaffolds exist in a number of bioactive natural products [42,86,87,88]. For example, isatisine A from the leaves of *Isatis indigotica* showed anti-HIV activity [89], while halichrome A from a metagenomic library derived from the marine sponge *Halichondria okadai* exhibited cytotoxicity against B16 melanoma cells [89]. Herein, the cross-dehydrogenative coupling of C-2 substituted indoles (**1**) with dissimilar indole nucleophiles (**4**) was next explored (Figure 4). When the reaction was conducted at 0 °C, a similar scope of C-2 substituted indoles as for the aforementioned dicarbonyls were tried, providing the corresponding 2,2-disusbtituted indolin-3-ones in excellent yields. The reaction of 2-phenyl indole bearing an electron-withdrawing group on indole ring gave the indolin-3-one **5b** with a slightly decreased yield. Moreover, a number of 2-alkyl indoles were also suitable for the reaction with very decent product yields (**5h**–**5k**) and displayed excellent regio-selectivity, as no benzylic oxidation products were observed. It is worth noting that natural product halichrome A (**5i**) was successfully synthesized in 92% yield using the current method. A broad range of electronically varied indoles with different substitution patterns were also found to be appropriate nucleophiles for this process, affording the expected products **5l**–**5q** in excellent yields. However, when C-3 substituted indoles such as 3-methylindole, melatonine, and tryptamine derivative were subjected to the reaction, the expected 2,2′-bisindolin-3-ones **5r**–**5t** were obtained in low yields. Excitingly, MeOH as an additive proved to be beneficial and enhanced the reactivity of the reaction, and satisfying yields (90–92%) of coupling products were achieved [89,90,91,92,93].

**Figure 5:**
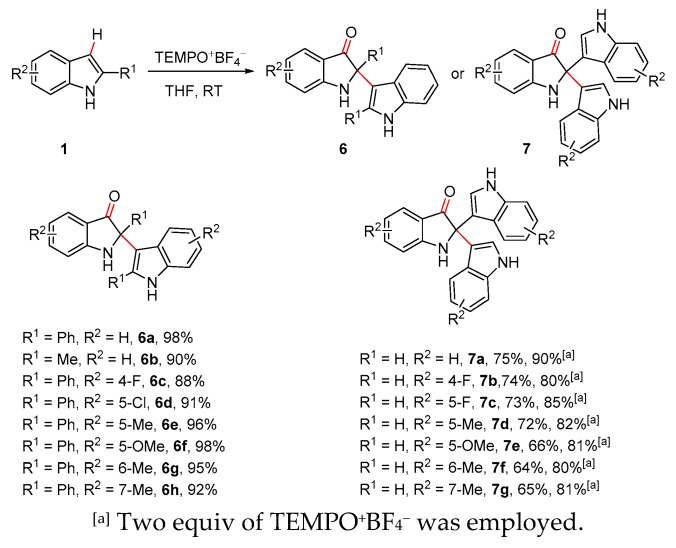
Oxidative dimerization and trimerization of indoles.

The oxidative dimerization of **1a** was realized in 96% or 98% yield without any additive and extra nucleophiles using TEMPO^+^ClO_4_^−^ or TEMPO^+^BF_4_^−^ as oxidant (Table 1, entries 1 and 11). Therefore, the scope of dimerization of C-2 substituted indoles was subsequently investigated (Figure 5). Structurally and electronically varied C-2 substituted indoles proved to be effective substrates, delivering the dimers **6a**–**6h** in excellent yields. Next, the universality of the developed method was further explored in the formation of oxidative trimers (2,2-bis(indol-3-yl)indolin-3-ones). The oxidative process exhibited excellent regio-selectivity and produced the desired trimeric products as single isomers without any 3,3-disubstituted indolin-3-ones generated, and proceeded with moderate yields. Interestingly, yields of the trimers increased remarkably to 80–90% when the reactions were conducted with excess oxidant.

**Figure 6:**
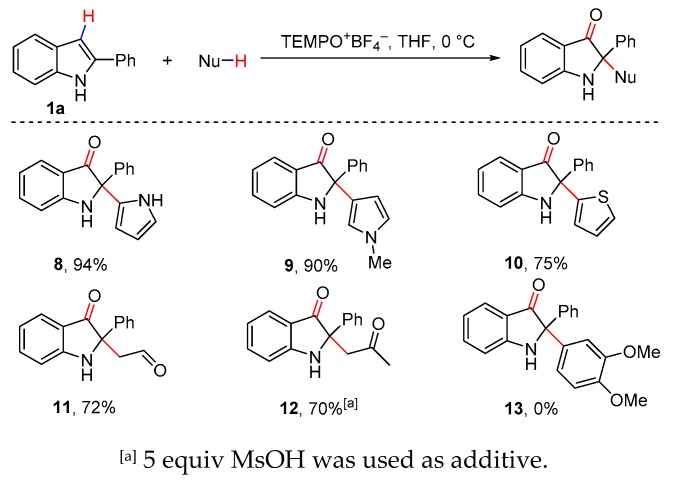
Cross-dehydrogenative coupling of indoles with various C-H nucleophiles.

The successful oxidative cross-dehydrogenative coupling of indoles with 1,3-dicarbonyl compounds and indole nucleophiles prompted us to further explore the reaction of indoles with other diverse C-H nucleophiles under the developed conditions (Figure 6). Delightedly, the CDC reactions of 2-phenyl indole **1a** with a number of C-H nucleophiles including pyrrole, thiophene, acetaldehyde and acetone, went smoothly to give the desired products **8**–**12** in good yields. It was noteworthy that C-3 position was the major reactive nucleophilic site of *N*-methyl pyrrole. However, π-rich arenes did not afford the desired products.

## 3. Materials and Methods

### 3.1. Materials

THF (Tianjin Fuyu Fine Chemical Co. Ltd., Tianjin, China) was freshly distilled over Na. Other reagents and solvents (J&K Inc. Ltd., Shanghai, China) were used as commercially available products without further purification unless specified. Proton (^1^H) and carbon (^13^C) nuclear magnetic resonance (NMR) spectra were recorded on a Bruker AVANCE DRX600 NMR spectrometer (Bruker BioSpin AG, Fällanden, Switzerland). The chemical shifts were given in parts per million (ppm) on the delta (*δ*) scale, and the residual solvent peaks were used as references as follows: CDCl_3_ *δ*_H_ 7.26, *δ*_C_ 77.16 ppm; acetone-*d*_6_ *δ*_H_ 2.05, *δ*_C_ 29.84 ppm; DMSO-*d*_6_ *δ*_H_ 2.50, *δ*_C_ 39.52 ppm. Analytical TLC was performed on precoated silica gel GF254 plates (Qingdao Haiyang Chemical Co. Ltd., Qingdao, China). Column chromatography was carried out on silica gel (200–300 mesh, Qingdao Haiyang Chemical Co. Ltd., Qingdao, China). ESIMS analyses were performed on an Agilent 1260-6460 Triple Quad LC-MS spectrometer (Agilent Technologies Inc., Waldbronn, Germany). HR-ESIMS were carried out on an Agilent 6520 Q-TOF MS spectrometer (Agilent Technologies Inc., Waldbronn, Germany).

### 3.2. General Procedure for the Oxidative Dearomative Cross-Dehydrogenative Coupling Reactions

**General procedure A:** To a solution of **1** (0.1 mmol), **2** (0.2 mmol) and Cu(OTf)_2_ (0.005 eq.) in THF (1.0 mL) was added TEMPO^+^BF_4_^−^ (0.1 mmol) at room temperature. The mixture was further stirred until the disappearance of starting indole by TLC analysis at room temperature. Then, the solvent was removed, and the residue was purified by flash chromatography using acetone-petroleum ether as eluent to afford the desired product.

**General procedure B:** To a solution of **1** (0.1 mmol) and **4** (0.2 mmol) in THF (1.0 mL) was added TEMPO^+^BF_4_^−^ (0.1 mmol) at 0 °C. The mixture was further stirred until the disappearance of starting material **1** by TLC analysis at 0 °C. The solvent was removed and the residue was purified by flash chromatography using acetone-petroleum ether as eluent to afford the desired product.

**General procedure C:** To a solution of **1** (0.1 mmol) and MeOH (0.5 mmol) in THF (1.0 mL) was added TEMPO^+^BF_4_^−^ (0.1 mmol) at 0 °C. The mixture was stirred at 0 °C until the disappearance of **1**. Nucleophiles **4r**–**4t** (0.2 mmol) were added to the mixture and the reaction was further stirred until the disappearance of intermediates by TLC analysis at 0 °C. Then, the solvent was removed and the residue was purified by flash chromatography using acetone-petroleum ether as eluent to afford the desired product.

**General procedure D:** To a solution of C2-substituted indole (0.2 mmol) or indole (0.3 mmol) in THF (1.0 mL) was added TEMPO^+^BF_4_^−^ (0.1 mmol). The mixture was stirred at room temperature for 6 h. The solvent was removed and the residue was purified by flash chromatography using acetone-petroleum ether as eluent to afford the desired product.

For original ^1^H and ^13^C NMR spectra of all synthesized compounds please see the Supplementary Materials.

***Diethyl 2-(3-oxo-2-phenylindolin-2-yl)malonate (*3a*).*** According to procedure A, **3a** was obtained as a yellow solid in 98% yield (36.0 mg; flash chromatographic condition: petroleum ether-acetone 90:10). ^1^H NMR (600 MHz, CDCl_3_) *δ* 7.56 (d, *J* = 7.7 Hz, 1H), 7.54–7.51 (m, 2H), 7.49–7.45 (m, 1H), 7.30 (t, *J* = 7.6 Hz, 2H), 7.25 (t, *J* = 7.3 Hz, 1H), 6.97 (d, *J* = 8.2 Hz, 1H), 6.81 (t, *J* = 7.4 Hz, 1H), 6.09 (s, 1H), 4.72 (s, 1H), 4.10–3.99 (m, 3H), 3.91 (dq, *J* = 10.8, 7.1 Hz, 1H), 1.02 (t, *J* = 7.1 Hz, 3H), 0.85 (t, *J* = 7.2 Hz, 3H); ^13^C NMR (151 MHz, CDCl_3_) *δ* 198.1 (C=O), 167.9 (C=O), 166.4 (C=O), 160.2 (Cq), 137.4 (CH), 136.9 (Cq), 128.9 (CH, 2C), 128.2 (CH), 125.5 (CH), 125.4 (CH, 2C), 119.6 (Cq), 119.2 (CH), 111.5 (CH), 70.4 (Cq), 62.0 (CH_2_), 61.7 (CH_2_), 58.8 (CH), 13.8 (CH_3_), 13.4 (CH_3_); HR-ESIMS *m*/*z* calcd for C_21_H_22_NO_5_ [M + H]^+^ 368.1492, found 368.1494.

***Diethyl 2-(5-chloro-3-oxo-2-phenylindolin-2-yl)malonate (*3b*).*** According to procedure A, **3b** was obtained as a yellow solid in 90% yield (36.1 mg; flash chromatographic condition: petroleum ether-acetone 90:10). ^1^H NMR (600 MHz, CDCl_3_) *δ* 7.53 (d, *J* = 2.2 Hz, 1H), 7.50 (t, *J* = 1.7 Hz, 1H), 7.49 (t, *J* = 1.7 Hz, 1H), 7.42 (dd, *J* = 8.7, 2.2 Hz, 1H), 7.33–7.30 (m, 2H), 7.27 (dt, *J* = 14.4, 1.1 Hz, 1H), 6.94 (d, *J* = 8.6 Hz, 1H), 6.13 (s, 1H), 4.70 (s, 1H), 4.09–4.01 (m, 3H), 3.96 (dq, *J* = 10.8, 7.1 Hz, 1H), 1.02 (t, *J* = 7.1 Hz, 3H), 0.95 (t, *J* = 7.1 Hz, 3H); ^13^C NMR (151 MHz, CDCl_3_) *δ* 196.0 (C=O), 166.8 (C=O), 165.2 (C=O), 157.4 (Cq), 136.3 (CH), 135.4 (Cq), 128.1 (CH, 2C), 127.5 (CH), 124.4 (CH, 2C), 123.8 (CH), 123.4 (Cq), 119.8 (Cq), 111.7 (CH), 70.0 (Cq), 61.2 (CH_2_), 60.9 (CH_2_), 57.8 (CH), 12.9 (CH_3_), 12.6 (CH_3_); HR-ESIMS *m*/*z* calcd for C_21_H_21_ClNO_5_ [M + H]^+^ 402.1103, found 402.1103.

***Diethyl 2-(5-methyl-3-oxo-2-phenylindolin-2-yl)malonate (*3c*).*** According to procedure A, **3c** was obtained as a yellow solid in 95% yield (36.2 mg; flash chromatographic condition: petroleum ether-acetone 90:10). ^1^H NMR (600 MHz, CDCl_3_) *δ* 7.52–7.48 (m, 2H), 7.36 (s, 1H), 7.32–7.28 (m, 3H), 7.25 (d, *J* = 7.3 Hz, 1H), 6.90 (d, *J* = 8.3 Hz, 1H), 5.92 (s, 1H), 4.71 (s, 1H), 4.10–3.99 (m, 3H), 3.92 (dq, *J* = 10.8, 7.1 Hz, 1H), 2.27 (s, 3H), 1.02 (t, *J* = 7.1 Hz, 3H), 0.89 (t, *J* = 7.1 Hz, 3H); ^13^C NMR (151 MHz, CDCl_3_) *δ* 198.2 (C=O), 167.9 (C=O), 166.5 (C=O), 158.7 (Cq), 138.8 (CH), 137.2 (Cq), 128.9 (CH, 2C), 128.8 (Cq), 128.1 (CH), 125.4 (CH, 2C), 124.9 (CH), 119.8 (Cq), 111.5 (CH), 70.8 (Cq), 62.0 (CH_2_), 61.7 (CH_2_), 58.9 (CH), 20.6 (CH_3_), 13.9 (CH_3_), 13.5 (CH_3_); HR-ESIMS *m*/*z* calcd for C_22_H_24_NO_5_ [M + H]^+^ 382.1649, found 382.1650.

***Diethyl 2-(5-methoxy-3-oxo-2-phenylindolin-2-yl)malonate (*3d*).*** According to procedure A, **3d** was obtained as a yellow solid in 98% yield (38.9 mg; flash chromatographic condition: petroleum ether-acetone 90:10). ^1^H NMR (600 MHz, CDCl_3_) *δ* 7.52–7.48 (m, 2H), 7.36 (s, 1H), 7.32–7.28 (m, 3H), 7.25 (d, *J* = 7.3 Hz, 1H), 6.90 (d, *J* = 8.3 Hz, 1H), 5.92 (s, 1H), 4.71 (s, 1H), 4.10–3.99 (m, 3H), 3.92 (dq, *J* = 10.8, 7.1 Hz, 1H), 2.27 (s, 3H), 1.02 (t, *J* = 7.1 Hz, 3H), 0.89 (t, *J* = 7.1 Hz, 3H); ^13^C NMR (151 MHz, CDCl_3_) *δ* 198.1 (C=O), 167.6 (C=O), 166.2 (C=O), 155.8 (Cq), 153.4 (Cq), 136.9 (Cq), 128.7 (CH, 2C), 127.9 (CH), 127.6 (CH), 125.2 (CH, 2C), 119.6 (Cq), 112.9 (CH), 105.3 (CH), 71.1 (Cq), 61.8 (CH_2_), 61.5 (CH_2_), 58.7 (CH_3_), 55.6 (CH), 13.6 (CH_3_), 13.4 (CH_3_); HR-ESIMS *m*/*z* calcd for C_22_H_24_NO_6_ [M + H]^+^ 398.1598, found 398.1600.

***Diethyl 2-(6-methyl-3-oxo-2-phenylindolin-2-yl)malonate (*3e*).*** According to procedure A, **3e** was obtained as a yellow solid in 94% yield (35.8 mg; flash chromatographic condition: petroleum ether-acetone 90:10). ^1^H NMR (600 MHz, CDCl_3_) *δ* 7.51 (d, *J* = 7.6 Hz, 2H), 7.45 (d, *J* = 7.9 Hz, 1H), 7.29 (t, *J* = 7.6 Hz, 2H), 7.24 (t, *J* = 7.3 Hz, 1H), 6.78 (s, 1H), 6.64 (d, *J* = 7.9 Hz, 1H), 6.00 (s, 1H), 4.70 (s, 1H), 4.09–3.98 (m, 3H), 3.92 (dq, *J* = 10.8, 7.1 Hz, 1H), 2.38 (s, 3H), 1.02 (t, *J* = 7.1 Hz, 3H), 0.89 (t, *J* = 7.1 Hz, 3H); ^13^C NMR (151 MHz, CDCl_3_) *δ* 197.4 (C=O), 167.9 (C=O), 166.4 (C=O), 160.7 (Cq), 149.1 (Cq), 137.3 (Cq), 128.9 (CH, 2C), 128.1 (CH), 125.4 (CH, 2C), 125.3 (CH), 121.0 (CH), 117.4 (Cq), 111.6 (CH), 70.6 (Cq), 62.0 (CH_2_), 61.7 (CH_2_), 58.7 (CH), 22.6 (CH_3_), 13.8 (CH_3_), 13.5 (CH_3_); HR-ESIMS *m/z* calcd for C_22_H_24_NO_5_ [M + H]^+^ 382.1649, found 382.1648. 

***Diethyl 2-(7-methyl-3-oxo-2-phenylindolin-2-yl)malonate (*3f*).*** According to procedure A, **3f** was obtained as a yellow solid in 91% yield (34.6 mg; flash chromatographic condition: petroleum ether-acetone 90:10). ^1^H NMR (600 MHz, CDCl_3_) *δ* 7.51 (d, *J* = 7.5 Hz, 2H), 7.43 (d, *J* = 7.7 Hz, 1H), 7.34–7.29 (m, 3H), 7.25 (t, *J* = 7.3 Hz, 1H), 6.76 (t, *J* = 7.4 Hz, 1H), 5.87 (s, 1H), 4.72 (s, 1H), 4.04 (m, 3H), 3.88 (dq, *J* = 10.7, 7.1 Hz, 1H), 2.35 (s, 3H), 1.05 (t, *J* = 7.1 Hz, 3H), 0.84 (t, *J* = 7.1 Hz, 3H); ^13^C NMR (151 MHz, CDCl_3_) *δ* 198.4 (C=O), 168.0 (C=O), 166.3 (C=O), 159.4 (Cq), 137.4 (CH), 137.1 (Cq), 128.9 (CH, 2C), 128.2 (CH), 125.4 (CH, 2C), 122.9 (CH), 120.7 (Cq), 119.4 (CH), 119.1 (Cq), 70.6 (Cq), 62.0 (CH_2_), 61.8 (CH_2_), 58.8 (CH), 15.9 (CH_3_), 13.9 (CH_3_), 13.4 (CH_3_); HR-ESIMS *m/z* calcd for C_22_H_24_NO_5_ [M + H]^+^ 382.1649, found 382.1649.

***Diethyl 2-(2-(4-fluorophenyl)-3-oxoindolin-2-yl)malonate (*3g*).*** According to procedure A, **3g** was obtained as a yellow solid in 90% yield (34,6 mg; flash chromatographic condition: petroleum ether-acetone 90:10). ^1^H NMR (600 MHz, CDCl_3_) *δ* 7.57 (d, *J* = 7.7 Hz, 1H), 7.56–7.52 (m, 2H), 7.50–7.46 (m, 1H), 7.03–6.95 (m, 3H), 6.83 (t, *J* = 7.4 Hz, 1H), 6.10 (s, 1H), 4.64 (s, 1H), 4.12–3.97 (m, 3H), 3.91 (dq, *J* = 10.8, 7.1 Hz, 1H), 1.06 (t, *J* = 7.1 Hz, 3H), 0.86 (t, *J* = 7.1 Hz, 4H); ^13^C NMR (151 MHz, CDCl_3_) *δ* 198.1 (C=O), 167.8 (C=O), 166.1 (C=O), 163.6 (Cq), 161.9 (Cq), 160.1 (Cq), 137.6 (CH), 132.8 (Cq), 132.8 (Cq), 127.5 (CH, 2C), 127.4 (CH, 2C), 125.6 (CH), 119.6 (Cq), 119.4 (CH), 115.9 (CH, 2C), 115.8 (CH, 2C), 111.6 (CH), 69.8 (Cq), 62.2 (CH_2_), 61.9 (CH_2_), 58.9 (CH), 13.9 (CH_3_), 13.4 (CH_3_); HR-ESIMS *m/z* calcd for C_21_H_21_FNO_5_ [M + H]^+^ 386.1398, found 386.1402.

***Diethyl 2-(3-oxo-2-(p-tolyl)indolin-2-yl)malonate (*3h*).*** According to procedure A, **3h** was obtained as a yellow solid in 99% yield (37.7 mg; flash chromatographic condition: petroleum ether-acetone 90:10). ^1^H NMR (600 MHz, CDCl_3_) *δ* 7.56 (d, *J* = 7.6 Hz, 1H), 7.46 (t, *J* = 7.6 Hz, 1H), 7.38 (d, *J* = 8.2 Hz, 2H), 7.11 (d, *J* = 8.1 Hz, 2H), 6.96 (d, *J* = 8.2 Hz, 1H), 6.80 (t, *J* = 7.4 Hz, 1H), 6.03 (s, 1H), 4.70 (s, 1H), 4.12–3.98 (m, 3H), 3.90 (dq, *J* = 10.8, 7.1 Hz, 1H), 2.28 (s, 3H), 1.06 (t, *J* = 7.1 Hz, 3H), 0.85 (t, *J* = 7.1 Hz, 3H); ^13^C NMR (151 MHz, CDCl_3_) *δ* 198.2 (C=O), 167.9 (C=O), 166.5 (C=O), 160.2 (Cq), 137.9 (Cq), 137.3 (CH), 133.9 (Cq), 129.7 (CH, 2C), 125.6 (CH), 125.2 (CH, 2C), 119.7 (Cq), 119.2 (CH), 111.5 (CH), 70.3 (Cq), 62.0 (CH_2_), 61.7 (CH_2_), 58.7 (CH), 21.0 (CH_3_), 13.9 (CH_3_), 13.4 (CH_3_); HR-ESIMS *m/z* calcd for C_22_H_24_NO_5_ [M + H]^+^ 382.1649, found 382.1651.

***Diethyl 2-(3-oxo-2-(4-(trifluoromethoxy)phenyl)indolin-2-yl)malonate (*3i*).*** According to procedure A, **3i** was obtained as a yellow solid in 83% yield (37.4 mg; flash chromatographic condition: petroleum ether-acetone 90:10). ^1^H NMR (600 MHz, CDCl_3_) *δ* 7.63–7.60 (m, 2H), 7.58 (d, *J* = 7.7 Hz, 1H), 7.51–7.47 (m, 1H), 7.16 (d, *J* = 8.3 Hz, 2H), 6.98 (d, *J* = 8.2 Hz, 1H), 6.84 (t, *J* = 7.4 Hz, 1H), 6.11 (s, 1H), 4.64 (s, 1H), 4.09–4.03 (m, 2H), 4.00 (ddd, *J* = 14.3, 9.0, 5.4 Hz, 1H), 3.91 (dq, *J* = 10.8, 7.1 Hz, 1H), 1.03 (t, *J* = 7.1 Hz, 3H), 0.87 (t, *J* = 7.1 Hz, 3H); ^13^C NMR (151 MHz, CDCl_3_) *δ* 197.9 (C=O), 167.8 (C=O), 165.9 (C=O), 160.1 (Cq), 149.2 (Cq) 137.7 (CH), 135.9 (Cq), 127.3 (CH, 2C), 125.6 (CH), 121.3 (Cq), 121.2 (CH, 2C), 119.6 (Cq), 119.5 (CH), 119.5 (Cq), 111.7 (CH), 69.8 (Cq), 62.2 (CH_2_), 61.9 (CH_2_), 58.9 (CH), 13.8 (CH_3_), 13.5 (CH_3_); HR-ESIMS *m/z* calcd for C_22_H_21_F_3_NO_6_ [M + H]^+^ 452.1315, found 452.1314.

***Diethyl 2-(2-(3-methoxyphenyl)-3-oxoindolin-2-yl)malonate (*3j*).*** According to procedure A, **3j** was obtained as a yellow solid in 99% yield (39.3 mg; flash chromatographic condition: petroleum ether-acetone 90:10). ^1^H NMR (600 MHz, CDCl_3_) *δ* 7.56 (d, *J* = 7.7 Hz, 1H), 7.47 (ddd, *J* = 8.3, 7.2, 1.3 Hz, 1H), 7.22 (t, *J* = 8.0 Hz, 1H), 7.09 (ddd, *J* = 7.9, 1.8, 0.8 Hz, 1H), 7.07–7.05 (m, 1H), 6.96 (d, *J* = 8.2 Hz, 1H), 6.83–6.77 (m, 2H), 6.04 (s, 1H), 4.70 (s, 1H), 4.13–3.98 (m, 3H), 3.90 (dq, *J* = 10.7, 7.2 Hz, 1H), 3.77 (s, 3H), 1.06 (t, *J* = 7.1 Hz, 3H), 0.85 (t, *J* = 7.1 Hz, 3H); ^13^C NMR (151 MHz, CDCl_3_) *δ* 197.9 (C=O), 167.9 (C=O), 166.4 (C=O), 160.2 (Cq), 159.9 (Cq), 138.6 (Cq), 137.4 (CH), 129.9 (CH), 125.5 (CH), 119.6 (Cq), 119.3 (CH), 117.7 (CH), 113.3 (CH), 111.6 (CH), 111.5 (CH), 70.3 (Cq), 62.0 (CH_2_), 61.8 (CH_2_), 58.7 (CH_3_), 55.3 (CH), 13.9 (CH_3_), 13.4 (CH_3_); HR-ESIMS *m/z* calcd for C_22_H_24_NO_6_ [M + H]^+^ 398.1598, found 398.1599.

***Diethyl 2-(2-methyl-3-oxoindolin-2-yl)malonate (*3k*).*** According to procedure A, **3k** was obtained as a yellow solid in 84% yield (25.6 mg; flash chromatographic condition: petroleum ether-acetone 90:10). ^1^H NMR (600 MHz, CDCl_3_) *δ* 7.63 (d, *J* = 7.7 Hz, 1H), 7.45–7.41 (m, 1H), 6.85–6.79 (m, 2H), 5.45 (s, 1H), 4.36–4.27 (m, 2H), 3.98 (s, 1H), 3.97–3.93 (m, 1H), 3.89–3.83 (m, 1H), 1.35 (s, 3H), 1.33 (t, *J* = 7.1 Hz, 3H), 0.85 (t, *J* = 7.1 Hz, 3H); ^13^C NMR (151 MHz, CDCl_3_) *δ* 201.6 (C=O), 168.4 (C=O), 166.5 (C=O), 159.8 (Cq), 137.2 (CH), 125.0 (CH), 120.1 (Cq), 119.0 (CH), 112.3 (CH), 65.3 (Cq), 61.9 (CH_2_), 61.9 (CH_2_), 57.8 (CH), 22.3 (CH_3_), 14.2 (CH_3_), 13.4 (CH_3_); HR-ESIMS *m/z* calcd for C_16_H_20_NO_5_ [M + H]^+^ 306.1336, found 306.1335.

***Dimethyl 2-(3-oxo-2-phenylindolin-2-yl)malonate (*3l*).*** According to procedure A, **3l** was obtained as a yellow solid in 97% yield (32.8 mg; flash chromatographic condition: petroleum ether-acetone 90:10). ^1^H NMR (600 MHz, CDCl_3_) *δ* 7.57 (d, *J* = 7.7 Hz, 1H), 7.53–7.50 (m, 2H), 7.50–7.46 (m, 1H), 7.31 (t, *J* = 7.6 Hz, 2H), 7.26 (dd, *J* = 7.9, 5.9 Hz, 1H), 6.98 (d, *J* = 8.2 Hz, 1H), 6.82 (t, *J* = 7.4 Hz, 1H), 6.08 (s, 1H), 4.76 (s, 1H), 3.58 (s, 3H), 3.49 (s, 3H). ^13^C NMR (151 MHz, CDCl_3_) *δ* 198.1 (C=O), 168.4 (C=O), 166.6 (C=O), 160.2 (Cq), 137.5 (CH), 136.8 (Cq), 129.0 (CH, 2C), 128.3 (CH), 125.6 (CH), 125.3 (CH, 2C), 119.4 (Cq), 119.4 (CH), 111.6 (CH), 70.4 (Cq), 58.6 (CH), 52.8 (CH_3_, 2C); HR-ESIMS *m/z* calcd for C_19_H_18_NO_5_ [M + H]^+^ 340.1179, found 340.1181.

***Diisopropyl 2-(3-oxo-2-phenylindolin-2-yl)malonate (*3m*).*** According to procedure A, **3m** was obtained as a yellow solid in 95% yield (37.5 mg; flash chromatographic condition: petroleum ether-acetone 90:10). ^1^H NMR (600 MHz, CDCl_3_) *δ* 7.56 (d, *J* = 7.7 Hz, 1H), 7.53–7.50 (m, 2H), 7.46 (t, *J* = 7.7 Hz, 1H), 7.29 (t, *J* = 7.6 Hz, 2H), 7.24 (t, *J* = 7.3 Hz, 1H), 6.96 (d, *J* = 8.2 Hz, 1H), 6.80 (t, *J* = 7.4 Hz, 1H), 6.09 (s, 1H), 4.89–4.82 (m, 2H), 4.66 (s, 1H), 1.09 (d, *J* = 6.3 Hz, 3H), 1.06 (d, *J* = 6.3 Hz, 3H), 0.98 (d, *J* = 6.3 Hz, 3H), 0.72 (d, *J* = 6.3 Hz, 3H); ^13^C NMR (151 MHz, CDCl_3_) *δ* 198.0 (C=O), 167.5 (C=O), 165.9 (C=O), 160.2 (Cq), 137.3 (CH), 137.2 (Cq), 128.9 (CH, 2C), 128.1 (CH), 125.5 (CH), 125.4 (CH, 2C), 119.8 (Cq), 119.2 (CH), 111.5 (CH), 70.4 (CH), 70.1 (CH), 69.4 (Cq), 59.1 (CH), 21.5 (CH_3_), 21.4 (CH_3_), 21.3 (CH_3_), 20.7 (CH_3_); HR-ESIMS *m/z* calcd for C_23_H_26_NO_5_ [M + H]^+^ 396.1805, found 396.1803.

***Di-tert-butyl 2-(3-oxo-2-phenylindolin-2-yl)malonate (*3n*).*** According to procedure A, **3n** was obtained as a yellow solid in 96% yield (32.5 mg; flash chromatographic condition: petroleum ether-acetone 90:10). ^1^H NMR (600 MHz, CDCl_3_) *δ* 7.56 (d, *J* = 7.6 Hz, 1H), 7.53 (d, *J* = 7.6 Hz, 2H), 7.48 (ddd, *J* = 8.3, 7.2, 1.3 Hz, 1H), 7.31 (t, *J* = 7.6 Hz, 2H), 7.26–7.23 (m, 1H), 6.96 (d, *J* = 8.2 Hz, 1H), 6.81 (t, *J* = 7.3 Hz, 1H), 6.07 (s, 1H), 4.56 (s, 1H), 1.23 (s, 9H), 1.14 (s, 9H); ^13^C NMR (151 MHz, CDCl_3_) *δ* 197.9 (C=O), 167.0 (C=O), 165.6 (C=O), 160.1 (Cq), 137.4 (Cq), 137.2 (CH), 128.6 (2C, CH), 127.8 (CH), 125.5 (CH), 125.4 (2C, CH), 119.6 (Cq), 118.9 (CH), 111.2 (CH), 83.1 (Cq), 82.3(Cq), 70.5(Cq), 60.4 (CH), 27.5 (CH_3_, 3C), 27.4 (CH_3_, 3C); HR-ESIMS *m/z* calcd for C_25_H_30_NO_5_ [M + H]^+^ 424.2118, found 424.2122.

***Dibutyl 2-(3-oxo-2-phenylindolin-2-yl)malonate (*3o*).*** According to procedure A, **3o** was obtained as a yellow solid in 99% yield (41.9 mg; flash chromatographic condition: petroleum ether-acetone 90:10). ^1^H NMR (600 MHz, CDCl_3_) *δ* 7.55 (d, *J* = 7.7 Hz, 1H), 7.53 (dd, *J* = 8.2, 0.9 Hz, 2H), 7.48–7.45 (m, 1H), 7.30 (t, *J* = 7.6 Hz, 2H), 7.25 (t, *J* = 7.3 Hz, 1H), 6.97 (d, *J* = 8.2 Hz, 1H), 6.81 (t, *J* = 7.4 Hz, 1H), 6.09 (s, 1H), 4.74 (s, 1H), 4.02–3.96 (m, 2H), 3.96–3.87 (m, 2H), 1.45–1.35 (m, 2H), 1.27–1.10 (m, 6H), 0.83 (t, *J* = 7.4 Hz, 3H), 0.78 (t, *J* = 7.2 Hz, 3H); ^13^C NMR (151 MHz, CDCl_3_) *δ* 198.0 (C=O), 168.1 (C=O), 166.5 (C=O), 160.2 (Cq), 137.4 (CH), 137.0 (Cq), 128.9 (CH, 2C), 128.2 (CH), 125.6 (CH), 125.4 (CH, 2C), 119.6 (Cq), 119.2 (CH), 111.6 (CH), 70.3 (Cq), 65.9 (CH_2_), 65.5 (CH_2_), 58.8 (CH), 30.4 (CH_2_), 30.1 (CH_2_), 18.9 (CH_2_, 2C), 13.7 (CH_3_), 13.6 (CH_3_); HR-ESIMS *m/z* calcd for C_25_H_30_NO_5_ [M + H]^+^ 424.2118, found 424.2120.

***Dibenzyl 2-(3-oxo-2-phenylindolin-2-yl)malonate (*3p*).*** According to procedure A, **3p** was obtained as a yellow solid in 99% yield (48.6 mg; flash chromatographic condition: petroleum ether-acetone 90:10). ^1^H NMR (600 MHz, CDCl_3_) *δ* 7.46 (dd, *J* = 7.8, 1.6 Hz, 2H), 7.43 (d, *J* = 7.7 Hz, 1H), 7.40 (ddd, *J* = 8.3, 7.2, 1.3 Hz, 1H), 7.29–7.18 (m, 9H), 7.01 (t, *J* = 6.9 Hz, 4H), 6.85 (d, *J* = 8.2 Hz, 1H), 6.75–6.71 (m, 1H), 6.01 (s, 1H), 4.97 (s, 2H), 4.93 (d, *J* = 12.2 Hz, 1H), 4.89 (d, *J* = 12.2 Hz, 1H), 4.84 (s, 1H); ^13^C NMR (151 MHz, CDCl_3_) *δ* 197.7 (C=O), 167.8 (C=O), 166.1 (C=O), 160.0 (Cq), 137.3 (CH), 136.8 (Cq), 134.9 (Cq), 134.6 (Cq), 129.0 (CH, 2C), 128.6 (CH, 2C), 128.5 (CH, 2C), 128.4 (CH), 128.3 (CH, 2C), 128.3 (CH), 128.2 (CH), 128.2 (CH, 2C), 125.6 (CH), 125.4 (CH, 2C), 119.4 (Cq), 119.3 (CH), 111.5 (CH), 70.4 (Cq), 67.8 (CH_2_), 67.5 (CH_2_), 58.8 (CH); HR-ESIMS *m/z* calcd for C_31_H_26_NO_5_ [M + H]^+^ 492.1805, found 492.1807.

***3-(3-oxo-2-phenylindolin-2-yl)pentane-2,4-dione (*3q*).*** According to procedure A, **3q** was obtained as a yellow solid in 80% yield (24.6 mg; flash chromatographic condition: petroleum ether-acetone 90:10). ^1^H NMR (600 MHz, CDCl_3_) *δ* 7.61 (d, *J* = 7.4 Hz, 2H), 7.53 (d, *J* = 7.7 Hz, 1H), 7.48 (ddd, *J* = 8.3, 7.2, 1.2 Hz, 1H), 7.32 (t, *J* = 7.7 Hz, 2H), 7.25 (d, *J* = 7.3 Hz, 1H), 6.99 (d, *J* = 8.3 Hz, 1H), 6.80 (t, *J* = 7.2 Hz, 1H), 6.28 (s, 1H), 5.08 (s, 1H), 2.14 (s, 3H), 2.05 (s, 3H); ^13^C NMR (151 MHz, CDCl_3_) *δ* 203.6 (C=O), 200.1 (C=O), 199.6 (C=O), 160.8 (Cq), 138.1 (CH), 137.5 (Cq), 129.0 (CH, 2C), 128.2 (CH), 125.5 (CH, 2C), 125.4 (CH), 119.4 (CH), 119.2 (Cq), 112.3 (CH), 71.2 (Cq), 71.1 (CH), 33.1 (CH_3_), 31.3 (CH_3_); HR-ESIMS *m/z* calcd for C_19_H_18_NO_3_ [M + H]^+^ 308.1281, found 308.1280.

***2-(1H-Indol-3-yl)-2-phenylindolin-3-one (*5a*)*.** According to procedure B, **5a** was obtained as a yellow solid in 98% yield (31.8 mg; flash chromatographic condition: petroleum ether-acetone 85:15). ^1^H NMR (600 MHz, acetone-*d*_6_) *δ* 10.28 (s, 1H), 7.61 (d, *J* = 7.6 Hz, 2H), 7.57 (d, *J* = 7.7 Hz, 1H), 7.53 (ddd, *J* = 8.4, 7.1, 1.4 Hz, 1H), 7.43 (d, *J* = 8.2 Hz, 1H), 7.36–7.23 (m, 4H), 7.21 (s, 1H), 7.16 (d, *J* = 8.0 Hz, 1H), 7.08 (t, *J* = 9.0 Hz, 2H), 6.90–6.79 (m, 2H); ^13^C NMR (151 MHz, acetone-*d*_6_) *δ* 200.9(C=O), 161.9(Cq), 141.4(Cq), 138.3(CH), 138.2(Cq), 128.9(CH, 2C), 128.2(CH), 127.7(CH, 2C), 126.8(Cq), 125.5(CH), 124.9(CH), 122.5(CH), 121.0(CH), 119.8(CH), 119.6(Cq), 119.0(CH), 116.3(Cq), 113.3(CH), 112.6(CH), 71.9(Cq); HR-ESIMS *m/z* calcd for C_22_H_17_N_2_O [M + H]^+^ 325.1335, found 325.1337.

***5-Chloro-2-(1H-indol-3-yl)-2-phenylindolin-3-one (*5b*)*.** According to procedure B, **5b** was obtained as a yellow solid in 90% yield (32.3 mg; flash chromatographic condition: petroleum ether-acetone 85:15). ^1^H NMR (600 MHz, CDCl_3_) *δ* 8.25 (s, 1H), 7.64 (d, *J* = 2.2 Hz, 1H), 7.57–7.51 (m, 2H), 7.44 (dd, *J* = 8.7, 2.2 Hz, 1H), 7.36 (d, *J* = 8.2 Hz, 1H), 7.33–7.28 (m, 3H), 7.18 (t, *J* = 7.6 Hz, 1H), 7.13 (d, *J* = 8.0 Hz, 1H), 7.09 (d, *J* = 2.5 Hz, 1H), 6.99 (t, *J* = 7.6 Hz, 1H), 6.86 (d, *J* = 8.7 Hz, 1H), 5.43 (s, 1H); ^13^C NMR (151 MHz, CDCl_3_) *δ* 199.6 (C=O), 158.9 (Cq), 139.1 (Cq), 137.6 (CH), 137.1 (Cq), 128.7 (CH, 2C), 128.1 (CH), 126.8 (CH, 2C), 125.6 (Cq), 124.9 (CH), 124.9 (Cq), 123.9 (CH), 122.8 (CH), 120.7 (Cq), 120.3 (CH), 119.7 (CH), 115.3 (Cq), 114.2 (CH), 111.9 (CH), 72.3 (Cq); HR-ESIMS *m/z* calcd for C_22_H_16_ClN_2_O [M + H]^+^ 359.0946, found 359.0950.

***2-(1H-Indol-3-yl)-5-methyl-2-phenylindolin-3-one (*5c*)*.** According to procedure B, **5c** was obtained as a yellow solid in 95% yield (32.2 mg; flash chromatographic condition: petroleum ether-acetone 85:15). ^1^H NMR (600 MHz, CDCl_3_) *δ* 8.16 (s, 1H), 7.56 (dt, *J* = 3.8, 2.1 Hz, 2H), 7.50 (s, 1H), 7.38 (d, *J* = 8.2 Hz, 1H), 7.35 (dd, *J* = 8.3, 1.7 Hz, 1H), 7.32–7.27 (m, 3H), 7.20–7.15 (m, 3H), 6.99 (dd, *J* = 11.2, 4.0 Hz, 1H), 6.87 (d, *J* = 8.3 Hz, 1H), 5.23 (s, 1H), 2.33 (s, 3H); ^13^C NMR (151 MHz, CDCl_3_) *δ* 200.9 (C=O), 159.2 (Cq), 139.8(Cq), 139.1 (CH), 137.0 (Cq), 129.4 (Cq), 128.5 (CH, 2C), 127.8 (CH), 126.9 (CH, 2C), 125.8 (Cq), 125.0 (CH), 123.9 (CH), 122.6 (CH), 120.1(CH), 119.9 (Cq), 119.9 (CH), 115.8 (Cq), 113.1 (CH), 111.8 (CH), 71.8 (Cq), 20.7 (CH_3_); HR-ESIMS *m/z* calcd for C_23_H_19_N_2_O [M + H]^+^ 339.1492, found 339.1495.

***2-(1H-Indol-3-yl)-5-methoxy-2-phenylindolin-3-one (*5d*)*.** According to procedure B, **5d** was obtained as a yellow solid in 98% yield (34.6 mg; flash chromatographic condition: petroleum ether-acetone 85:15). ^1^H NMR (600 MHz, CDCl_3_) *δ* 8.27 (s, 1H), 7.56 (dd, *J* = 8.1, 1.7 Hz, 2H), 7.34 (d, *J* = 8.3 Hz, 1H), 7.32–7.24 (m, 3H), 7.21–7.09 (m, 5H), 6.97 (t, *J* = 7.5 Hz, 1H), 6.89 (d, *J* = 8.8 Hz, 1H), 5.13 (s, 1H), 3.77 (s, 3H); ^13^C NMR (151 MHz, CDCl_3_) *δ* 201.2 (C=O), 156.6 (Cq), 154.0 (Cq), 139.8 (Cq), 137.0 (Cq), 128.5 (CH, 2C), 128.3 (CH), 127.8 (CH), 126.9 (CH, 2C), 125.7 (Cq), 123.8 (Cq), 122.6 (CH), 120.1 (CH), 120.0 (CH), 119.8(CH), 115.8 (Cq), 114.8 (CH), 111.8 (CH), 105.2 (CH), 72.4 (Cq), 55.9 (CH_3_); HR-ESIMS *m/z* calcd for C_23_H_19_N_2_O_2_ [M + H]^+^ 355.1441, found 355.1443.

***2-(1H-Indol-3-yl)-6-methyl-2-phenylindolin-3-one (*5e*)*.** According to procedure B, **5e** was obtained as a yellow solid in 94% yield (31.8 mg; flash chromatographic condition: petroleum ether-acetone 85:15). ^1^H NMR (600 MHz, acetone-*d*_6_) *δ* 10.24 (s, 1H), 7.61–7.56 (m, 2H), 7.43 (d, *J* = 8.1 Hz, 1H), 7.41 (d, *J* = 8.2 Hz, 1H), 7.33–7.24 (m, 3H), 7.19–7.12 (m, 3H), 7.07 (ddd, *J* = 8.2, 7.0, 1.1 Hz, 1H), 6.89–6.81 (m, 2H), 6.65 (dd, *J* = 7.9, 1.1 Hz, 1H), 2.34 (s, 3H); ^13^C NMR (151 MHz, acetone-*d*_6_) *δ* 200.1 (C=O), 162.4 (Cq), 149.6 (Cq), 141.8 (Cq), 138.3 (Cq), 128.9 (CH, 2C), 128.2 (CH), 127.8 (CH, 2C), 126.9 (Cq), 125.4 (CH), 125.0 (CH), 122.5 (CH), 121,1 (CH), 120.9 (CH), 119.8 (CH), 117.5 (Cq), 116.7 (Cq), 113.2 (CH), 112.5 (CH), 72.2 (Cq), 22.5 (CH_3_); HR-ESIMS *m/z* calcd for C_23_H_19_N_2_O [M + H]^+^ 339.1492, found 339.1494.

***2-(1H-Indol-3-yl)-7-methyl-2-phenylindolin-3-one (*5f*)*.** According to procedure B, **5f** was obtained as a yellow solid in 91% yield (30.8 mg; flash chromatographic condition: petroleum ether-acetone 85:15). ^1^H NMR (600 MHz, CDCl_3_) *δ* 8.19 (s, 1H), 7.62–7.53 (m, 3H), 7.38–7.25 (m, 5H), 7.21–7.13 (m, 3H), 6.99 (t, *J* = 7.5 Hz, 1H), 6.85 (t, *J* = 7.5 Hz, 1H), 5.12 (s, 1H), 2.27 (s, 3H); ^13^C NMR (151 MHz, CDCl_3_) *δ* 201.1(C=O), 159.8 (Cq), 139.7 (Cq), 137.5 (CH), 137.0 (Cq), 128.5 (CH, 2C), 127.8 (CH), 126.9 (CH, 2C), 125.8 (Cq), 124.0 (CH), 123.0 (CH), 122.5 (CH), 122.1 (Cq), 120.1 (CH), 119.9 (CH), 119.9 (CH), 119.3 (Cq), 115.8 (Cq), 111.7 (CH), 71.4 (Cq), 15.9 (CH_3_); HR-ESIMS *m/z* calcd for C_23_H_19_N_2_O [M + H]^+^ 339.1492, found 339.1496.

***2-(4-Fluorophenyl)-2-(1H-indol-3-yl)indolin-3-one (*5g*)*.** According to procedure B, **5g** was obtained as a yellow solid in 97% yield (33.2 mg; flash chromatographic condition: petroleum ether-acetone 85:15). ^1^H NMR (600 MHz, CDCl_3_) *δ* 8.29 (s, 1H), 7.70 (dd, *J* = 7.7, 1.3 Hz, 1H), 7.56–7.48 (m, 3H), 7.26 (s, 1H), 7.18 (ddd, *J* = 8.2, 7.0, 1.1 Hz, 1H), 7.16–7.09 (m, 2H), 7.03–6.94 (m, 3H), 6.94–6.88 (m, 2H), 5.37 (s, 1H); ^13^C NMR (151 MHz, CDCl_3_ ) *δ* 200.7 (C=O), 163.5 (Cq), 161.8 (Cq), 160.7 (Cq), 137.8 (CH), 137.1 (Cq), 135.4 (Cq), 135.4 (Cq), 128.8 (CH, 2C), 128.7 (CH, 2C), 125.7 (CH), 125.6 (Cq), 123.8 (CH), 122.8 (CH), 120.2 (CH), 120.0 (CH), 119.7 (CH), 119.6 (Cq), 115.5 (Cq), 115.4 (CH, 2C), 115.3 (CH, 2C), 113.2 (CH), 111.9 (CH), 70.9 (Cq); HR-ESIMS *m/z* calcd for C_22_H_16_FN_2_O [M + H]^+^ 343.1241, found 343.1238.

***2-(1H-Indol-3-yl)-2-methylindolin-3-one (*5h*)*.** According to procedure B, **5h** was obtained as a yellow solid in 95% yield (24.9 mg; flash chromatographic condition: petroleum ether-acetone 85:15). ^1^H NMR (600 MHz, acetone-*d*_6_) *δ* 10.20 (s, 1H), 7.57–7.49 (m, 2H), 7.43–7.34 (m, 3H), 7.06 (ddd, *J* = 8.0, 6.9, 1.1 Hz, 1H), 7.00 (d, *J* = 8.1 Hz, 1H), 6.87 (t, *J* = 7.5 Hz, 1H), 6.84–6.75 (m, 2H), 1.75 (s, 3H); ^13^C NMR (151 MHz, acetone-*d*_6_) *δ* 203.6 (C=O), 161.5 (Cq), 138.2(Cq), 138.0 (CH), 126.3 (Cq), 125.3 (CH), 123.8 (CH), 122.2 (CH), 121.0 (CH), 119.9 (Cq), 119.6 (CH), 118.5 (CH), 116.4 (Cq), 113.1 (CH), 112.3 (CH), 66.4(Cq), 24.1 (CH_3_); HR-ESIMS *m/z* calcd for C_17_H_15_N_2_O [M + H]^+^ 263.1179, found 263.1176.

***2-Ethyl-2-(1H-indol-3-yl)indolin-3-one (*5i*)*.** According to procedure B, **5i** was obtained as a yellow solid in 92% yield (25.3 mg; flash chromatographic condition: petroleum ether-acetone 85:15). ^1^H NMR (600 MHz, acetone-*d*_6_) *δ* 10.19 (s, 1H), 7.65 (d, *J* = 8.1 Hz, 1H), 7.55–7.45 (m, 2H), 7.41–7.33 (m, 2H), 7.11–6.99 (m, 2H), 6.92 (ddd, *J* = 8.1, 7.0, 1.1 Hz, 1H), 6.85 (s, 1H), 6.76 (ddd, *J* = 7.9, 7.1, 0.9 Hz, 1H), 2.35–2.30 (m, 1H), 2.26–2.21 (m, 1H), 0.89 (t, *J* = 7.4 Hz, 3H); ^13^C NMR (151 MHz, acetone-*d*_6_) *δ* 202.8 (C=O), 161.9 (Cq), 137.9 (Cq), 137.5 (CH), 126.0 (Cq), 124.6 (CH), 123.3 (CH), 121.9 (CH), 121.1 (CH), 120.6 (Cq), 119.3 (CH), 118.0 (CH), 115.2 (Cq), 112.4 (CH), 112.0 (CH), 70.1 (Cq), 30.5 (CH_2_), 8.1 (CH_3_); HR-ESIMS *m/z* calcd for C_18_H_17_N_2_O [M + H]^+^ 277.1335, found 277.1333.

***2-(Cyclopropylmethyl)-2-(1H-indol-3-yl)indolin-3-one (*5j*)*.** According to procedure B, **5j** was obtained as a yellow solid in 90% yield (27.2 mg; flash chromatographic condition: petroleum ether-acetone 85:15). ^1^H NMR (600 MHz, CDCl_3_) *δ* 8.25 (s, 1H), 7.66 (dd, *J* = 7.7, 1.3 Hz, 1H), 7.55 (d, *J* = 8.1 Hz, 1H), 7.51 (ddd, *J* = 8.4, 7.1, 1.4 Hz, 1H), 7.32 (d, *J* = 8.2 Hz, 1H), 7.18–7.13 (m, 2H), 7.03 (ddd, *J* = 8.1, 7.0, 1.0 Hz, 1H), 6.94 (d, *J* = 8.2 Hz, 1H), 6.87–6.83 (m, 1H), 5.20 (s, 1H), 2.57 (dd, *J* = 14.0, 4.7 Hz, 1H), 1.82 (dd, *J* = 14.0, 8.7 Hz, 1H), 0.81–0.72 (m, 1H), 0.41–0.29 (m, 2H), 0.19 (dq, *J* = 9.6, 4.9 Hz, 1H), 0.13–0.07 (m, 1H); ^13^C NMR (151 MHz, CDCl_3_) *δ* 203.6 (C=O), 160.8 (Cq), 137.5 (CH), 137.0 (Cq), 125.3 (Cq), 125.2 (CH), 122.7 (CH), 122.4 (CH), 120.9 (Cq), 120.3 (CH), 120.0 (CH), 119.0 (CH), 115.2 (Cq), 112.3 (CH), 111.6 (CH), 70.0 (Cq), 42.3 (CH_2_), 6.1 (CH_2_), 5.3 (CH_2_), 4.0 (CH); HR-ESIMS *m/z* calcd for C_20_H_19_N_2_O [M + H]^+^ 303.1492, found 303.1493.

***Ethyl 5-(2-(1H-indol-3-yl)-3-oxoindolin-2-yl)pentanoate (*5k*)*.** According to procedure B, **5k** was obtained as a yellow solid in 96% yield (36.1 mg; flash chromatographic condition: petroleum ether-acetone 85:15). ^1^H NMR (600 MHz, CDCl_3_) *δ* 8.49 (s, 1H), 7.65–7.61 (m, 1H), 7.49 (ddd, *J* = 8.3, 7.1, 1.4 Hz, 1H), 7.44 (d, *J* = 8.1 Hz, 1H), 7.30 (d, *J* = 9.0 Hz, 1H), 7.14 (ddd, *J* = 8.2, 7.0, 1.1 Hz, 1H), 7.05–6.98 (m, 2H), 6.90–6.81 (m, 2H), 5.10 (s, 1H), 4.09 (q, *J* = 7.1 Hz, 2H), 2.23 (m, 4H), 1.61 (p, *J* = 7.6 Hz, 2H), 1.45 (m, 1H), 1.25 (m, 1H), 1.21 (t, *J* = 7.1 Hz, 3H); ^13^C NMR (151 MHz, CDCl_3_ ) *δ* 203.5 (C=O), 173.8 (C=O), 160.9 (Cq), 137.7 (CH), 137.0 (Cq), 125.1 (CH), 125.0 (Cq), 122.8 (CH), 122.3 (CH), 120.7 (Cq), 120.0 (CH), 120.0 (CH), 119.0 (CH), 114.6 (Cq), 112.4 (CH), 111.8 (CH), 69.3(Cq), 60.4 (CH_2_), 37.0 (CH_2_), 34.2 (CH_2_), 25.2 (CH_2_), 23.1 (CH_2_), 14.3 (CH_3_); HR-ESIMS *m/z* calcd for C_23_H_25_N_2_O_3_ [M + H]^+^ 377.1860, found 377.1862.

***2-(4-Methyl-1H-indol-3-yl)-2-phenylindolin-3-one (*5l*)*.** According to procedure B, **5l** was obtained as a yellow solid in 98% yield (33.2 mg; flash chromatographic condition: petroleum ether-acetone 85:15). ^1^H NMR (600 MHz, CDCl_3_) *δ* 8.44 (s, 1H), 7.72 (dd, *J* = 7.8, 1.3 Hz, 1H), 7.53 (ddd, *J* = 8.4, 7.1, 1.4 Hz, 1H), 7.41–7.35 (m, 2H), 7.31–7.23 (m, 5H), 7.15–7.10 (m, 1H), 7.00–6.91 (m, 2H), 6.86 (dt, *J* = 7.2, 1.0 Hz, 1H), 5.33 (s, 1H), 2.09 (s, 3H); ^13^C NMR (151 MHz, CDCl_3_) *δ* 201.2 (C=O), 160.6 (Cq), 141.6 (Cq), 138.1 (Cq), 137.7 (CH), 129.7 (Cq), 128.7 (CH, 2C), 127.7 (CH), 126.6 (CH), 125.8 (CH, 2C), 125.1 (CH), 124.6 (Cq), 122.8 (CH), 122.5 (CH), 120.1 (CH), 119.8 (Cq), 114.3 (Cq), 113.5 (CH), 109.6 (CH), 72.4 (Cq), 21.9 (CH_3_); HR-ESIMS *m/z* calcd for C_23_H_19_N_2_O [M + H]^+^ 339.1492, found 339.1496.

***2-(5-Chloro-1H-indol-3-yl)-2-phenylindolin-3-one (*5m*)*.** According to procedure B, **5m** was obtained as a yellow solid in 90% yield (32.3 mg; flash chromatographic condition: petroleum ether-acetone 85:15). ^1^H NMR (600 MHz, CDCl_3_) *δ* 8.54 (s, 1H), 7.67 (d, *J* = 7.8 Hz, 1H), 7.57–7.47 (m, 3H), 7.32–7.27 (m, 3H), 7.22 (d, *J* = 8.6 Hz, 1H), 7.12 (s, 2H), 7.09 (dd, *J* = 8.5, 2.0 Hz, 1H), 6.94 (d, *J* = 8.3 Hz, 1H), 6.89 (t, *J* = 7.4 Hz, 1H), 5.43 (s, 1H); ^13^C NMR (151 MHz, CDCl_3_) *δ* 200.8 (C=O), 160.6 (Cq), 139.3 (CH), 137.9 (Cq), 135.5 (Cq), 128.7 (CH, 2C), 128.1 (CH), 126.8 (CH, 2C), 125.7 (CH), 125.3 (Cq), 123.0(CH), 119.9 (CH), 119.5 (Cq), 119.3 (CH), 115.3 (Cq), 113.0 (CH), 112.9 (Cq), 71.3 (Cq); HR-ESIMS *m/z* calcd for C_22_H_16_ClN_2_O [M + H]^+^ 359.0946, found 359.0949.

***2-(5-Methyl-1H-indol-3-yl)-2-phenylindolin-3-one (*5n*)*.** According to procedure B, **5n** was obtained as a yellow solid in 95% yield (32.2 mg; flash chromatographic condition: petroleum ether-acetone 85:15). ^1^H NMR (600 MHz, CDCl_3_) *δ* 8.21 (s, 1H), 7.70 (d, *J* = 7.8 Hz, 1H), 7.59–7.54 (m, 2H), 7.51 (ddd, *J* = 8.3, 7.1, 1.4 Hz, 1H), 7.34–7.28 (m, 3H), 7.25 (dd, *J* = 8.3, 2.9 Hz, 1H), 7.10 (d, *J* = 5.1 Hz, 1H), 7.01 (d, *J* = 8.3 Hz, 1H), 6.96 (s, 1H), 6.93 (d, *J* = 8.2 Hz, 1H), 6.90 (t, *J* = 7.4 Hz, 1H), 5.45 (s, 1H), 2.32 (s, 3H); ^13^C NMR (151 MHz, CDCl_3_) *δ* 200.8 (C=O), 160.8 (Cq), 139.6 (Cq), 137.6 (CH), 135.4 (Cq), 129.4 (CH), 128.5 (CH, 2C), 127.8 (CH), 126.9 (CH, 2C), 125.9 (Cq), 125.7 (CH), 124.2 (CH), 124.0 (Cq), 119.7 (CH), 119.6 (Cq), 119.3 (CH), 114.7 (Cq), 113.0 (CH), 111.5 (CH), 71.5 (Cq), 21.6 (CH_3_),; HR-ESIMS *m/z* calcd for C_23_H_19_N_2_O [M + H]^+^ 339.1492, found 339.1494.

***2-(5-Methoxy-1H-indol-3-yl)-2-phenylindolin-3-one (*5o*)*.** According to procedure B, **5o** was obtained as a yellow solid in 98% yield (34.8mg; flash chromatographic condition: petroleum ether-acetone 85:15). ^1^H NMR (600 MHz, CDCl_3_) ^1^H NMR (600 MHz, CDCl_3_) *δ* 8.14 (s, 1H), 7.70 (dd, *J* = 7.8, 1.3 Hz, 1H), 7.65–7.57 (m, 2H), 7.52 (ddd, *J* = 8.3, 7.1, 1.3 Hz, 1H), 7.36–7.23 (m, 4H), 7.08 (d, *J* = 5.8 Hz, 1H), 6.94 (d, *J* = 8.2 Hz, 1H), 6.90 (t, *J* = 7.4 Hz, 1H), 6.83 (dd, *J* = 8.6, 2.4 Hz, 1H), 6.57 (d, *J* = 2.4 Hz, 1H), 5.40 (s, 1H), 3.61 (s, 3H); ^13^C NMR (151 MHz, CDCl_3_) *δ* 200.8 (C=O), 160.5 (Cq), 153.9 (CH), 139.3 (CH), 137.5 (Cq), 132.0 (Cq), 128.3 (CH, 2C), 127.7 (CH), 126.8 (CH, 2C), 126.0 (CH), 125.4 (CH), 124.6 (Cq), 119.6 (Cq), 119.6 (CH), 115.4 (Cq), 112.8 (CH), 112.3 (Cq), 112.2 (CH), 101.8 (CH), 71.2 (Cq), 55.5 (CH_3_); HR-ESIMS *m/z* calcd for C_23_H_19_N_2_O_2_ [M + H]^+^ 355.1441, found 355.1444.

***2-(6-Methyl-1H-indol-3-yl)-2-phenylindolin-3-one (*5p*)*.** According to procedure B, **5p** was obtained as a yellow solid in 94% yield (31.9 mg; flash chromatographic condition: petroleum ether-acetone 85:15). ^1^H NMR (600 MHz, CDCl_3_) *δ* 8.11 (s, 1H), 7.70 (d, *J* = 7.8 Hz, 1H), 7.59–7.54 (m, 2H), 7.51 (ddd, *J* = 8.3, 7.1, 1.4 Hz, 1H), 7.33–7.27 (m, 3H), 7.16 (s, 1H), 7.08–7.02 (m, 2H), 6.94–6.87 (m, 2H), 6.83 (dd, *J* = 8.3, 1.3 Hz, 1H), 5.38 (s, 1H), 2.42 (s, 3H); ^13^C NMR (151 MHz, CDCl_3_) *δ* 200.8 (C=O), 160.7 (Cq), 139.7 (Cq), 137.6 (CH), 137.5 (Cq), 132.5 (Cq), 128.5 (CH, 2C), 127.8 (CH), 126.9 (CH, 2C), 125.7 (CH), 123.5 (Cq), 123.2 (Cq), 121.9 (CH), 119.7 (CH), 119.7 (CH), 119.4 (CH), 115.4 (Cq), 113.0 (CH), 111.7 (CH), 71.5 (Cq), 21.7 (CH_3_); HRMS *m/z* calcd for C_23_H_19_N_2_O [M + H]^+^ 339.1492, found 339.1494.

***2-(7-Methyl-1H-indol-3-yl)-2-phenylindolin-3-one (*5q*)*.** According to procedure B, **5q** was obtained as a yellow solid in 91% yield (30.8 mg; flash chromatographic condition: petroleum ether-acetone 85:15). ^1^H NMR (600 MHz, CDCl_3_) *δ* 8.28 (s, 1H), 7.71 (d, *J* = 7.7 Hz, 1H), 7.61–7.55 (m, 2H), 7.51 (ddd, *J* = 8.4, 7.1, 1.4 Hz, 1H), 7.32–7.27 (m, 3H), 7.13 (s, 1H), 7.01 (dd, *J* = 19.3, 7.5 Hz, 2H), 6.95–6.87 (m, 3H), 5.45 (s, 1H), 2.46 (s, 3H); ^13^C NMR (151 MHz, CDCl_3_) *δ* 200.8 (C=O), 160.7 (Cq), 139.6 (CH), 137.6 (Cq), 136.6 (Cq), 128.5 (CH, 2C), 127.8 (CH), 126.9 (CH, 2C), 125.7 (CH), 125.3 (CH), 123.6 (Cq), 123.1 (CH), 121.1 (Cq), 120.3 (CH), 119.7 (CH), 119.6 (Cq), 117.4 (CH), 115.9 (Cq), 113.0 (CH), 71.5 (Cq), 16.7 (CH_3_); HR-ESIMS *m/z* calcd for C_23_H_19_N_2_O [M + H]^+^ 339.1492, found 339.1495.

***2-(3-Methyl-1H-indol-2-yl)-2-phenylindolin-3-one (*5r*)*.** According to procedure C, **5r** was obtained as a yellow solid in 90% yield (30.4 mg; flash chromatographic condition: petroleum ether-acetone 85:15). ^1^H NMR (600 MHz, CDCl_3_) *δ* 8.84 (s, 1H), 7.69 (d, *J* = 7.7 Hz, 1H), 7.59–7.53 (m, 2H), 7.36–7.29 (m, 6H), 7.20 (ddd, *J* = 8.2, 7.0, 1.2 Hz, 1H), 7.13 (t, *J* = 7.4 Hz, 1H), 7.01 (d, *J* = 8.3 Hz, 1H), 6.93 (t, *J* = 7.4 Hz, 1H), 5.43 (s, 1H), 2.22 (s, 3H); ^13^C NMR (151 MHz, CDCl_3_) *δ* 201.1 (C=O), 161.0 (Cq), 139.6 (Cq), 138.3 (CH), 134.6 (Cq), 131.0 (Cq), 129.6 (Cq), 129.0 (CH, 2C), 128.5 (CH), 126.5 (CH, 2C), 125.8 (CH), 122.4 (CH), 120.3 (CH), 119.6 (Cq), 119.5 (CH), 118.6 (CH), 112.9 (CH), 111.2 (CH), 109.7 (Cq), 71.6 (Cq), 9.6 (CH_3_); HR-ESIMS *m/z* calcd for C_23_H_19_N_2_O [M + H]^+^ 339.1492, found 339.1490.

***N-(2-(5-Methoxy-2-(3-oxo-2-phenylindolin-2-yl)-1H-indol-3-yl)ethyl)acetamide (*5s*)*.** According to procedure C, **5s** was obtained as a yellow solid in 92% yield (40.3 mg; flash chromatographic condition: petroleum ether-acetone 60:40). ^1^H NMR (600 MHz, CDCl_3_) *δ* 9.35 (s, 1H), 8.07 (s, 1H), 7.53 (d, *J* = 7.8 Hz, 1H), 7.43 (ddd, *J* = 8.3, 7.0, 1.3 Hz, 1H), 7.26–7.14 (m, 6H), 7.07 (d, *J* = 8.3 Hz, 1H), 6.89 (d, *J* = 2.4 Hz, 1H), 6.80 (dd, *J* = 8.8, 2.4 Hz, 1H), 6.72 (ddd, *J* = 7.8, 7.0, 0.8 Hz, 1H), 6.13 (s, 1H), 3.78 (s, 3H), 3.47–3.31 (m, 1H), 3.19–3.13 (m, 1H), 2.84–2.79 (m, 1H), 2.75–2.70 (m, 1H), 1.90 (s, 3H); ^13^C NMR (151 MHz, CDCl_3_) *δ* 201.4 (C=O), 171.7 (C=O), 162.3 (Cq), 154.2 (Cq), 140.0 (Cq), 138.6 (CH), 133.1 (Cq), 129.7 (Cq), 129.0 (Cq), 128.9(CH, 2C), 128.2 (CH), 126.0(CH, 2C), 125.6 (CH), 118.8 (CH), 117.3 (Cq), 112.4 (CH), 112.3 (CH), 112.2 (CH), 110.0 (Cq), 100.2 (CH), 71.1 (Cq), 56.1 (CH_3_), 41.2 (CH_2_), 24.6 (CH_2_), 23.3 (CH_3_); HR-ESIMS *m/z* calcd for C_27_H_26_N_3_O_3_ [M + H]^+^ 440.1969, found 440.1965.

***Methyl (2-(2-(3-oxo-2-phenylindolin-2-yl)-1H-indol-3-yl)ethyl)carbamate (*5t*)*.** According to procedure C, **5t** was obtained as a yellow solid in 90% yield (38.2 mg; flash chromatographic condition: petroleum ether-acetone 60:40). ^1^H NMR (600 MHz, CDCl_3_) *δ* 9.46 (s, 1H), 7.74 (s, 1H), 7.61 (d, *J* = 7.8 Hz, 1H), 7.56–7.46 (m, 2H), 7.37 (d, *J* = 8.1 Hz, 1H), 7.30–7.16 (m, 6H), 7.15–7.08 (m, 2H), 6.80 (t, *J* = 7.4 Hz, 1H), 5.13 (s, 1H), 3.71 (s, 3H), 3.40–3.34 (m, 1H), 3.24–3.19 (m, 1H), 3.03–2.98 (m, 1H), 2.85–2.81 (m, 1H); ^13^C NMR (151 MHz, CDCl_3_) *δ* 201.5 (C=O), 162.2 (Cq), 158.2 (C=O), 140.1 (Cq), 138.6 (CH), 134.6 (Cq), 132.1 (Cq), 128.9 (CH, 2C), 128.6 (Cq), 128.3 (CH), 126.2 (CH, 2C), 125.7 (CH), 122.4 (CH), 119.7 (CH), 118.9 (CH), 118.1 (CH), 117.6 (Cq), 112.4 (CH), 111.6 (CH), 110.5 (Cq), 71.2 (Cq), 52.5 (CH_3_), 42.2 (CH_2_), 25.3 (CH_2_); HR-ESIMS *m/z* calcd for C_26_H_24_N_3_O_3_ [M + H]^+^ 426.1812, found 426.1815.

***2-Phenyl-2-(2-phenyl-1H-indol-3-yl)indolin-3-one (*6a*)*.** According to procedure D, **6a** was obtained as a yellow solid in 98% yield (19.2 mg; flash chromatographic condition: petroleum ether-acetone 85:15). ^1^H NMR (600 MHz, acetone-*d*_6_) *δ* 10.38 (s, 1H), 7.58–7.53 (m, 2H), 7.51 (ddd, *J* = 8.3, 7.1, 1.3 Hz, 1H), 7.40 (d, *J* = 8.1 Hz, 1H), 7.28 (d, *J* = 7.6 Hz, 1H), 7.22–7.14 (m, 4H), 7.14–7.04 (m, 6H), 7.02 (d, *J* = 8.2 Hz, 1H), 6.81–6.74 (m, 3H); ^13^C NMR (151 MHz, acetone-*d*_6_) *δ* 200.9 (C=O), 160.9 (Cq), 141.7 (Cq), 138.8 (Cq), 138.0 (CH), 137.2 (Cq), 134.6 (Cq), 130.6 (CH, 2C), 128.8 (Cq), 128.6 (CH, 2C), 128.4 (CH), 128.5 (CH, 2C), 128.1 (CH, 2C), 127.9 (CH), 125.4 (CH), 122.3 (CH), 121.9 (CH), 120.8 (Cq), 119.8 (CH), 119.0 (Cq), 118.9 (CH), 113.1 (CH), 111.9 (CH), 72.5 (Cq); HR-ESIMS *m/z* calcd for C_28_H_21_N_2_O [M + H]^+^ 401.1648, found 401.1652.

***2-Methyl-2-(2-methyl-1H-indol-3-yl)indolin-3-one (*6b*)*.** According to procedure D, **6b** was obtained as a yellow solid in 90% yield (12.5 mg; flash chromatographic condition: petroleum ether-acetone 85:15). ^1^H NMR (600 MHz, CDCl_3_) *δ* 7.86 (s, 1H), 7.72–7.69 (m, 1H), 7.51 (d, *J* = 0.9 Hz, 1H), 7.40 (d, *J* = 8.1 Hz, 1H), 7.24–7.22 (m, 1H), 7.08–7.04 (m, 1H), 6.96 (s, 1H), 6.93–6.85 (m, 3H), 2.42 (s, 3H), 1.92 (s, 3H); ^13^C NMR (151 MHz, CDCl_3_) *δ* 204.4 (C=O), 159.7 (Cq), 137.6 (CH), 135.0 (Cq), 132.7 (Cq), 127.6 (Cq), 125.5 (CH), 121.4 (CH), 119.9 (CH), 119.7 (CH), 119.2 (CH), 112.6 (CH), 110.6 (CH), 110.3 (Cq), 109.7 (Cq), 67.3 (Cq), 25.2 (CH_3_), 14.8 (CH_3_); HR-ESIMS *m/z* calcd for C_18_H_17_N_2_O [M + H]^+^ 277.1335, found 277.1336.

***4-Fluoro-2-(4-fluoro-2-phenyl-1H-indol-3-yl)-2-phenylindolin-3-one (*6c*)*.** According to procedure D, **6c** was obtained as a yellow solid in 88% yield (19.2 mg; flash chromatographic condition: petroleum ether-acetone 85:15). ^1^H NMR (600 MHz, DMSO-*d*_6_) *δ* 11.64 (s, 1H), 8.62 (s, 1H), 7.48 (d, *J* = 5.6 Hz, 1H), 7.28–7.23 (m, 2H), 7.20 (d, *J* = 8.1 Hz, 1H), 7.18–7.04 (m, 6H), 6.96 (d, *J* = 2.5 Hz, 3H), 6.83 (d, *J* = 8.3 Hz, 1H), 6.65–6.60 (m, 1H), 6.37 (dd, *J* = 9.5, 8.0 Hz, 1H); ^13^C NMR (151 MHz, DMSO-*d*_6_) *δ* 196.4, 160.9, 160.9, 160.0, 158.3, 155.9, 154.3, 139.4, 139.3, 138.7, 138.6, 138.5, 138.4, 132.7, 129.7, 127.6, 127.4, 127.1, 126.9, 122.2, 122.1, 115.8, 108.2, 107.7, 107.7, 107.6, 107.6, 104.6, 104.5, 102.9, 102.8, 79.2, 71.4; HR-ESIMS *m/z* calcd for C_28_H_19_F_2_N_2_O [M + H]^+^ 437.1460, found 437.1461.

***5-Chloro-2-(5-chloro-2-phenyl-1H-indol-3-yl)-2-phenylindolin-3-one (*6d*)*.** According to procedure D, **6d** was obtained as a yellow solid in 91% yield (21.4 mg; flash chromatographic condition: petroleum ether-acetone 85:15). ^1^H NMR (600 MHz, CDCl_3_) *δ* 8.17 (s, 1H), 7.44–7.40 (m, 2H), 7.37 (dd, *J* = 8.6, 2.2 Hz, 1H), 7.32 (d, *J* = 2.2 Hz, 1H), 7.32–7.28 (m, 1H), 7.25–7.21 (m, 4H), 7.20–7.16 (m, 2H), 7.15–7.12 (m, 2H), 7.10 (dd, *J* = 8.6, 2.0 Hz, 1H), 6.90 (d, *J* = 2.0 Hz, 1H), 6.62 (d, *J* = 8.6 Hz, 1H), 5.12 (s, 1H); ^13^C NMR (151 MHz, CDCl_3_) *δ* 199.1 (C=O), 157.4 (Cq), 139.7 (Cq), 138.5 (Cq), 137.3 (CH), 133.9 (Cq), 132.9 (Cq), 129.8 (CH, 2C), 128.8 (CH), 128.7 (CH, 2C), 128.4 (Cq), 128.2 (CH), 128.0 (CH, 2C), 127.2 (CH, 2C), 125.8 (Cq), 124.7 (CH), 124.5 (Cq), 123.0 (CH), 121.3 (Cq), 121.1 (CH), 113.5 (CH), 111.9 (CH), 111.6 (Cq), 72.9 (Cq); HR-ESIMS *m/z* calcd for C_28_H_19_Cl_2_N_2_O [M + H]^+^ 469.0869, found 469.0869.

***5-Methyl-2-(5-methyl-2-phenyl-1H-indol-3-yl)-2-phenylindolin-3-one (*6e*)*.** According to procedure D, **6e** was obtained as a yellow solid in 96% yield (20.6 mg; flash chromatographic condition: petroleum ether-acetone 85:15). ^1^H NMR (600 MHz, CDCl_3_) *δ* 7.99 (s, 1H), 7.54–7.42 (m, 2H), 7.28 (dd, *J* = 8.3, 1.9 Hz, 1H), 7.25–7.22 (m, 1H), 7.22–7.21 (m, 1H), 7.19 (d, *J* = 8.2 Hz, 1H), 7.18–7.14 (m, 3H), 7.15–7.11 (m, 4H), 6.97 (dd, *J* = 8.3, 1.6 Hz, 1H), 6.82–6.80 (m, 1H), 6.65 (d, *J* = 8.2 Hz, 1H), 5.02 (s, 1H), 2.29 (s, 3H), 2.25(s, 3H); ^13^C NMR (151 MHz, CDCl_3_) *δ* 200.7 (C=O), 158.0 (Cq), 140.8 (Cq), 138.6 (CH), 137.3 (Cq), 134.0 (Cq), 133.6 (Cq), 129.9 (CH, 2C), 129.2 (Cq), 128.9 (Cq), 128.3 (CH, 2C), 128.2 (CH), 127.8 (Cq), 127.7 (CH, 2C), 127.5 (CH), 127.4 (CH, 2C), 124.8 (CH), 124.1 (CH), 121.3 (CH), 120.9 (Cq), 112.5 (CH), 111.9 (Cq), 110.5 (CH), 72.7 (Cq), 21.8(CH_3_), 20.7(CH_3_); HR-ESIMS *m/z* calcd for C_30_H_25_N_2_O [M + H]^+^ 429.1961, found 429.1963.

***5-Methoxy-2-(5-methoxy-2-phenyl-1H-indol-3-yl)-2-phenylindolin-3-one (*6f*)*.** According to procedure D, **6f** was obtained as a yellow solid in 98% yield (22.6 mg; flash chromatographic condition: petroleum ether-acetone 85:15). ^1^H NMR (600 MHz, CDCl_3_) *δ* 8.05 (s, 1H), 7.61–7.49 (m, 2H), 7.25–7.19 (m, 2H), 7.20–7.16 (m, 3H), 7.15–7.12 (m, 2H), 7.12–7.10 (m, 3H), 6.82 (d, *J* = 2.7 Hz, 1H), 6.78 (dd, *J* = 8.8, 2.4 Hz, 1H), 6.72 (dd, *J* = 8.8, 0.5 Hz, 1H), 6.36 (d, *J* = 2.4 Hz, 1H), 4.96 (s, 1H), 3.72 (s, 3H), 3.51 (s, 3H); ^13^C NMR (151 MHz, CDCl_3_) *δ* 200.9 (C=O), 155.2 (Cq), 153.9 (Cq), 153.8 (Cq), 140.6 (Cq), 137.7 (Cq), 133.4 (Cq), 130.7 (Cq), 129.8 (CH. 2C), 128.3 (CH, 2C), 128.3 (CH), 128.0 (Cq), 127.7 (CH), 127.7(CH, 2C), 127.6 (CH), 127.5(CH, 2C), 121.2 (Cq), 114.2 (CH), 112.7 (CH), 112.3 (Cq), 111.5 (CH), 105.3 (CH), 103.3 (CH), 73.1 (Cq), 55.9(CH_3_), 55.5(CH_3_); HR-ESIMS *m/z* calcd for C_30_H_25_N_2_O_3_ [M + H]^+^ 461.1860, found 461.1860.

***6-Methyl-2-(6-methyl-2-phenyl-1H-indol-3-yl)-2-phenylindolin-3-one (*6g*)*.** According to procedure D, **6g** was obtained as a yellow solid in 95% yield (20.3 mg; flash chromatographic condition: petroleum ether-acetone 85:15). ^1^H NMR (600 MHz, acetone-*d*_6_) *δ* 10.19 (s, 1H), 7.51 (d, *J* = 7.7 Hz, 2H), 7.20–7.16 (m, 4H), 7.14 (s, 1H), 7.09–7.04 (m, 6H), 6.82 (s, 1H), 6.67 (d, *J* = 8.3 Hz, 1H), 6.61 (d, *J* = 8.2 Hz, 2H), 2.37 (s, 3H), 2.35 (s, 3H); ^13^C NMR (151 MHz, acetone-*d*_6_) *δ* 200.1 (C=O), 161.3 (Cq), 149.2 (Cq), 142.0 (Cq), 138.1 (Cq), 137.6 (Cq), 134.8 (Cq), 131.7 (CH), 130.6 (CH, 2C), 128.4 (CH, 2C), 128.2 (CH, 2C), 128.1 (CH), 128.0 (CH, 2C), 127.7 (CH), 126.8 (Cq), 125.1 (CH), 121.7 (CH), 121.5 (CH), 120.6 (Cq), 120.6 (Cq), 118.7 (Cq), 112.9 (CH), 111.7 (CH), 72.7 (Cq), 22.5(CH_3_), 21.6(CH_3_); HR-ESIMS *m/z* calcd for C_30_H_25_N_2_O [M + H]^+^ 429.1961, found 429.1962.

***7-Methyl-2-(7-methyl-2-phenyl-1H-indol-3-yl)-2-phenylindolin-3-one (*6h*)*.** According to procedure D, **6h** was obtained as a yellow solid in 92% yield (19.8 mg; flash chromatographic condition: petroleum ether-acetone 85:15). ^1^H NMR (600 MHz, CDCl_3_) *δ* 7.97 (s, 1H), 7.57–7.43 (m, 2H), 7.33–7.28 (m, 2H), 7.24 (dt, *J* = 7.1, 1.1 Hz, 1H), 7.23–7.19 (m, 3H), 7.19–7.15 (m, 3H), 6.99–6.91 (m, 2H), 6.87 (dd, *J* = 8.2, 7.1 Hz, 1H), 6.74 (t, *J* = 7.4 Hz, 1H), 4.89 (s, 1H), 2.45 (s, 3H), 1.94 (s, 3H); ^13^C NMR (151 MHz, CDCl_3_) *δ* 200.9 (C=O), 158.5 (Cq), 141.0 (Cq), 137.1 (CH), 136.7 (Cq), 135.3 (Cq), 134.0 (Cq), 133.6 (Cq), 129.8 (CH, 2C), 129.2 (Cq), 128.4 (CH, 2C), 128.0 (CH, 2C), 127.7 (CH), 127.5 (CH, 2C), 127.0 (Cq), 123.1 (CH), 122.8 (CH), 121.4 (CH), 120.4 (CH), 119.9 (CH), 119.9 (Cq), 119.4 (CH), 112.6 (Cq), 72.4 (Cq), 16.7 (CH_3_), 15.6 (CH_3_); HR-ESIMS *m/z* calcd for C_30_H_25_N_2_O [M + H]^+^ 429.1961, found 429.1965.

***[3,2′:2′,3″-Terindolin]-3′-one (*7a*)*.** According to procedure D, **7a** was obtained as a yellow solid in 75% yield (18.1 mg; flash chromatographic condition: petroleum ether-acetone 80:20). ^1^H NMR (600 MHz, acetone-*d*_6_) *δ* 10.16 (s, 2H), 7.56 (d, *J* = 7.7 Hz, 1H), 7.53–7.49 (m, 1H), 7.46 (d, *J* = 8.1 Hz, 2H), 7.38 (d, *J* = 8.2 Hz, 2H), 7.26–7.22 (m, 2H), 7.15 (s, 1H), 7.07–7.01 (m, 3H), 6.84 (ddd, *J* = 8.1, 7.0, 1.1 Hz, 2H), 6.82–6.77 (m, 1H); ^13^C NMR (151 MHz, acetone-*d*_6_) *δ* 201.4 (C=O), 161.6 (Cq), 138.4 (Cq, 2C), 138.0 (CH), 127.1 (CH), 125.4 (Cq, 2C), 125.0 (CH, 2C), 122.2 (CH, 2C), 121.8 (CH, 2C), 120.1 (Cq), 119.5 (CH, 2C), 118.6 (CH), 116.0 (CH), 113.1 (Cq, 2C), 112.3 (CH, 2C), 69.0 (Cq); HR-ESIMS *m/z* calcd for C_24_H_18_N_3_O [M + H]^+^ 364.1444, found 364.1445.

***4,4′,4″-Trifluoro-[3,2′:2′,3″-terindolin]-3′-one (*7b*)*.** According to procedure D, **7b** was obtained as a yellow solid in 74% yield (20.5 mg; flash chromatographic condition: petroleum ether-acetone 80:20). ^1^H NMR (600 MHz, DMSO-*d*_6_) *δ* 11.29 (s, 2H), 7.69 (s, 1H), 7.43–7.37 (m, 1H), 7.24 (d, *J* = 8.1 Hz, 2H), 7.10–7.05 (m, 2H), 6.90 (s, 2H), 6.76 (d, *J* = 8.2 Hz, 1H), 6.71–6.66 (m, 2H), 6.40–6.36 (m, 1H); ^13^C NMR (151 MHz, DMSO-*d*_6_) *δ* 196.8, 161.6, 161.5, 160.0, 158.3, 156.2, 154.5, 140.0, 140.0, 138.4, 138.4, 125.6, 122.1, 122.0, 114.2, 114.1, 112.1, 112.1, 108.7, 108.7, 108.3, 108.3, 107.0, 106.9, 104.3, 104.2, 102.3, 102.2, 67.2; HR-ESIMS *m/z* calcd for C_24_H_15_F_3_N_3_O [M + H]^+^ 418.1162, found 418.1162.

***5,5′,5″-Trifluoro-[3,2′:2′,3″-terindolin]-3′-one (*7c*)*.** According to procedure D, **7c** was obtained as a yellow solid in 73% yield (20.3 mg; flash chromatographic condition: petroleum ether-acetone 80:20).^1^H NMR (600 MHz, CDCl_3_) *δ* 8.08 (s, 2H), 7.38 (dd, *J* = 7.2, 2.7 Hz, 1H), 7.34–7.26 (m, 2H), 7.25 (s, 1H), 7.18 (s, 2H), 7.06–7.02 (m, 2H), 6.93–6.87 (m, 3H), 5.25 (s, 1H); ^13^C NMR (151 MHz, CDCl_3_) *δ* 200.7, 200.7, 158.5, 157.8, 156.9, 156.8, 156.2, 133.5, 126.0, 125.9, 125.8, 125.5, 120.5, 120.4, 114.9, 114.8, 114.4, 114.4, 112.3, 112.3, 111.1, 110.9, 110.3, 110.2, 105.4, 105.3, 69.0; HR-ESIMS *m/z* calcd for C_24_H_15_F_3_N_3_O [M + H]^+^ 418.1162, found 418.1163.

***5,5′,5″-Trimethyl-[3,2′:2′,3″-terindolin]-3′-one (*7d*)*.** According to procedure D, **7d** was obtained as a yellow solid in 72% yield (19.4 mg; flash chromatographic condition: petroleum ether-acetone 80:20). ^1^H NMR (600 MHz, acetone-*d*_6_) *δ* 10.00 (s, 2H), 7.38–7.34 (m, 2H), 7.28–7.24 (m, 4H), 7.16 (d, *J* = 2.6 Hz, 2H), 6.96 (dd, *J* = 8.9, 1.7 Hz, 1H), 6.90–6.84 (m, 3H), 2.29 (s, 3H), 2.22 (s, 6H); ^13^C NMR (151 MHz, acetone-*d*_6_) *δ* 201.6 (C=O), 160.3 (Cq), 139.3 (CH), 136.9 (Cq, 2C), 128.1 (Cq, 2C), 127.5 (Cq, 2C), 125.1 (CH, 2C), 125.0 (Cq), 124.8 (CH), 123.9 (CH, 2C), 121.6 (CH, 2C), 120.6 (Cq), 115.8 (Cq, 2C), 113.3 (CH), 112.1 (CH, 2C), 69.6 (Cq), 21.9 (CH_3_, 2C), 20.7 (CH_3_); HR-ESIMS *m/z* calcd for C_27_H_24_N_3_O [M + H]^+^ 406.1914, found 406.1915.

***5,5′,5″-Trimethoxy-[3,2′:2′,3″-terindolin]-3′-one (*7e*)*.** According to procedure D, **7e** was obtained as a yellow solid in 66% yield (19.9 mg; flash chromatographic condition: petroleum ether-acetone 80:20). ^1^H NMR (600 MHz, DMSO-*d*_6_) *δ* 10.79 (s, 2H), 7.79 (s, 1H), 7.27–7.22 (m, 3H), 7.05 (d, *J* = 2.5 Hz, 2H), 6.99 (s, 1H), 6.95 (d, *J* = 8.8 Hz, 1H), 6.82 (d, *J* = 2.5 Hz, 2H), 6.72–6.70 (m, 2H), 3.73 (s, 3H), 3.54 (s, 6H). ^13^C NMR (151 MHz, DMSO-*d*_6_) *δ* 201.2 (C=O), 156.9 (Cq), 152.6 (Cq, 2C), 151.8 (Cq), 132.1 (Cq, 2C), 127.8 (Cq), 126.1 (Cq, 2C), 124.7 (CH, 2C), 118.0 (CH), 113.7 (Cq, 2C), 113.5 (CH), 112.1 (CH, 2C), 110.6 (CH, 2C), 104.6 (CH), 103.1 (CH, 2C), 68.5 (Cq), 55.6 (CH_3_), 55.1 (CH_3_, 2C); HR-ESIMS *m/z* calcd for C_27_H_24_N_3_O_4_ [M + H]^+^ 454.1761, found 454.1761.

***6,6′,6″-Trimethyl-[3,2′:2′,3″-terindolin]-3′-one (*7f*)*.** According to procedure D, **7f** was obtained as a yellow solid in 64% yield (17.3 mg; flash chromatographic condition: petroleum ether-acetone 80:20). ^1^H NMR (600 MHz, acetone-*d*_6_) *δ* 9.97 (s, 2H), 7.43 (d, *J* = 7.9 Hz, 1H), 7.32 (d, *J* = 8.1 Hz, 2H), 7.16 (s, 2H), 7.15–7.12 (m, 2H), 6.95 (s, 1H), 6.68 (dd, *J* = 8.2, 1.5 Hz, 2H), 6.63 (dt, *J* = 7.9, 1.5 Hz, 1H), 2.35 (s, 3H), 2.34 (s, 6H); ^13^C NMR (151 MHz, acetone-*d*_6_) *δ* 199.7 (C=O), 161.1 (Cq), 148.1 (Cq), 137.9 (Cq, 2C), 130.6 (Cq, 2C), 124.3 (CH, 2C), 124.2 (CH), 123.3 (Cq, 2C), 120.7 (CH, 2C), 120.3 (CH, 2C), 119.4 (CH), 117.2 (Cq), 115.3 (CH, 2C), 112.1 (CH), 111.2 (Cq, 2C), 68.4 (Cq), 21.6 (CH_3_), 20.8 (CH_3_, 2C); HR-ESIMS *m/z* calcd for C_27_H_24_N_3_O [M + H]^+^406.1914, found 406.1916.

***7,7′,7″-Trimethyl-[3,2′:2′,3″-terindolin]-3′-one (*7g*)*.** According to procedure D, **7g** was obtained as a yellow solid in 65% yield (17.6 mg; flash chromatographic condition: petroleum ether-acetone 80:20). ^1^H NMR (600 MHz, acetone-*d*_6_) *δ* 8.01 (s, 2H), 7.63–7.58 (m, 1H), 7.35 (dt, *J* = 7.1, 1.2 Hz, 1H), 7.27 (s, 1H), 7.08 (d, *J* = 2.3 Hz, 2H), 6.97 (dt, *J* = 7.1, 1.1 Hz, 2H), 6.91 (dd, *J* = 8.0, 7.1 Hz, 2H), 6.85 (t, *J* = 7.5 Hz, 1H), 5.28 (s, 1H), 2.45 (s, 6H), 2.22 (s, 3H); ^13^C NMR (151 MHz, acetone-*d*_6_) *δ* 201.7 (C=O), 159.7 (Cq), 137.6 (CH), 136.7 (CH), 125.4 (Cq, 2C), 124.1 (Cq, 2C), 122.9 (CH), 122.8 (CH, 2C), 122.1 (Cq), 120.8 (Cq, 2C), 120.2 (CH, 2C), 119.8 (Cq), 119.6 (CH, 2C), 118.2 (CH, 2C), 115.8 (Cq, 2C), 68.6 (Cq), 16.7 (CH_3_, 2C), 15.9 (CH_3_); HR-ESIMS *m/z* calcd for C_27_H_24_N_3_O [M + H]^+^ 406.1914, found 406.1911.

***2-Phenyl-2-(1H-pyrrol-2-yl)indolin-3-one (*8*)*.** According to procedure B, **8** was obtained as a yellow solid in 94% yield (25.9 mg; flash chromatographic condition: petroleum ether-acetone 80:20). ^1^H NMR (600 MHz, CDCl_3_) *δ 8*.84 (s, 1H), 7.65 (d, *J* = 7.8 Hz, 1H), 7.51 (ddd, *J* = 8.4, 7.1, 1.4 Hz, 1H), 7.32–7.21 (m, 5H), 6.94 (d, *J* = 8.3 Hz, 1H), 6.91–6.86 (m, 1H), 6.79 (td, *J* = 2.7, 1.5 Hz, 1H), 6.27 (ddd, *J* = 3.9, 2.6, 1.5 Hz, 1H), 6.21 (dt, *J* = 3.5, 2.7 Hz, 1H), 5.38 (s, 1H); ^13^C NMR (151 MHz, CDCl_3_) *δ* 201.2 (C=O), 161.0 (Cq), 140.8 (Cq), 138.0 (CH), 129.1 (Cq), 128.9 (CH, 2C), 128.4 (CH), 126.8 (CH, 2C), 125.7 (CH), 119.9 (CH), 119.6 (Cq), 118.7 (CH), 112.8 (CH), 108.5 (CH), 107.2 (CH), 71.0 (Cq); HR-ESIMS *m/z* calcd for C_18_H_15_N_2_O [M + H]^+^ 275.1179, found 275.1177. 

***2-(1-Methyl-1H-pyrrol-3-yl)-2-phenylindolin-3-one(*9*)*.** According to procedure B, **9** was obtained as a yellow solid in 90% yield (25.9 mg; flash chromatographic condition: petroleum ether-acetone 85:15). ^1^H NMR (600 MHz, CDCl_3_) *δ* 7.62 (d, *J* = 7.6 Hz, 1H), 7.52 (dd, *J* = 5.3, 3.4 Hz, 2H), 7.45 (ddd, *J* = 8.3, 7.1, 1.3 Hz, 1H), 7.31–7.26 (m, 2H), 7.24 (ddd, *J* = 7.2, 4.3, 1.3 Hz, 1H), 6.90 (d, *J* = 8.2 Hz, 1H), 6.84–6.80 (m, 1H), 6.56 (dt, *J* = 5.0, 2.2 Hz, 2H), 5.98 (dd, *J* = 2.6, 1.9 Hz, 1H), 5.15 (s, 1H), 3.56 (s, 3H). ^13^C NMR (151 MHz, CDCl_3_) *δ* 201.3 (C=O), 160.4 (Cq), 140.9 (Cq), 137.4 (CH), 128.3 (CH, 2C), 127.6 (CH), 126.9 (CH, 2C), 125.6 (CH), 123.9 (Cq), 122.6 (CH), 120.8 (CH), 119.7 (Cq), 119.4 (CH), 112.7 (CH), 107.3 (CH), 71.3 (CH), 36.3 (CH_3_); HR-ESIMS *m/z* calcd for C_19_H_17_N_2_O [M + H]^+^ 289.1335, found 289.1333.

***2-Phenyl-2-(thiophen-2-yl)indolin-3-one (*10*)*.** According to procedure B, **10** was obtained as a yellow solid in 75% yield (21.8 mg; flash chromatographic condition: petroleum ether-acetone 80:20). ^1^H NMR (600 MHz, CDCl_3_) *δ* 7.67 (d, *J* = 7.7 Hz, 1H), 7.56–7.45 (m, 3H), 7.39–7.29 (m, 3H), 7.25 (d, *J* = 5.2 Hz, 1H), 7.12 (dd, *J* = 3.7, 1.2 Hz, 1H), 7.00 (dd, *J* = 5.1, 3.6 Hz, 1H), 6.96 (d, *J* = 8.2 Hz, 1H), 6.91 (t, *J* = 7.4 Hz, 1H), 5.35 (s, 1H); ^13^C NMR (151 MHz, CDCl_3_) *δ* 199.4 (C=O), 160.0 (Cq), 144.6 (Cq), 140.5 (Cq), 137.9 (CH), 128.7 (CH, 2C), 128.4 (CH), 127.3 (CH), 126.9 (CH, 2C), 126.4 (CH), 125.9 (CH), 125.4 (CH), 120.2 (CH), 119.4 (Cq), 112.8 (CH), 72.4 (Cq); HR-ESIMS *m/z* calcd for C_18_H_14_NOS [M + H]^+^ 292.0791, found 292.0791.

***2-(3-Oxo-2-phenylindolin-2-yl)acetaldehyde (*11*)*.** According to procedure B, **11** was obtained as a yellow solid in 72% yield (18.1 mg; flash chromatographic condition: petroleum ether-acetone 90:10). ^1^H NMR (600 MHz, CDCl_3_) *δ* 9.70 (d, *J* = 1.7 Hz, 1H), 7.59 (d, *J* = 7.7 Hz, 1H), 7.53–7.49 (m, 3H), 7.36–7.32 (m, 2H), 7.30–7.28 (m, 1H), 6.97 (d, *J* = 8.3 Hz, 1H), 6.86 (t, *J* = 7.4 Hz, 1H), 5.70 (s, 1H), 3.64 (dd, *J* = 17.6, 1.9 Hz, 1H), 2.98 (d, *J* = 17.5 Hz, 1H); ^13^C NMR (151 MHz, CDCl_3_) *δ* 199.9 (C=O), 199.8 (C=O), 160.2 (Cq) 138.0 (CH), 137.7 (Cq), 129.1 (CH, 2C), 128.1 (CH), 125.8 (CH), 125.4 (CH, 2C), 119.6 (CH), 118.5 (Cq), 112.1 (CH), 68.7 (Cq), 50.4 (CH_2_); HR-ESIMS *m/z* calcd for C_16_H_14_NO_2_ [M + H]^+^ 252.1019, found 252.1021.

***2-(2-Oxopropyl)-2-phenylindolin-3-one (*12*)*.** According to procedure B using 5 equiv of MsOH as additive, **12** was obtained as a yellow solid in 70% yield (18.5 mg; flash chromatographic condition: petroleum ether-acetone 90:10). ^1^H NMR (600 MHz, CDCl_3_) *δ* 7.57–7.52 (m, 3H), 7.48 (ddd, *J* = 8.3, 7.0, 1.3 Hz, 1H), 7.34–7.30 (m, 2H), 7.28–7.22 (m, 1H), 6.95 (d, *J* = 8.3 Hz, 1H), 6.80 (t, *J* = 7.4 Hz, 1H), 6.13 (s, 1H), 3.73 (d, *J* = 17.4 Hz, 1H), 2.73 (d, *J* = 17.4 Hz, 1H), 2.10 (s, 3H). ^13^C NMR (151 MHz, CDCl_3_) *δ* 206.9 (C=O), 200.4 (C=O), 160.2 (Cq), 137.9 (CH), 137.8 (Cq), 128.8 (CH, 2C), 127.8 (CH), 125.6 (CH), 125.5 (CH, 2C), 119.1 (CH), 118.3 (Cq), 112.0 (CH), 69.1 (Cq), 49.6 (CH_2_), 31.6 (CH_3_); HR-ESIMS *m/z* calcd for C_17_H_16_NO_2_ [M + H]^+^ 265.1103, found 2665.1101.

## 4. Conclusions

In summary, an oxidative cross-dehydrogenative coupling of indoles with 1,3-dicarbonyl compounds and indoles has been developed. The reaction proceeds smoothly under mild conditions and features a broad substrate scope with excellent functional group tolerance, affording structurally diverse 2,2-disubstituted indolin-3-ones in high yields. Oxidative dimerization or trimerization of indoles was achieved under the same conditions. Moreover, a variety of C-H nucleophiles such as pyrrole, thiophene, acetaldehyde, and acetone were also suitable substrates and all the 2,2-disubstituted indolin-3-ones were obtained as racemic molecules.

## Figures and Tables

**Table 1:**
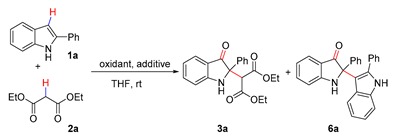
Optimization of reaction conditions ^[a]^.

Entry	Oxidant	additive	Yield (%) ^[b]^	
			**3a**	**6a**
1	TEMPO^+^ClO_4_^−^	-	0	96
2	TEMPO^+^ClO_4_^−^	CuCl	79	7
3	TEMPO^+^ClO_4_^−^	CuCl_2_	86	<5
4	TEMPO^+^ClO_4_^−^	Cu(OTf)_2_	95	-
5	TEMPO^+^ClO_4_^−^	Zn(OTf)_2_	92	-
6	TEMPO^+^ClO_4_^−^	Yb(OTf)_2_	40	<5
7	TEMPO^+^OTf^−^	Cu(OTf)_2_	93	-
8	TEMPO^+^BF_4_^−^	Cu(OTf)_2_	98	-
9	TEMPO^+^PF_6_^−^	Cu(OTf)_2_	90	-
10 ^[c]^	TEMPO^+^BF_4_^−^	Cu(OTf)_2_	98	-
11 ^[d]^	TEMPO^+^BF_4_^−^	-	-	98

^[a]^ Reaction conditions: **1a** (0.1 mmol), **2a** (0.2 mmol), additive (0.05 eq.) and oxidant (0.1 mmol) in THF (1.0 mL) at room temperature. ^[b]^ Yield of isolated product. ^[c]^ 0.005 eq. Cu(OTf)_2_ was added. ^[d]^ The reaction was performed without extra nucleophile.

## Supplementary Materials

The Supplementary Materials are available online.
