# Supplementary material for: Oxidative Dearomative Cross-Dehydrogenative Coupling of Indoles with Diverse C-H Nucleophiles: Efficient Approach to 2,2-Disubstituted Indolin-3-ones
Source: Molecules. 2020 Jan 20;25(2):419. doi: 10.3390/molecules25020419 (PMC7024378; doi:10.3390/molecules25020419)

Oxidative Dearomative Cross-Dehydrogenative Coupling of Indoles with Diverse C-H Nucleophiles: Efficient Approach to 2,2-Disubstituted Indolin-3-ones

Xue Yan,[1,2] Ying-De Tang,[1,2] Cheng-Shi Jiang,[2] Xigong Liu[2,3,*], and Hua Zhang[2,*]

[1] School of Chemistry and Chemical Engineering, University of Jinan, Jinan 250022, China

[2] School of Biological Science and Technology, University of Jinan, Jinan 250022, China

E-mail：bio_zhangh@ujn.edu.cn

[3] School of Chemistry and Chemical Engineering, Shandong University, Jinan 250100, China

E-mail: 201990000024@sdu.edu.cn

^{1}H and ^{13}C NMR spectra of synthesized compounds

^{1}H NMR spectrum of compound 3a in $CDCl_3$ (600 MHz)

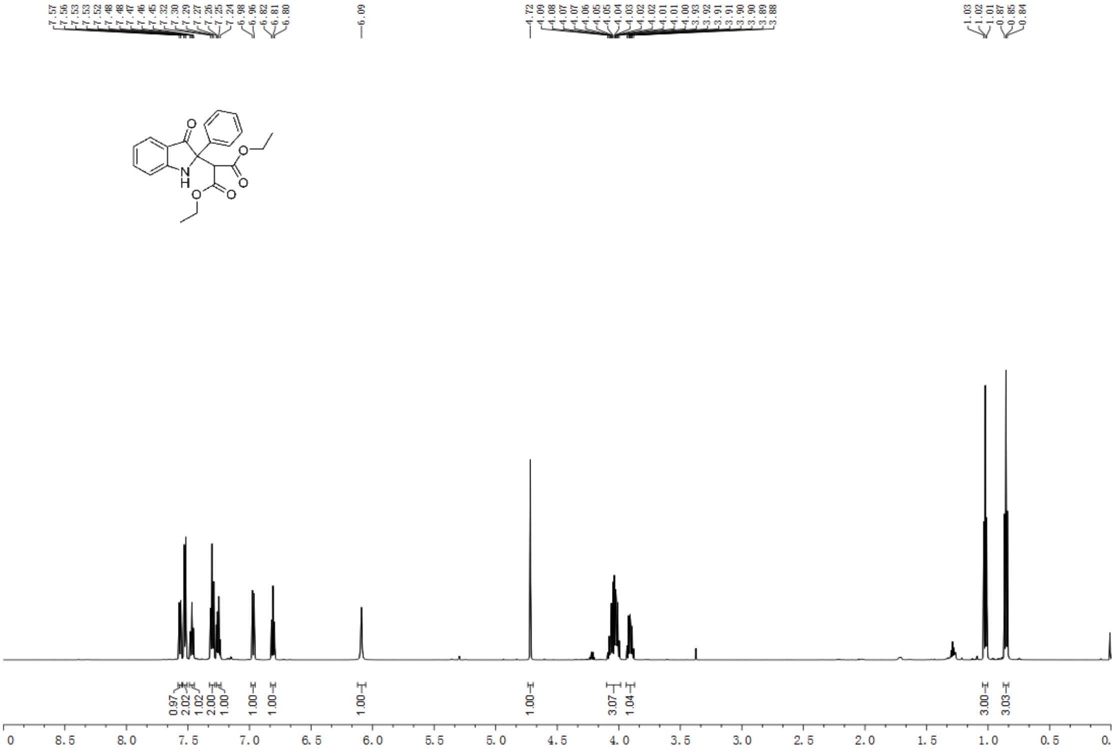

^{13}C NMR spectrum of compound 3a in $CDCl_3$ (151 MHz)

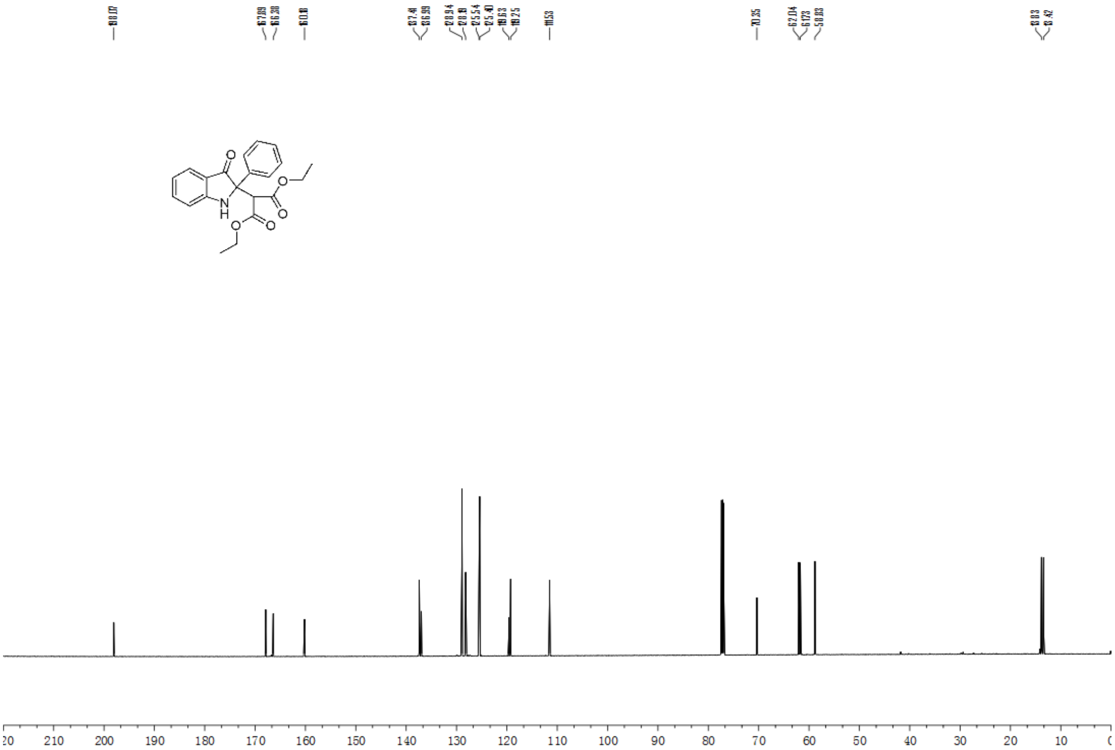

^{1}H NMR spectrum of compound 3b in $CDCl_3$ (600 MHz)

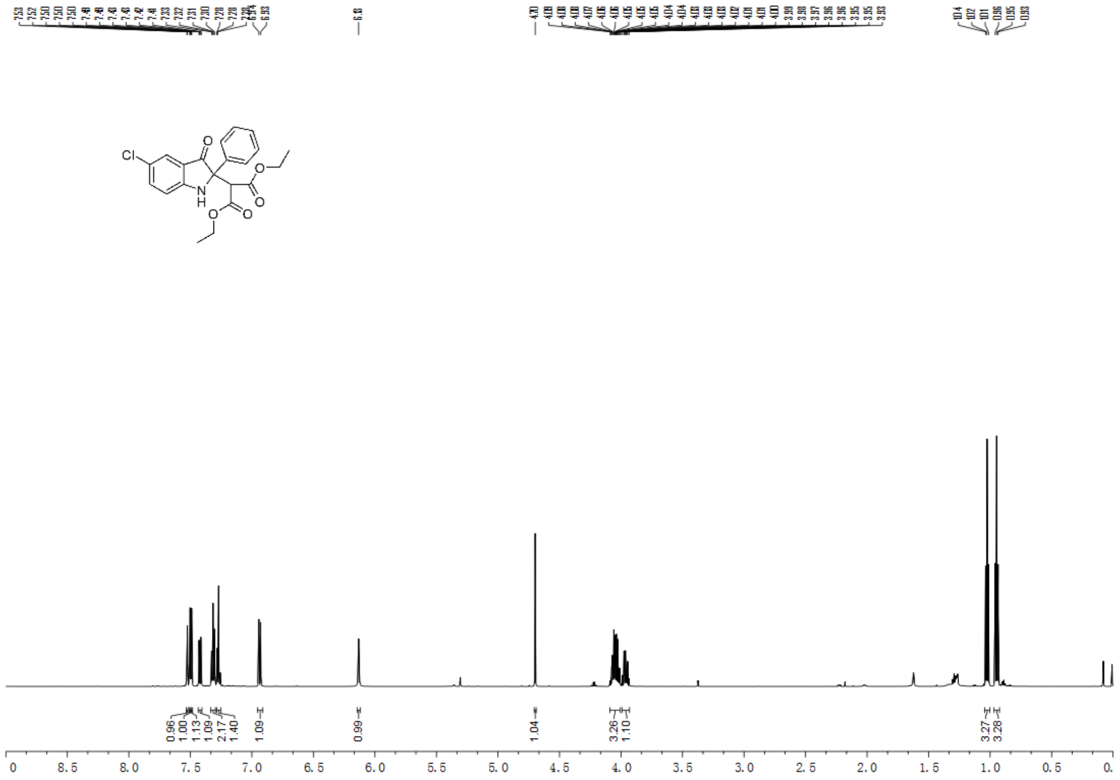

^{13}C NMR spectrum of compound 3b in $CDCl_3$ (151 MHz)

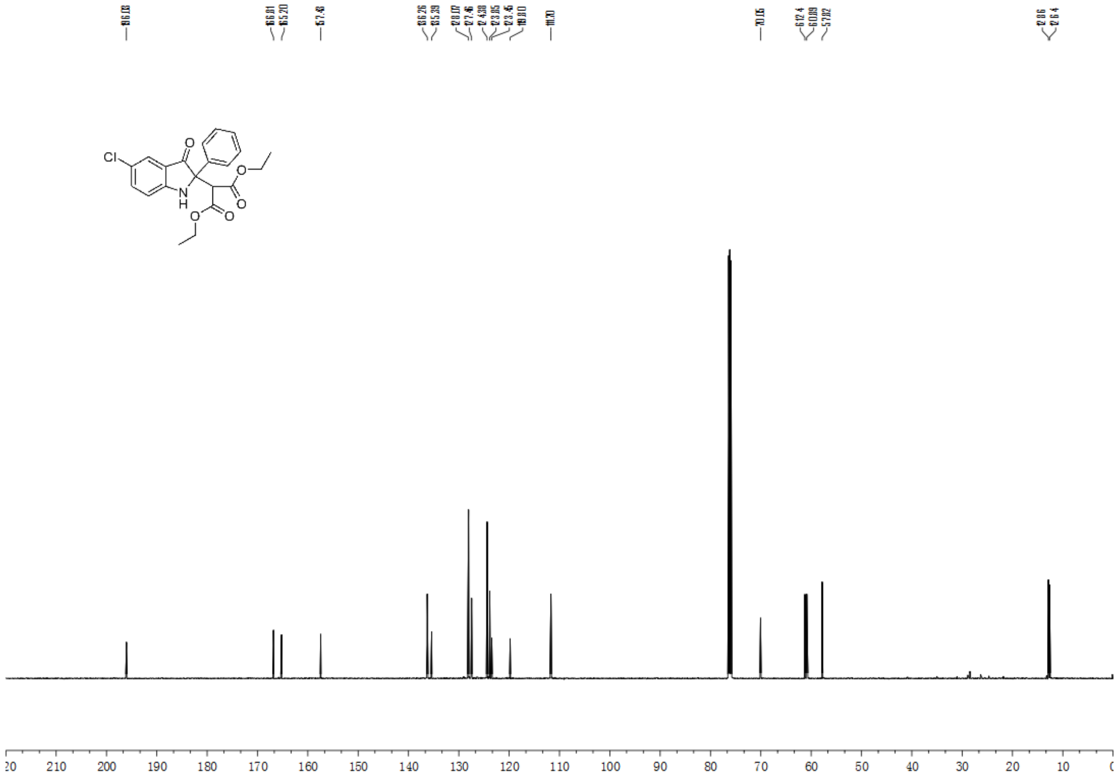

^{1}H NMR spectrum of compound 3c in $CDCl_3$ (600 MHz)

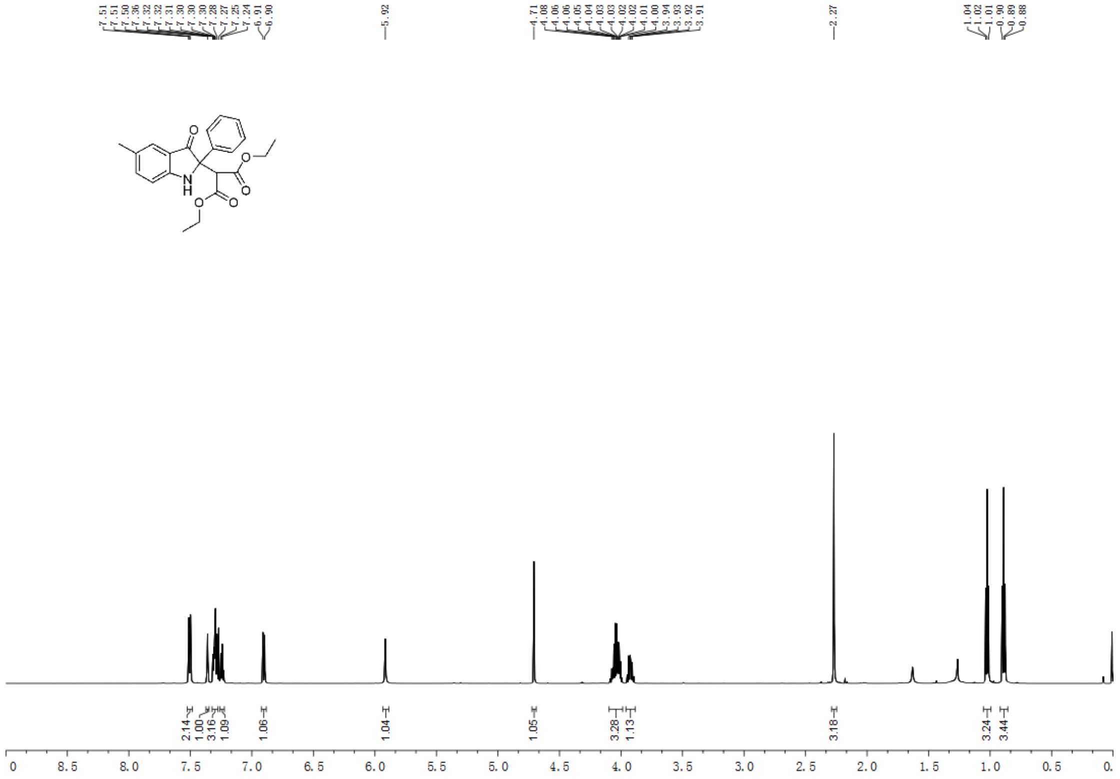

^{13}C NMR spectrum of compound 3c in $CDCl_3$ (151 MHz)

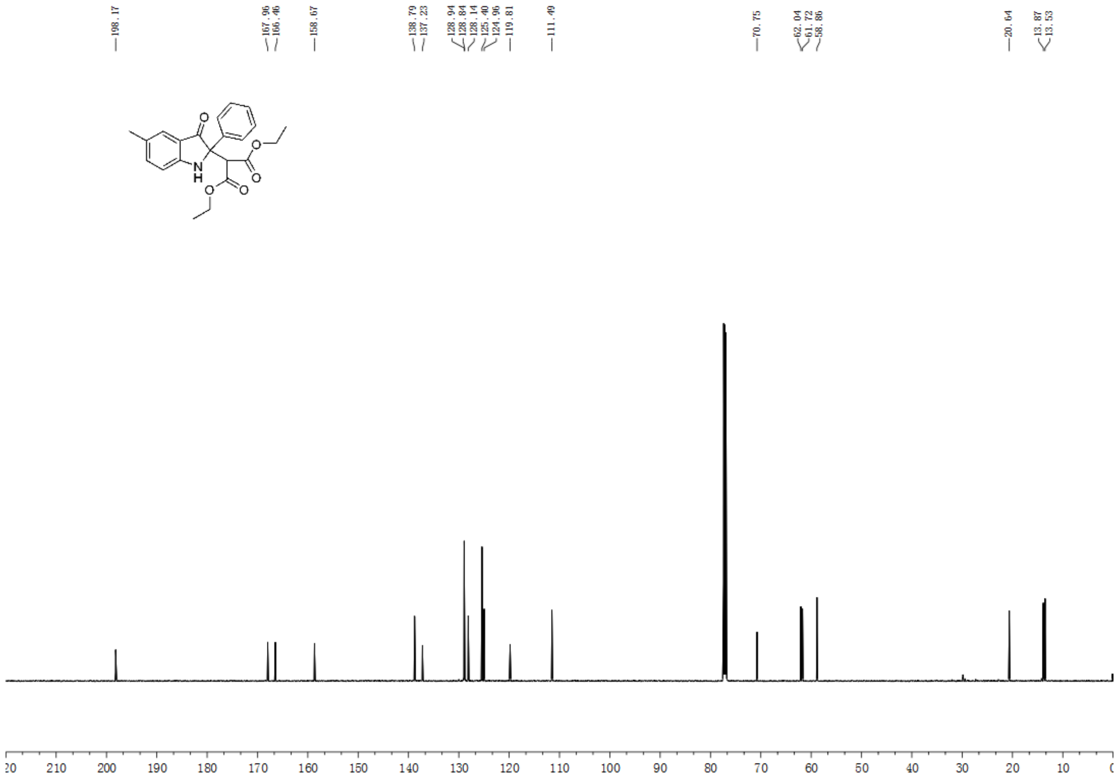

^{1}H NMR spectrum of compound 3d in $CDCl_3$ (600 MHz)

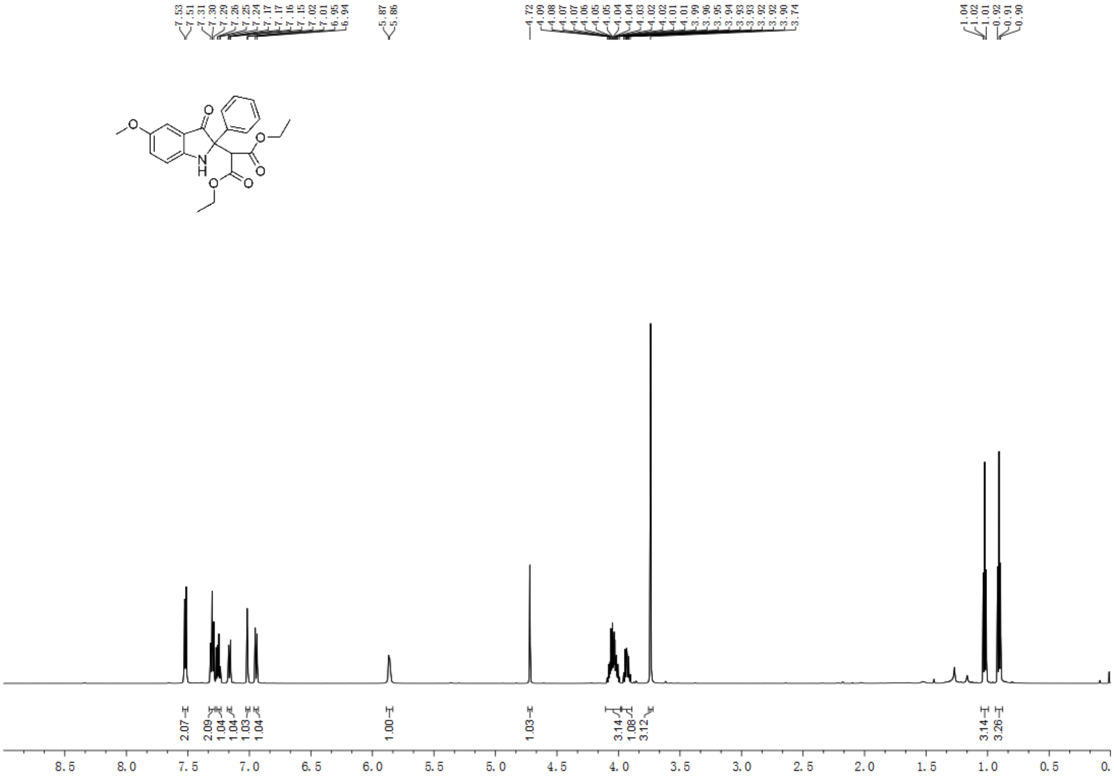

^{13}C NMR spectrum of compound 3d in $CDCl_3$ (151MHz)

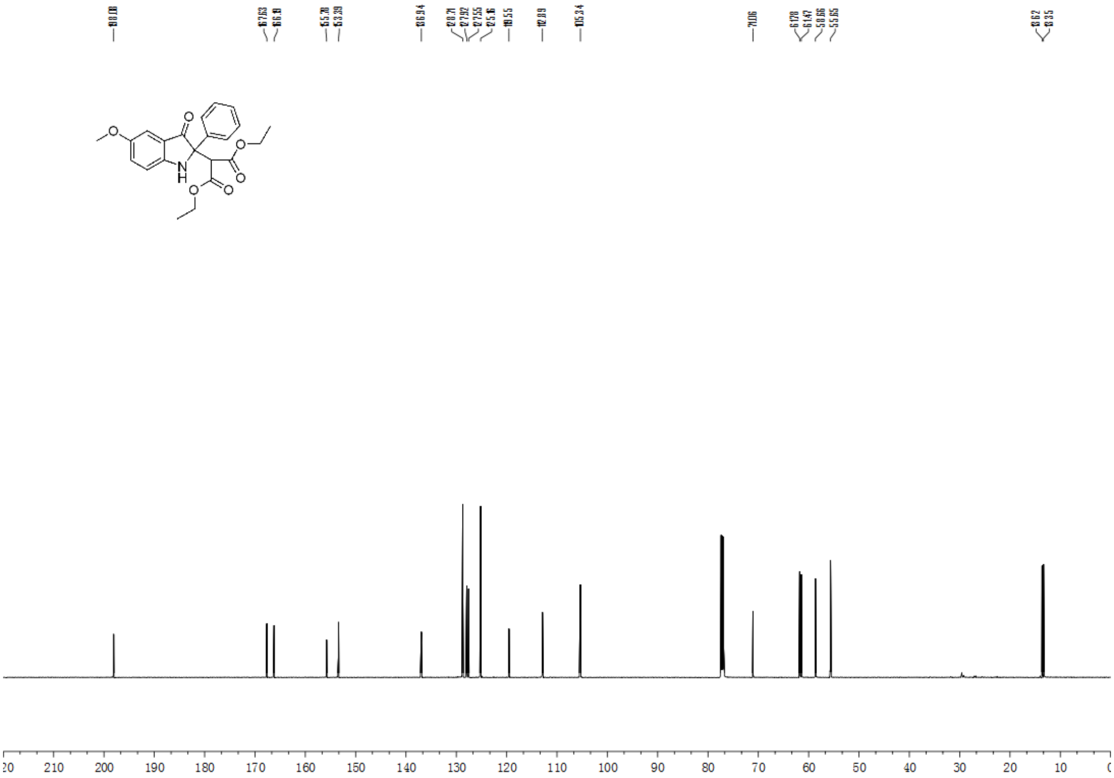

^{1}H NMR spectrum of compound 3e in $CDCl_3$ (600 MHz)

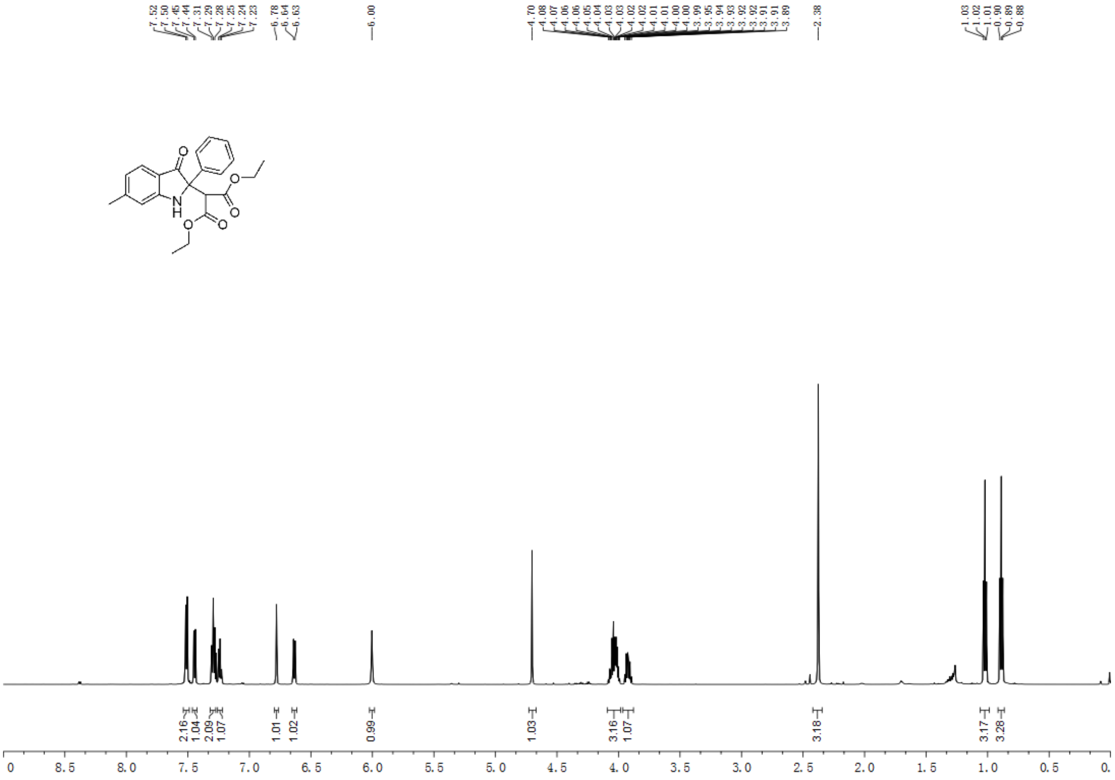

^{13}C NMR spectrum of compound 3e in $CDCl_3$ (151 MHz)

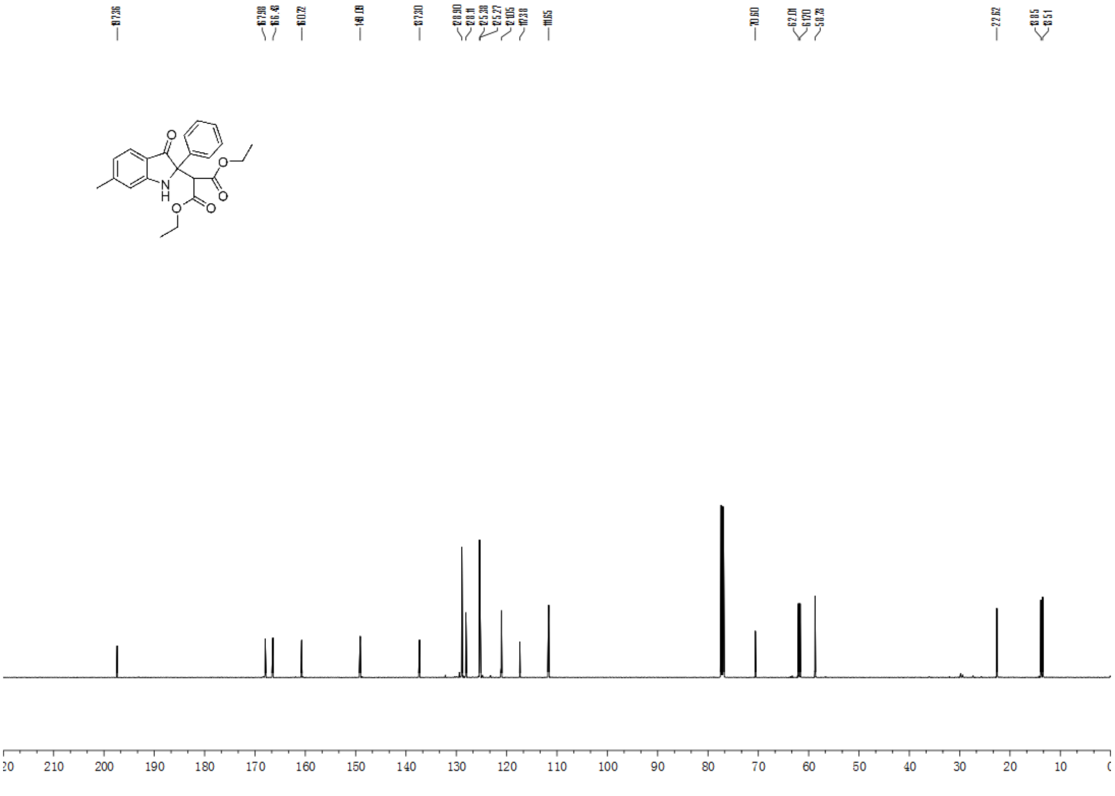

^{1}H NMR spectrum of compound 3f in $CDCl_3$ (600 MHz)

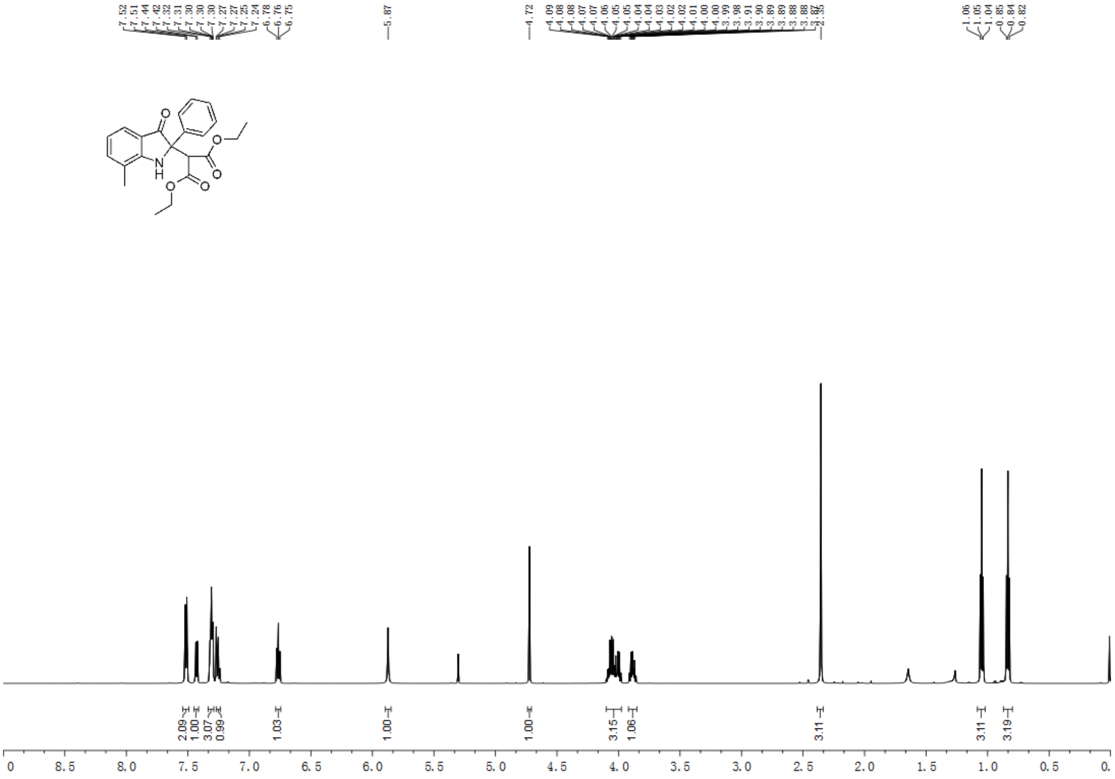

^{13}C NMR spectrum of compound 3f in $CDCl_3$ (151 MHz)

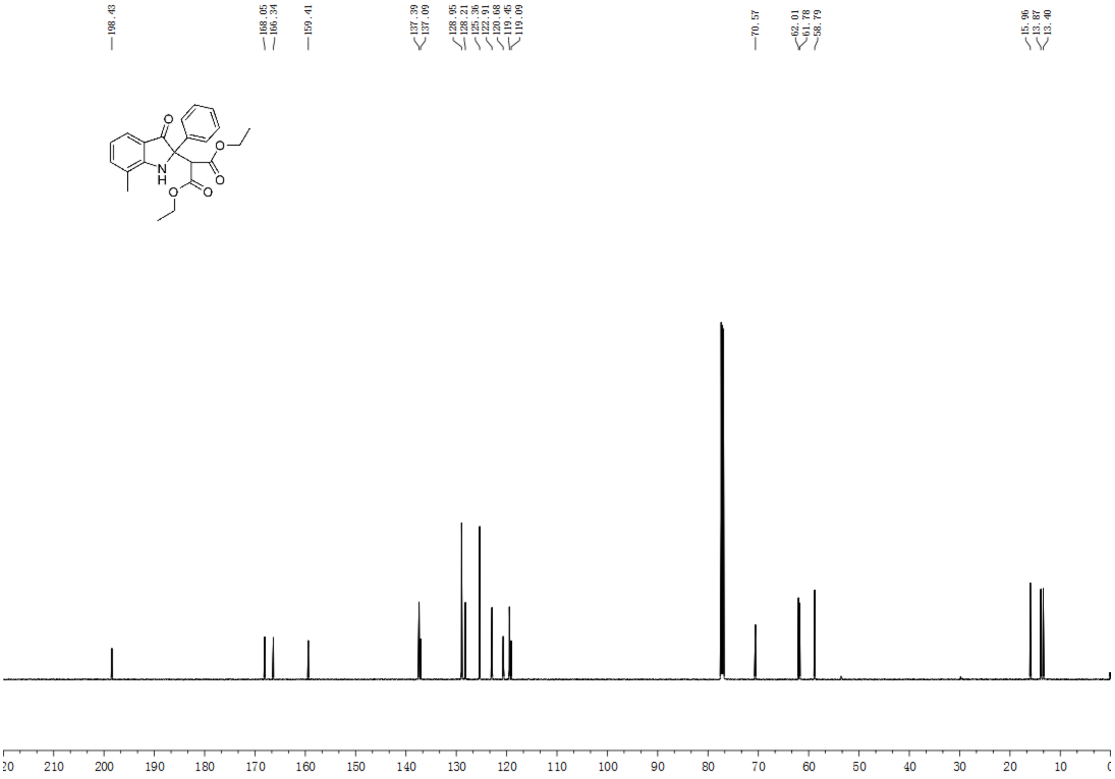

^{1}H NMR spectrum of compound 3g in $CDCl_3$ (600 MHz)

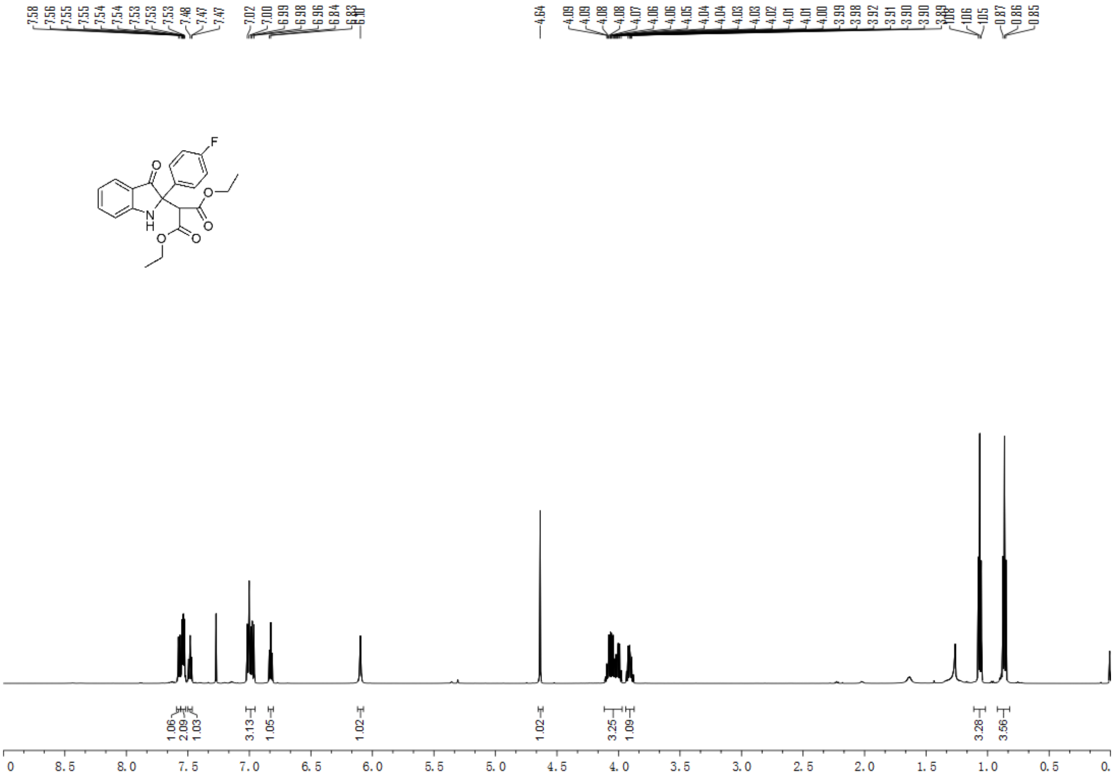

^{13}C NMR spectrum of compound 3g in $CDCl_3$ (151 MHz)

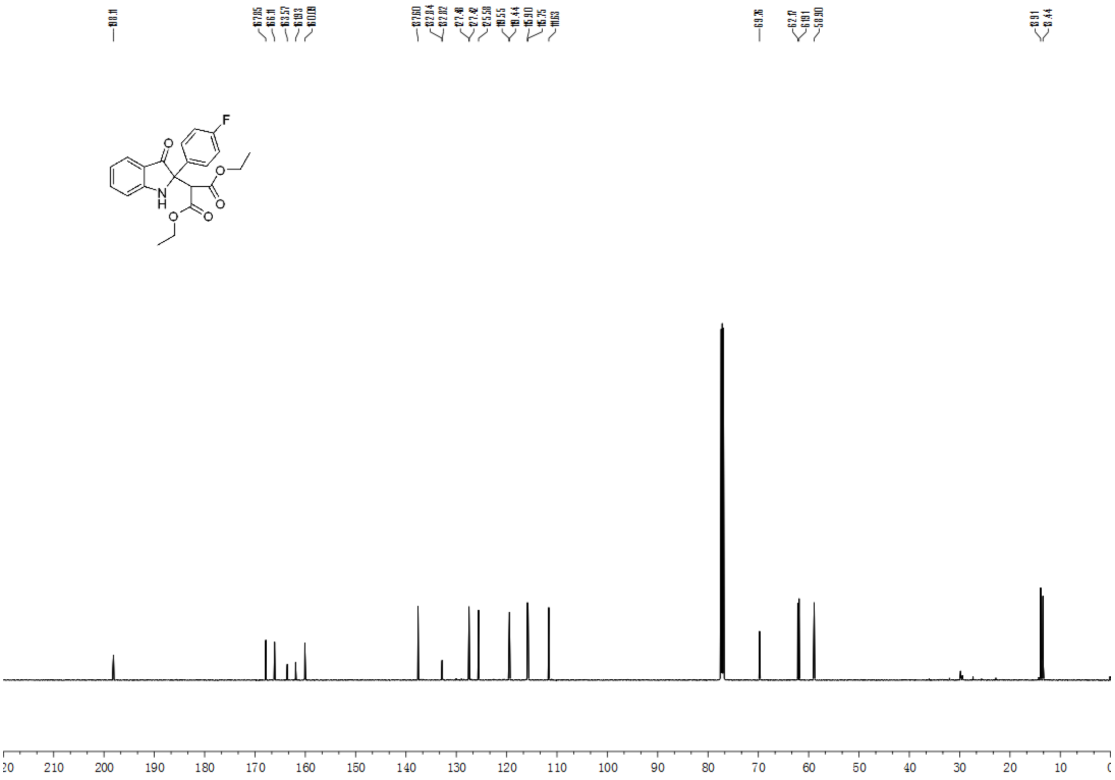

^{1}H NMR spectrum of compound 3h in $CDCl_3$ (600 MHz)

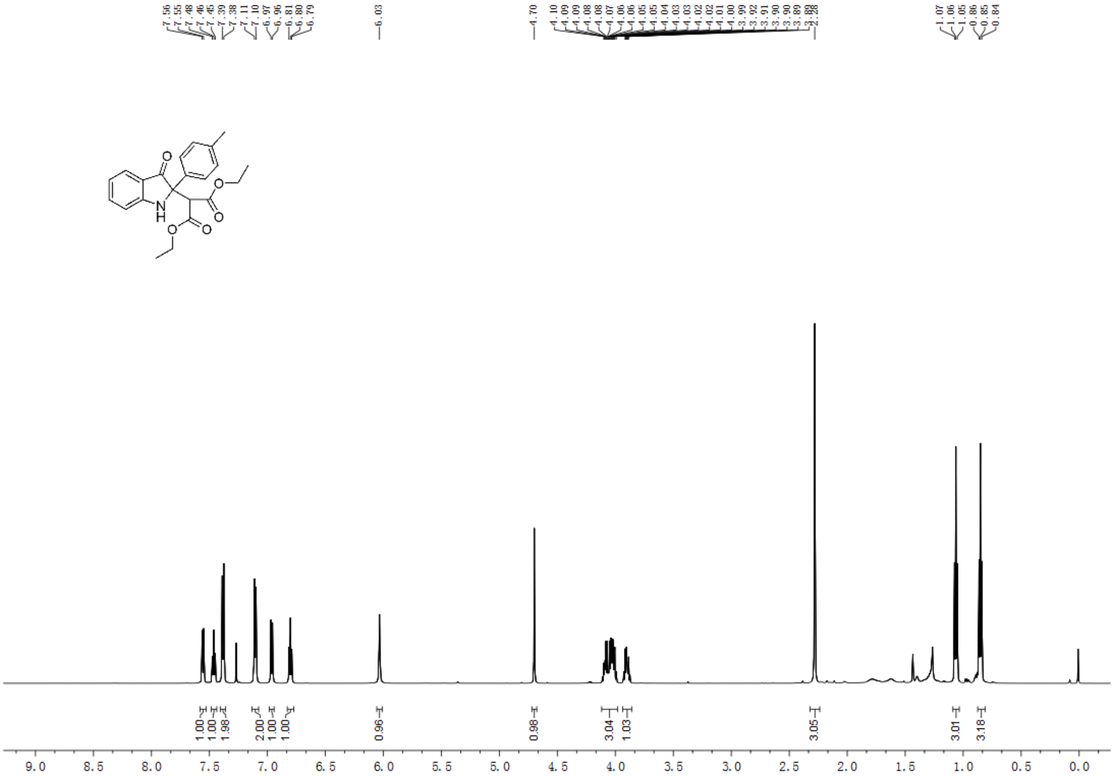

^{13}C NMR spectrum of compound 3h in $CDCl_3$ (151 MHz)

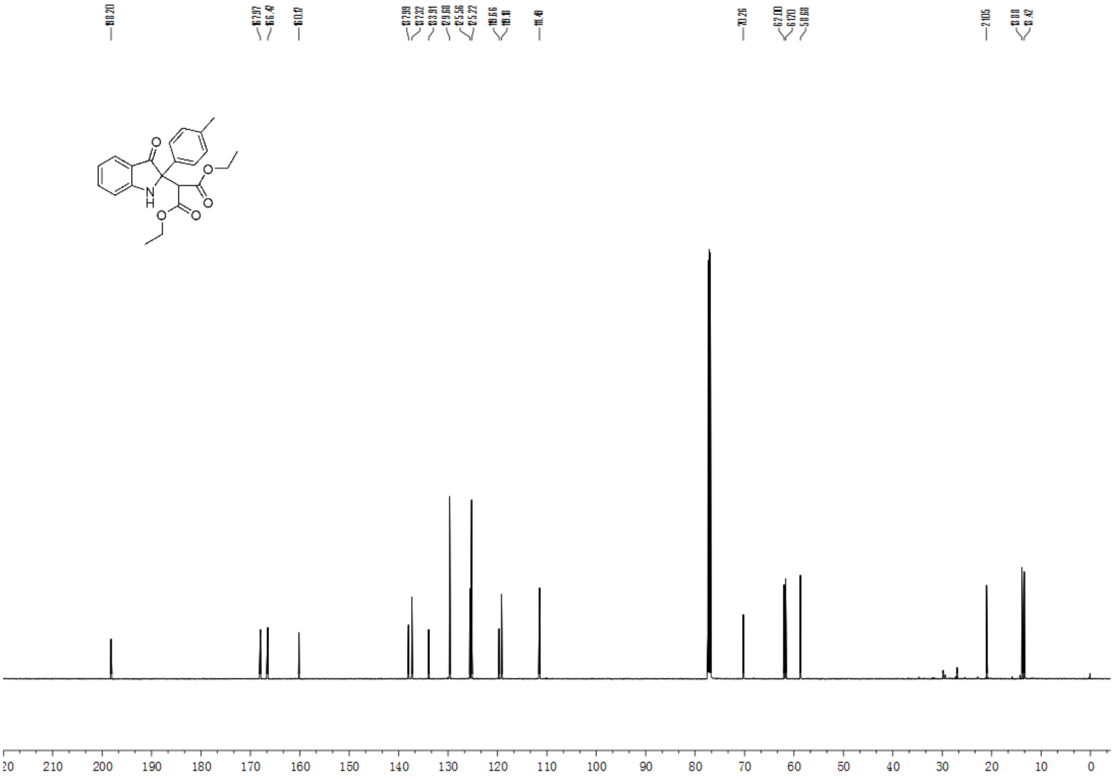

^{1}H NMR spectrum of compound 3i in $CDCl_3$ (600 MHz)

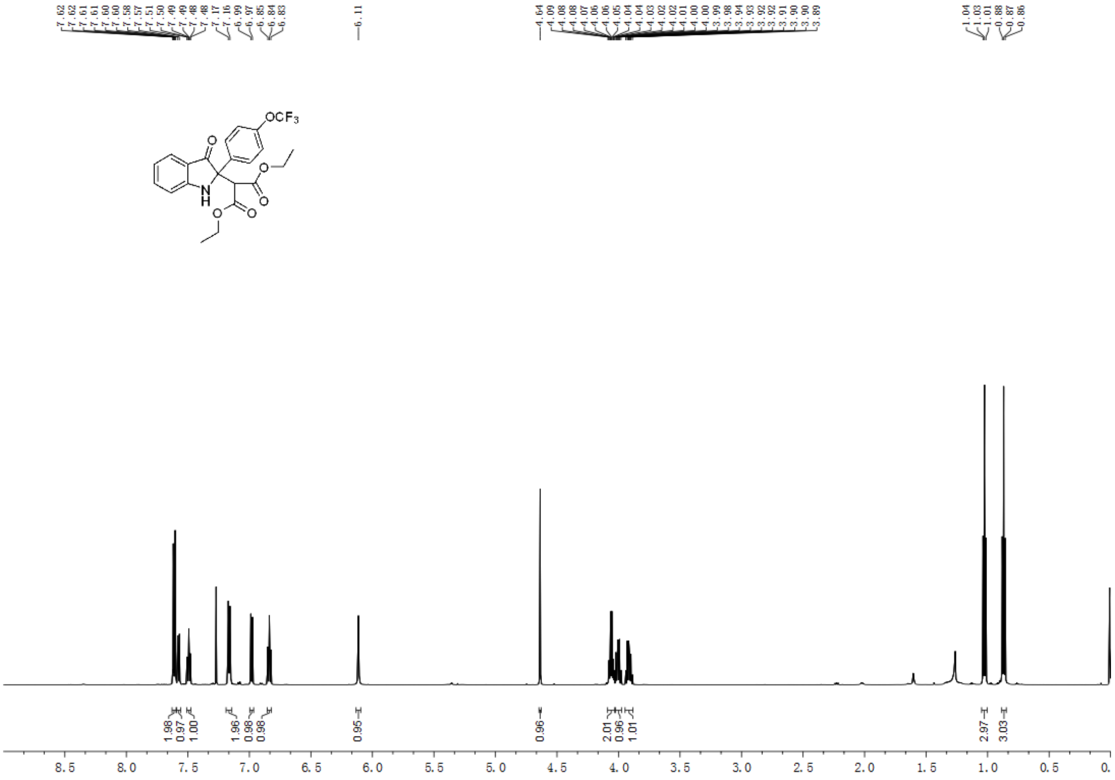

^{13}C NMR spectrum of compound 3i in $CDCl_3$ (151 MHz)

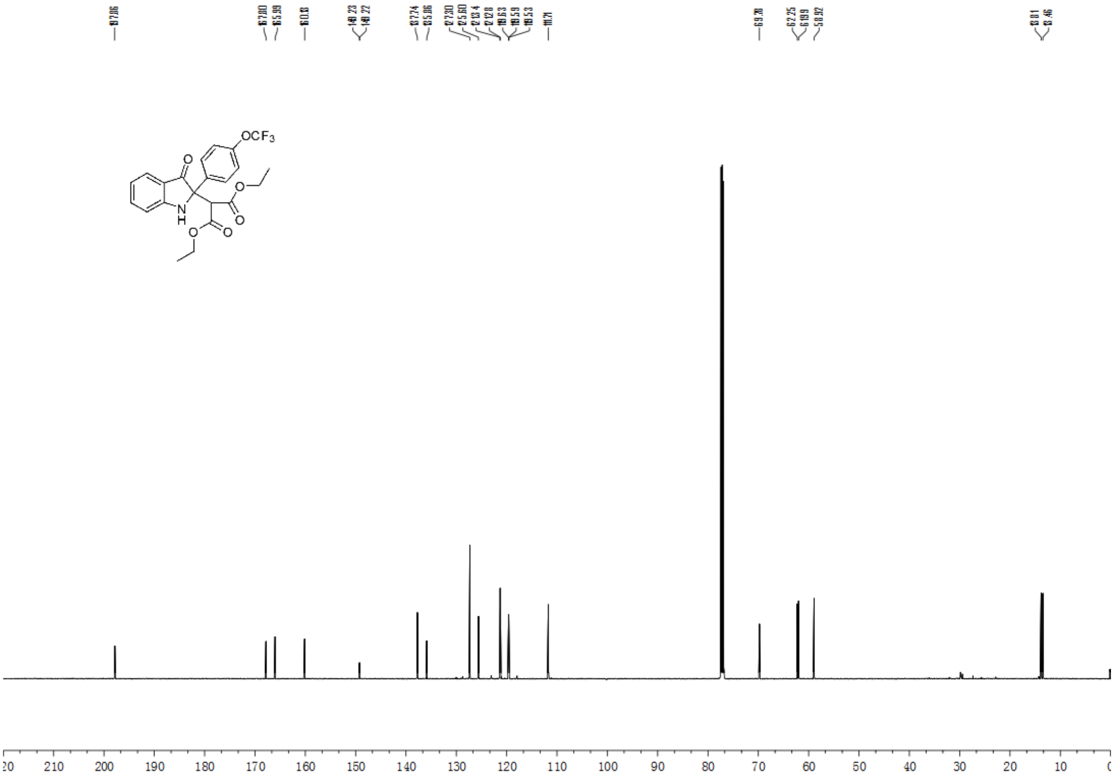

^{1}H NMR spectrum of compound 3j in $CDCl_3$ (600 MHz)

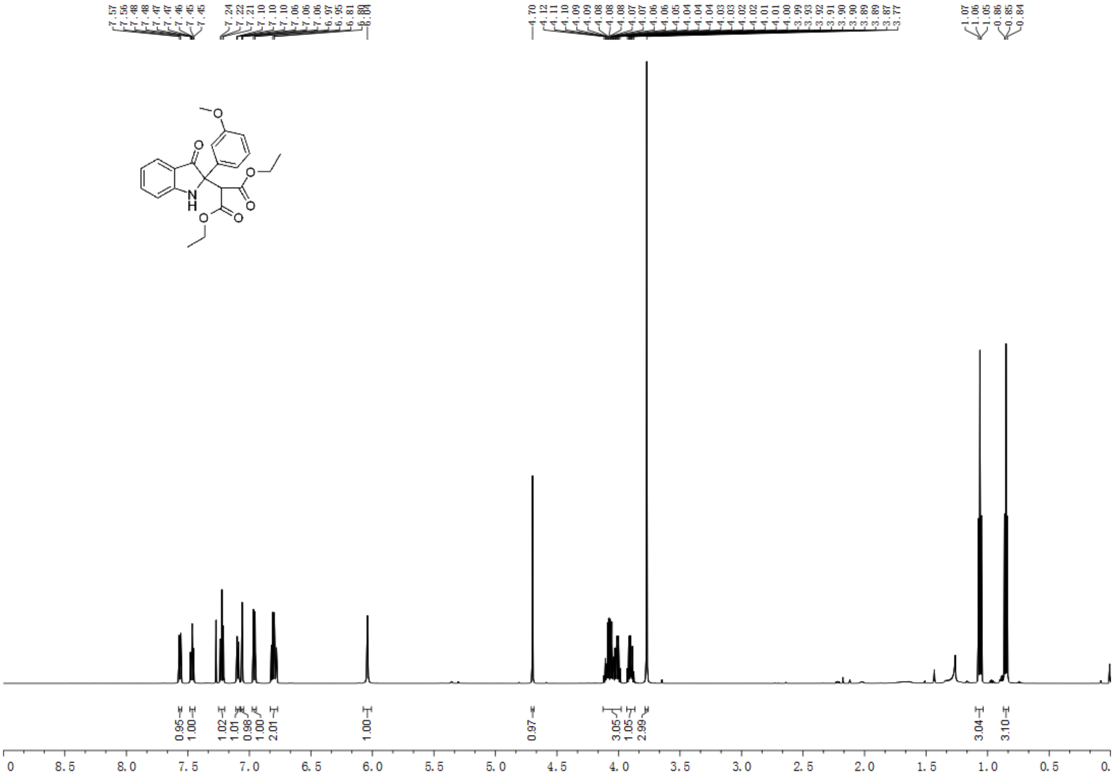

^{13}C NMR spectrum of compound 3j in $CDCl_3$ (151 MHz)

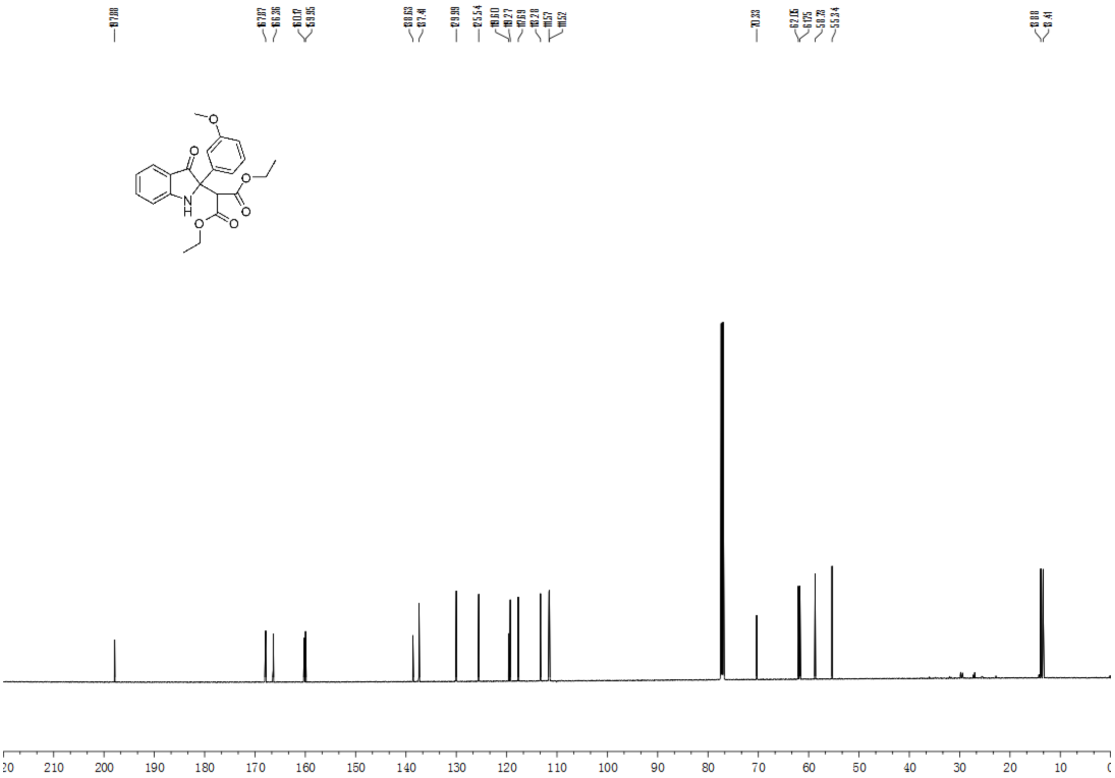

^{1}H NMR spectrum of compound 3k in $CDCl_3$ (600 MHz)

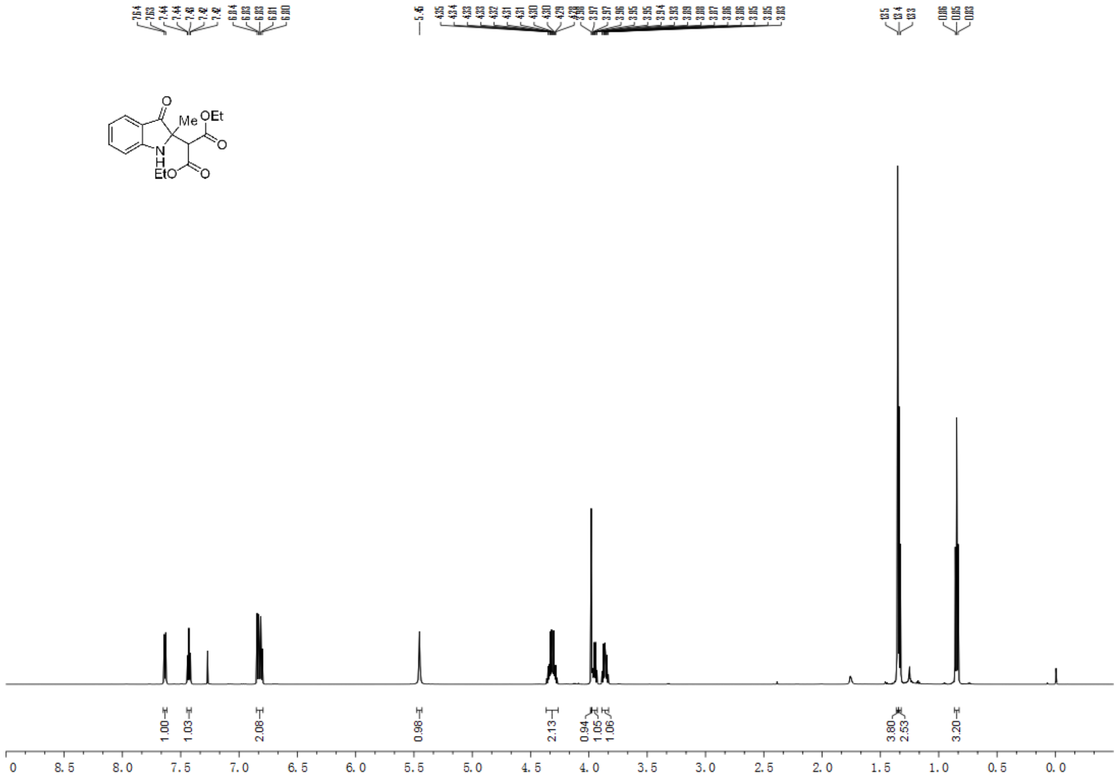

^{13}C NMR spectrum of compound 3k in $CDCl_3$ (151 MHz)

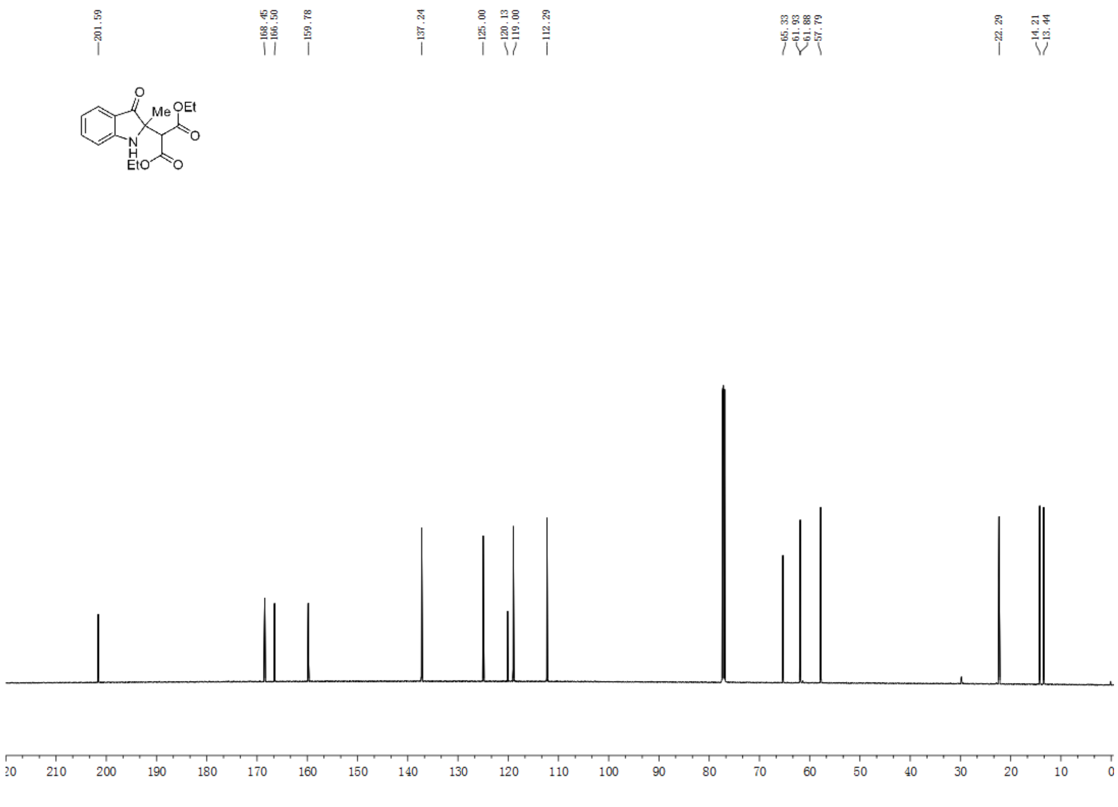

^{1}H NMR spectrum of compound 3l in $CDCl_3$ (600 MHz)

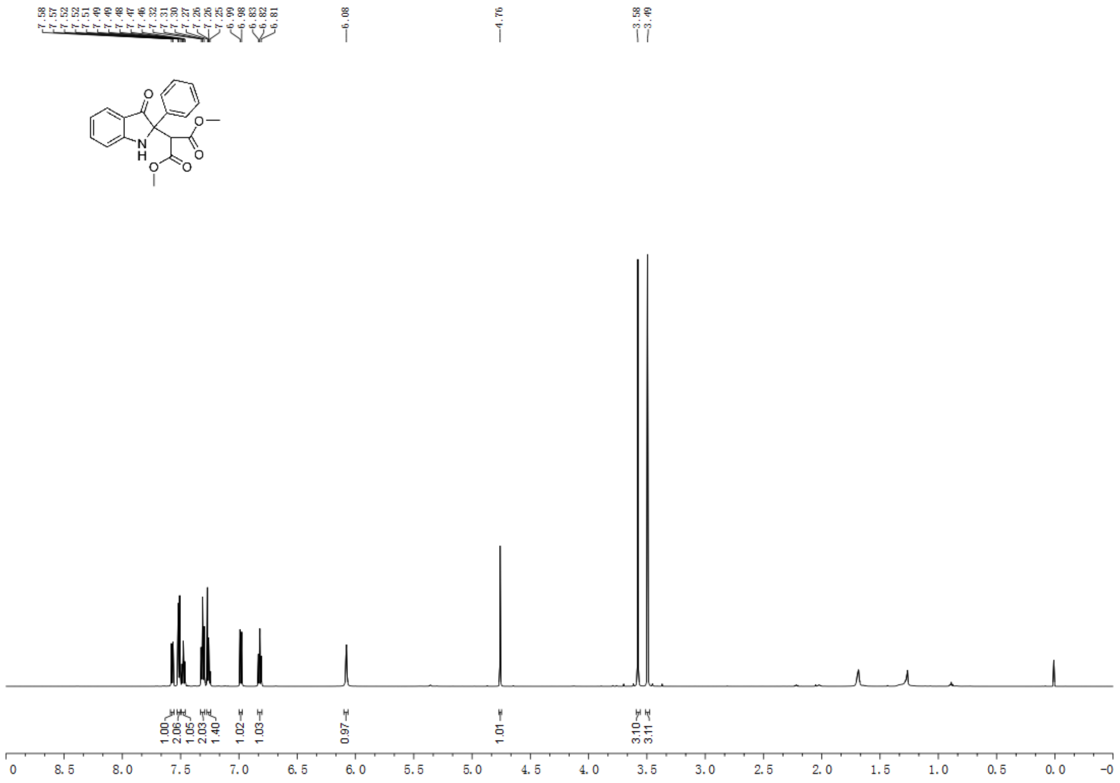

^{13}C NMR spectrum of compound 3l in $CDCl_3$ (151 MHz)

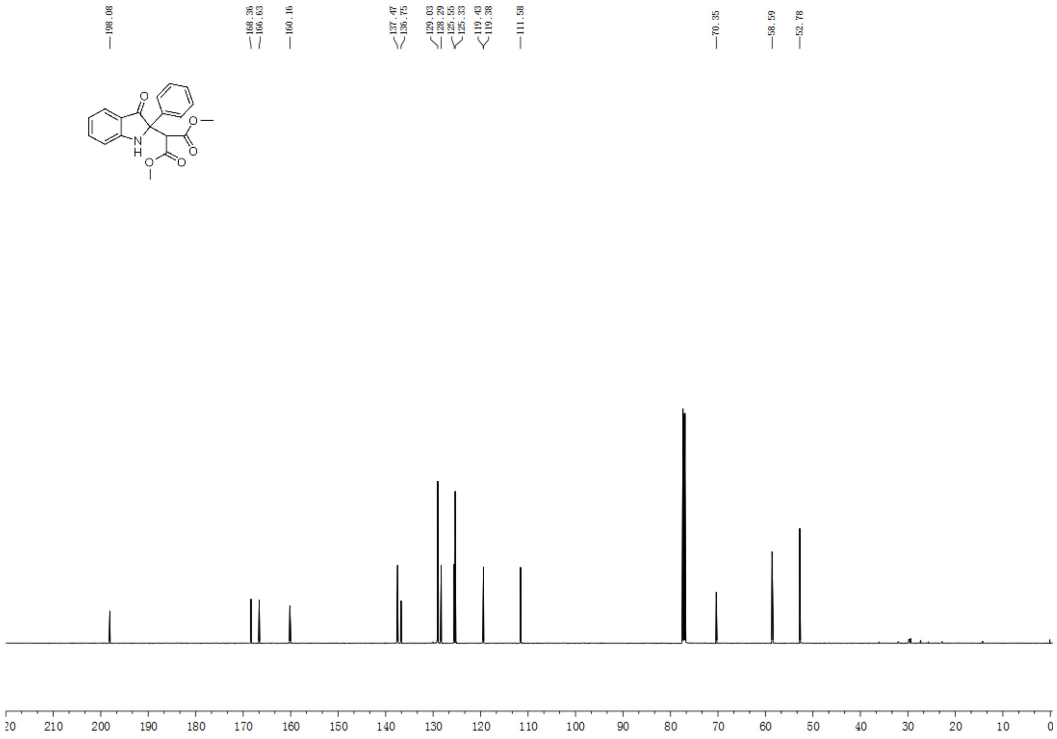

^{1}H NMR spectrum of compound 3m in $CDCl_3$ (600 MHz)

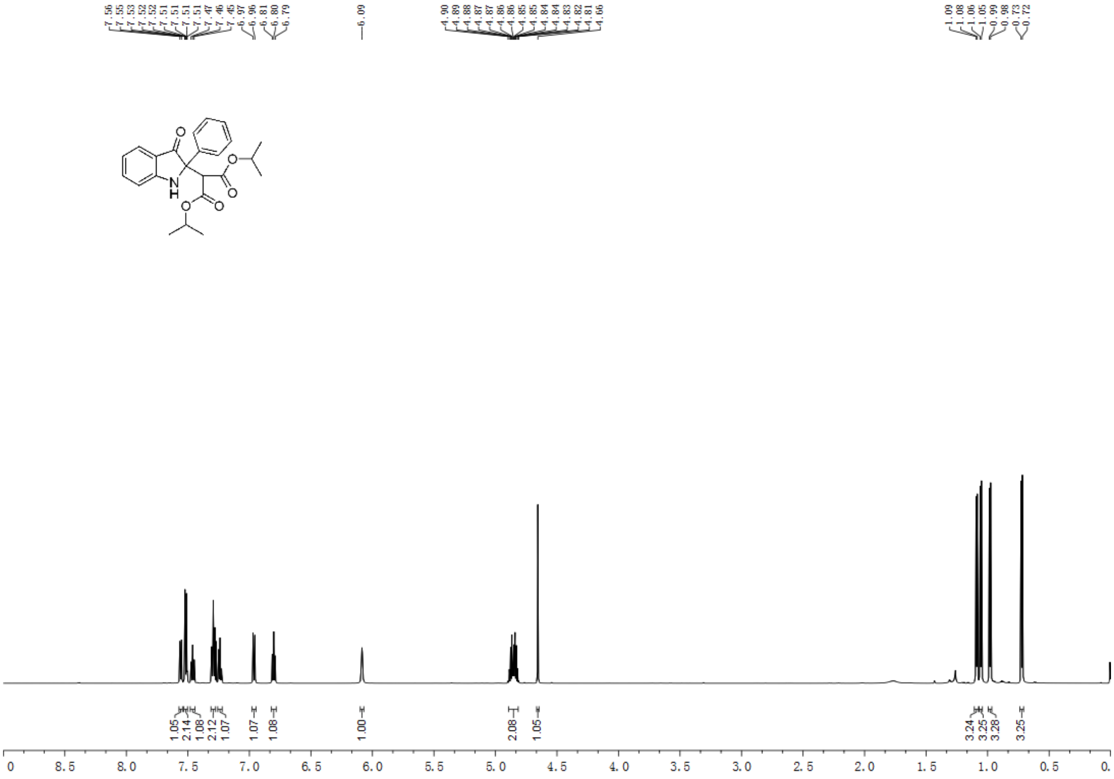

^{13}C NMR spectrum of compound 3m in $CDCl_3$ (151 MHz)

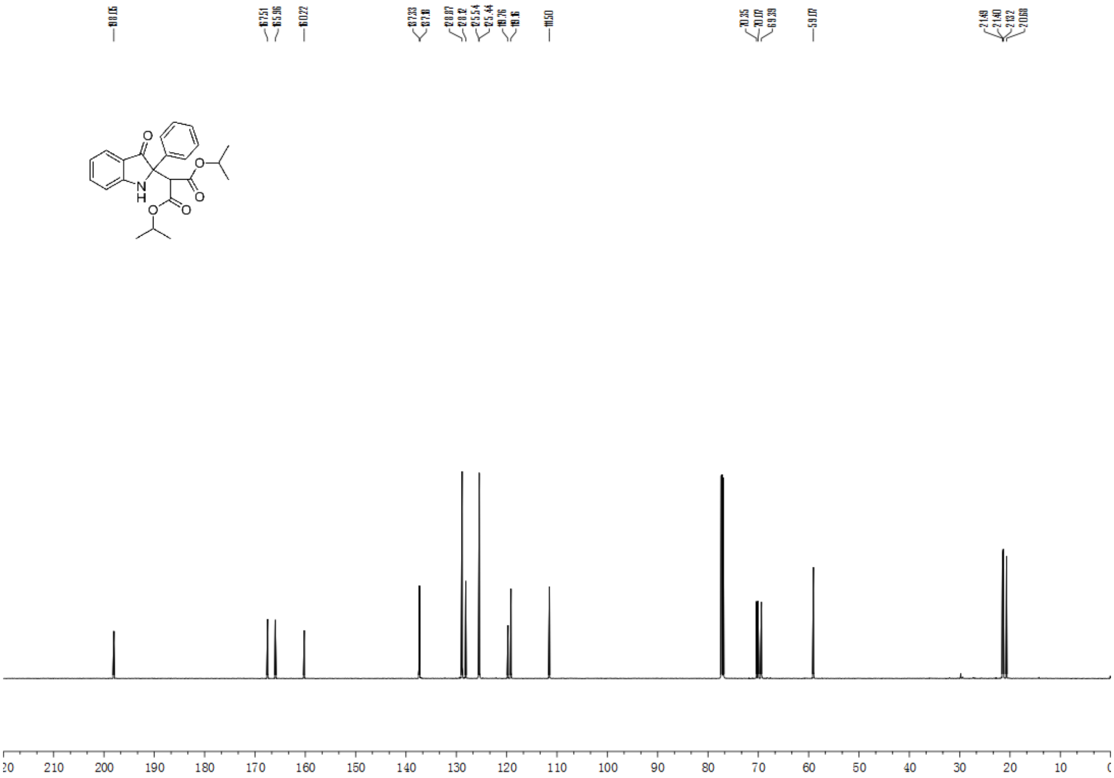

^{1}H NMR spectrum of compound 3n in $CDCl_3$ (600 MHz)

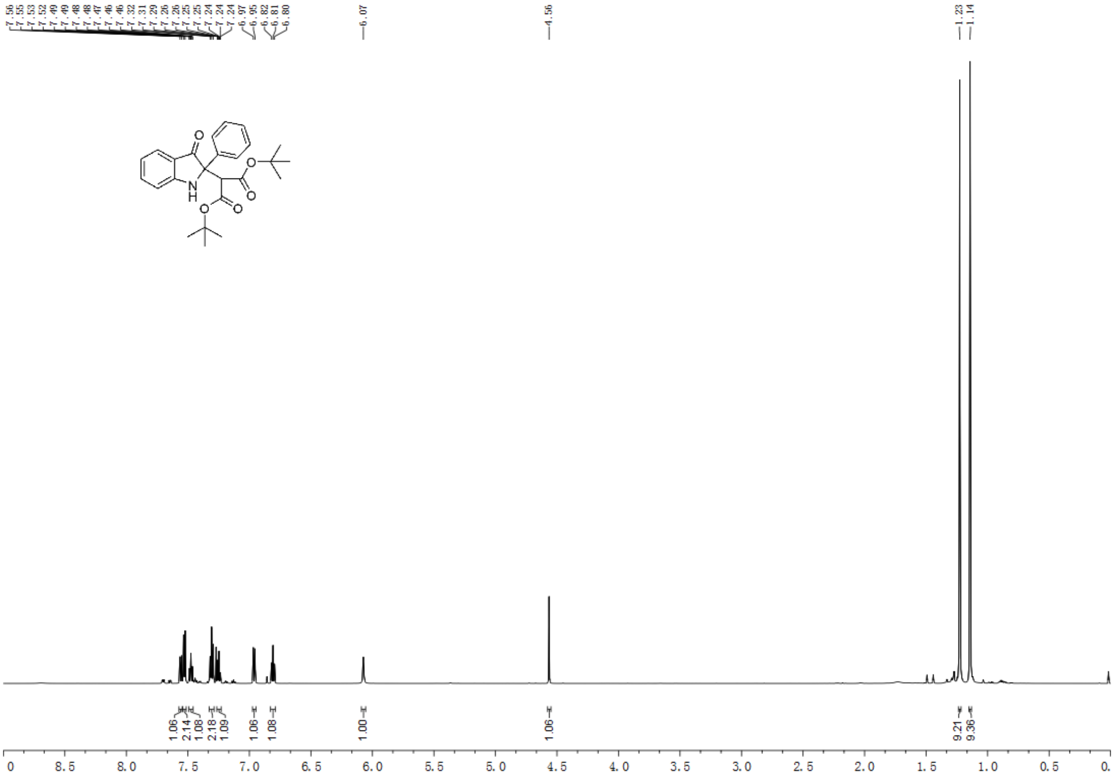

^{13}C NMR spectrum of compound 3n in $CDCl_3$ (151 MHz)

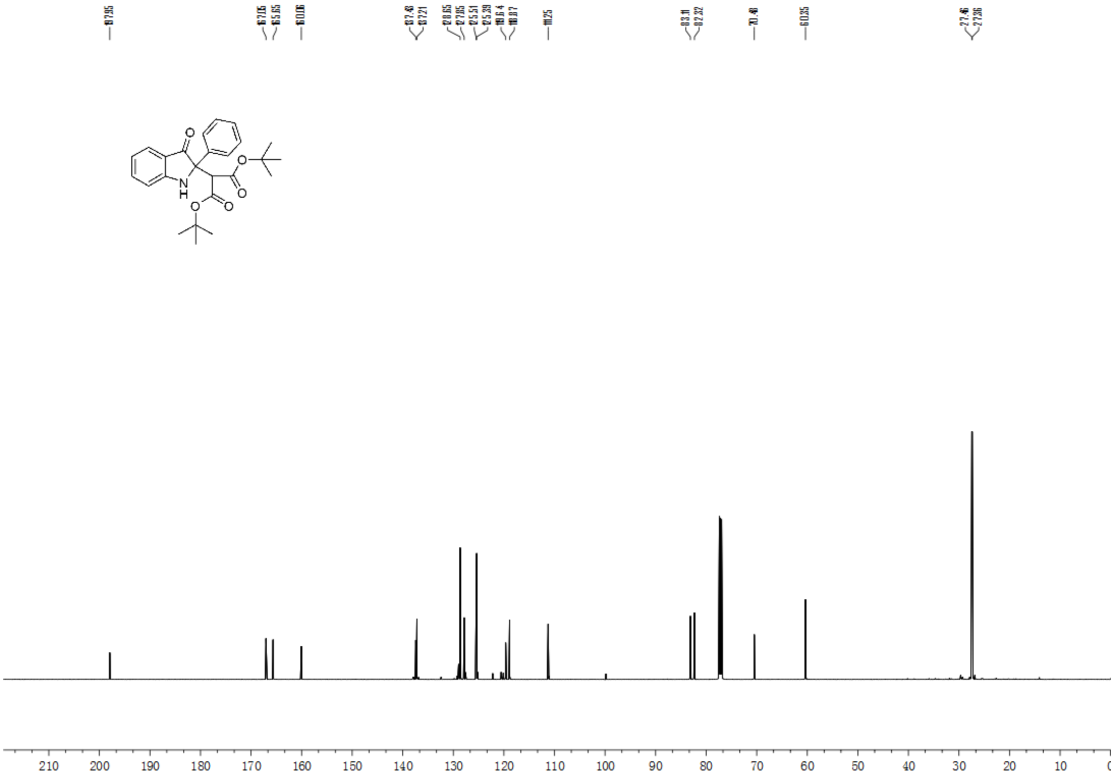

^{1}H NMR spectrum of compound 3o in $CDCl_3$ (600 MHz)

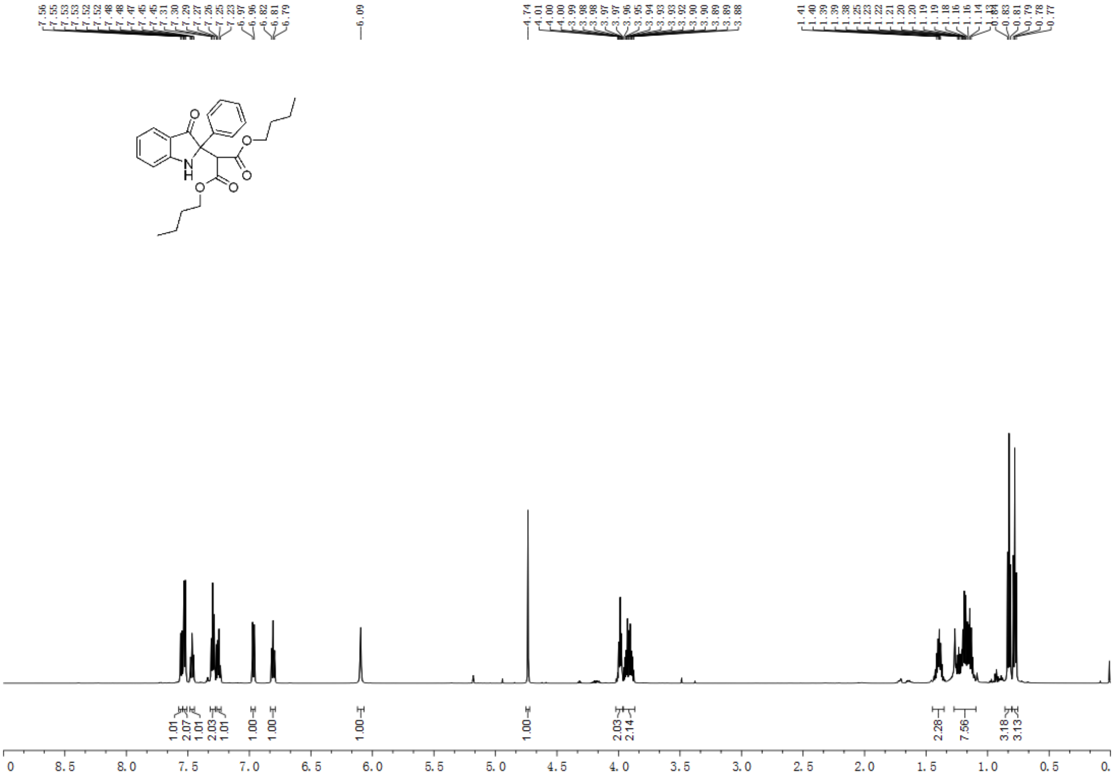

^{13}C NMR spectrum of compound 3o in $CDCl_3$ (151 MHz)

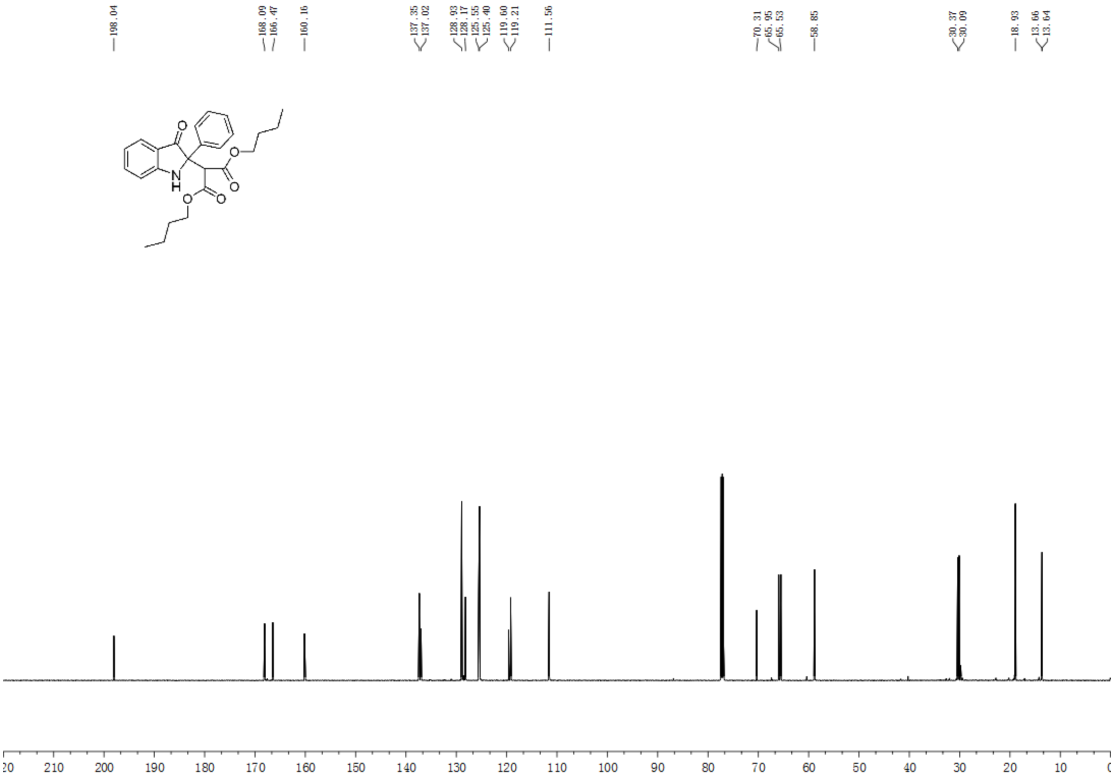

^{1}H NMR spectrum of compound 3p in $CDCl_3$ (600 MHz)

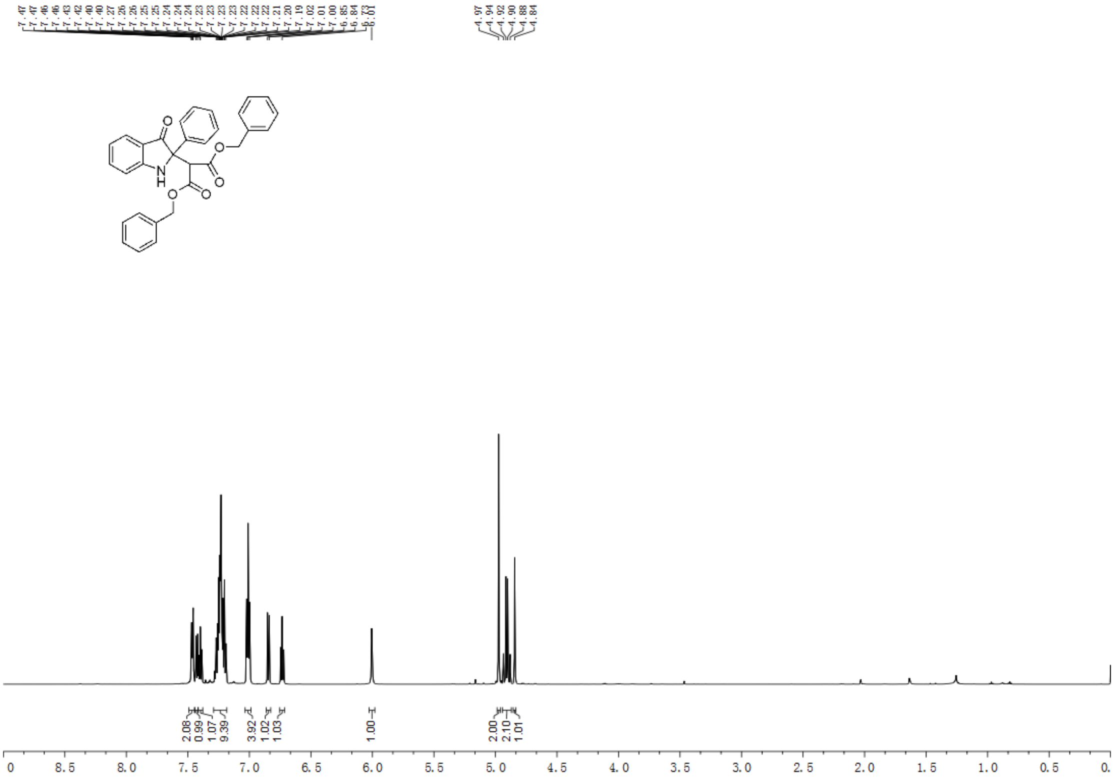

^{13}C NMR spectrum of compound 3p in $CDCl_3$ (151 MHz)

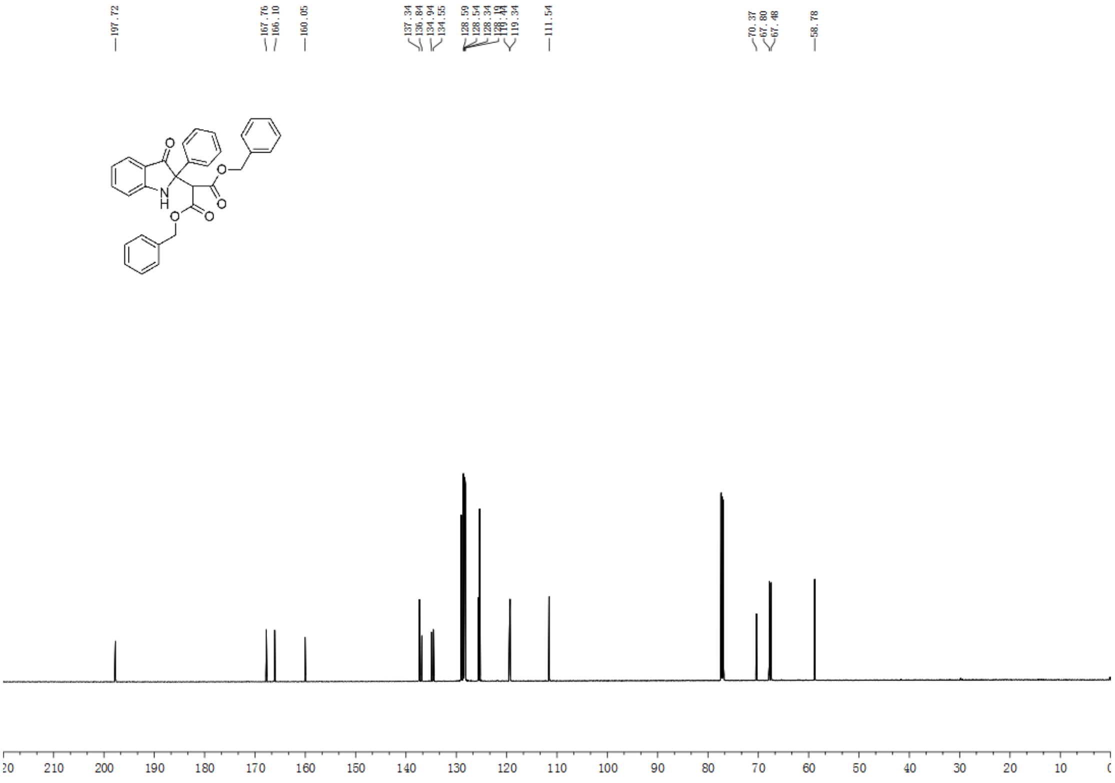

^{1}H NMR spectrum of compound 3q in $CDCl_3$ (600 MHz)

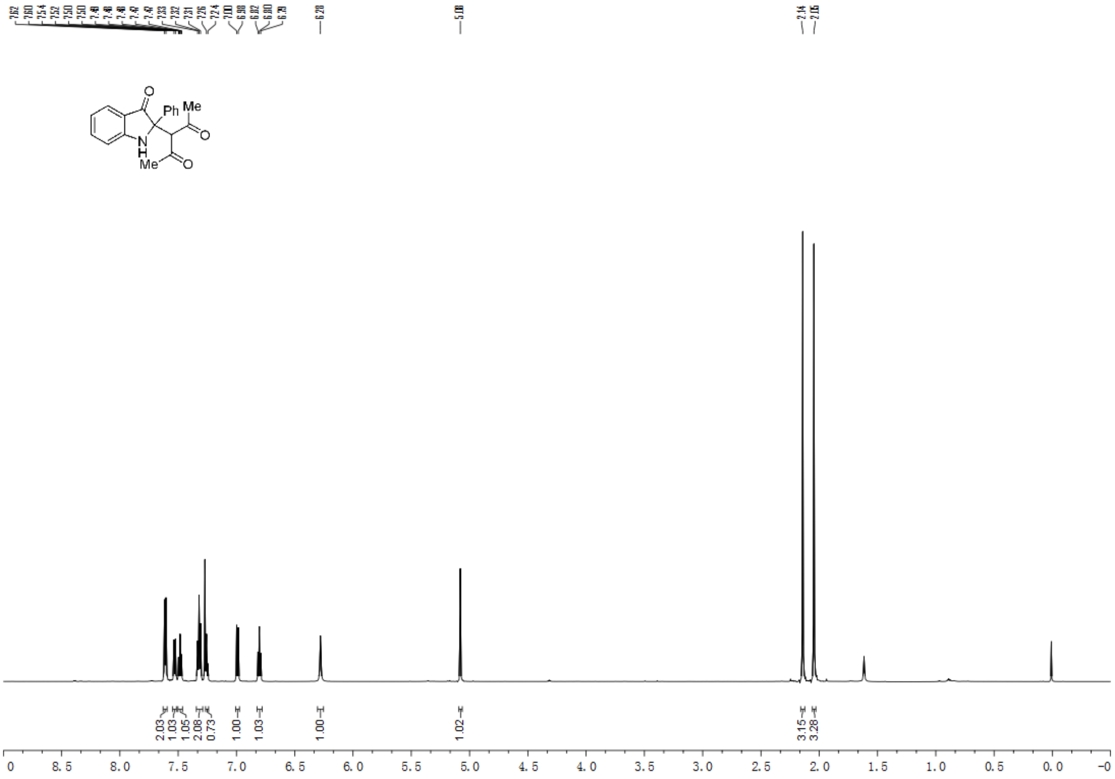

^{13}C NMR spectrum of compound 3q in $CDCl_3$ (151 MHz)

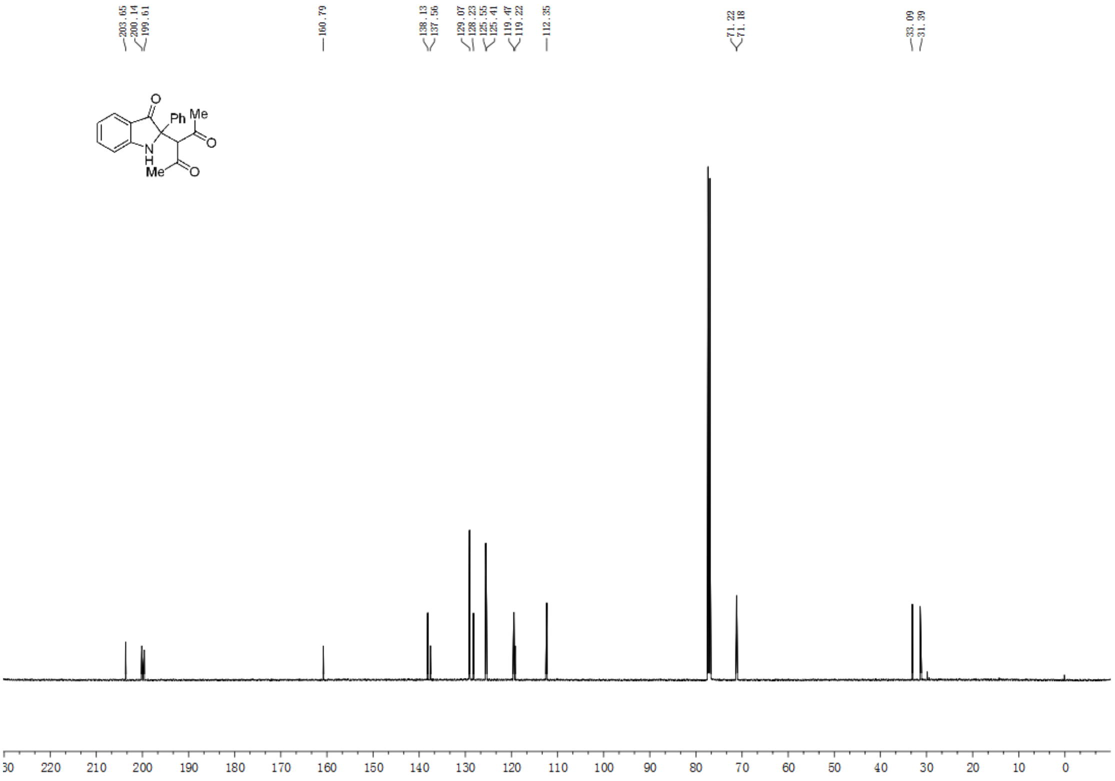

^{1}H NMR spectrum of compound 5a in acetone-d_6 (600 MHz)

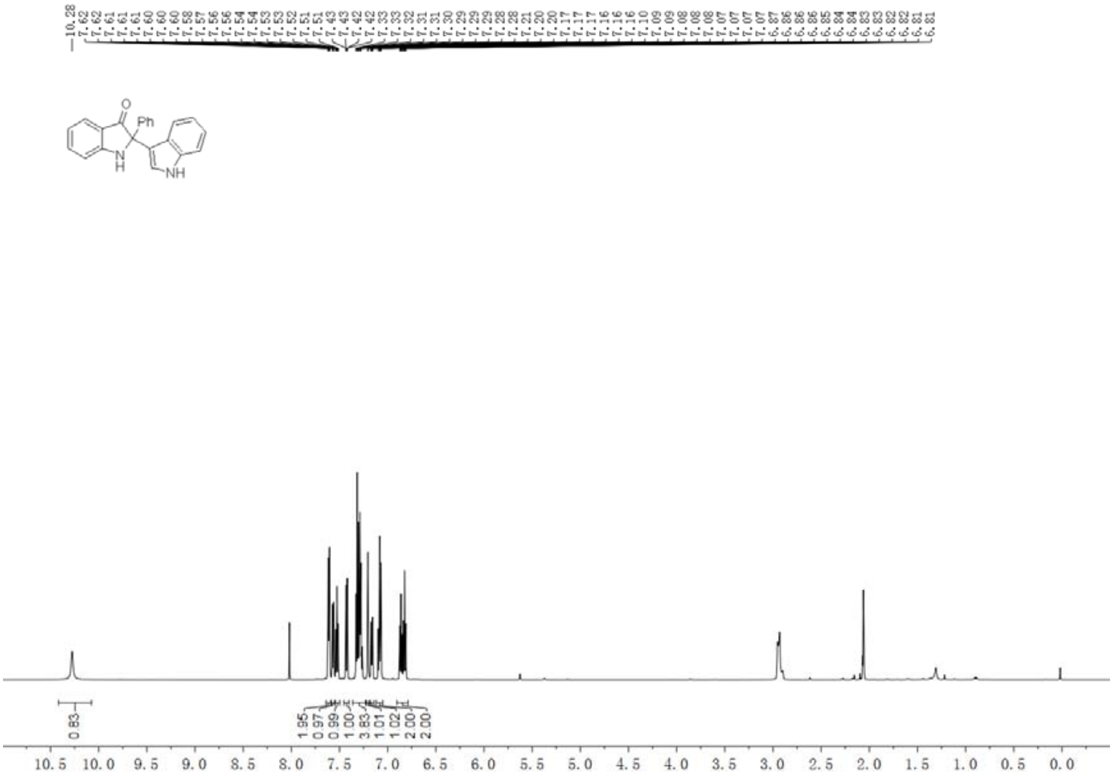

^{13}C NMR spectrum of compound 5a in acetone-d_6 (151 MHz)

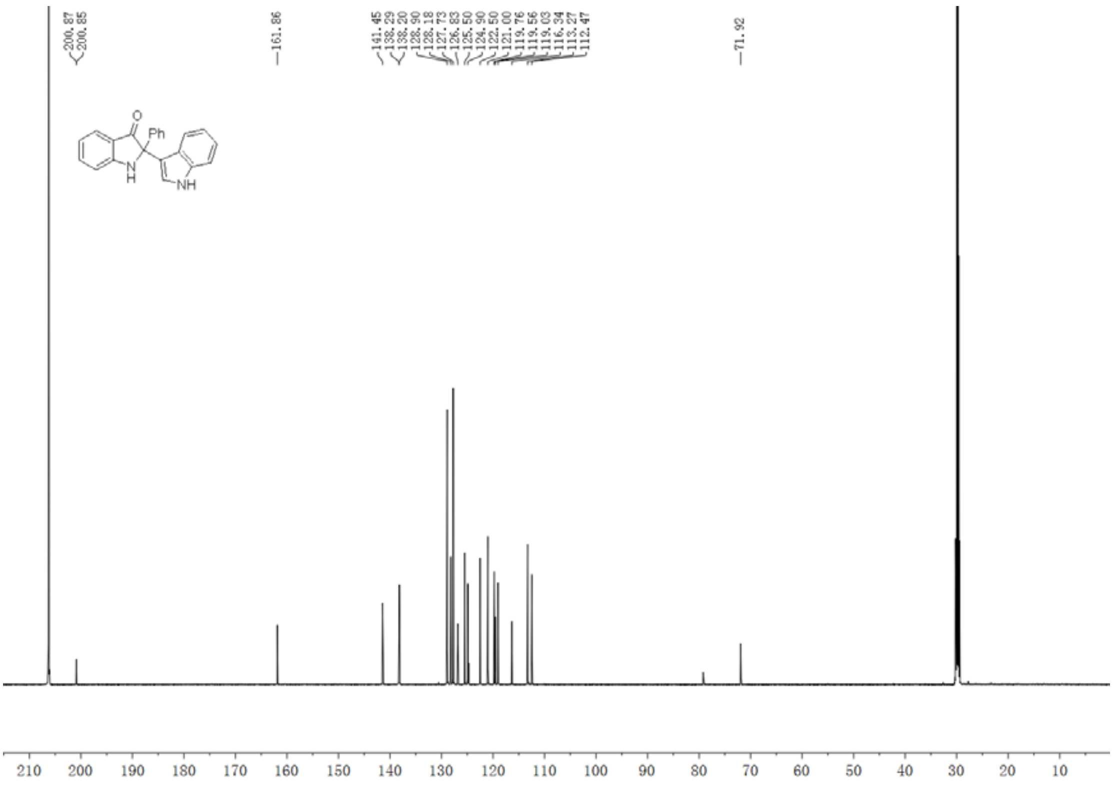

^{1}H NMR spectrum of compound 5b in $CDCl_3$ (600 MHz)

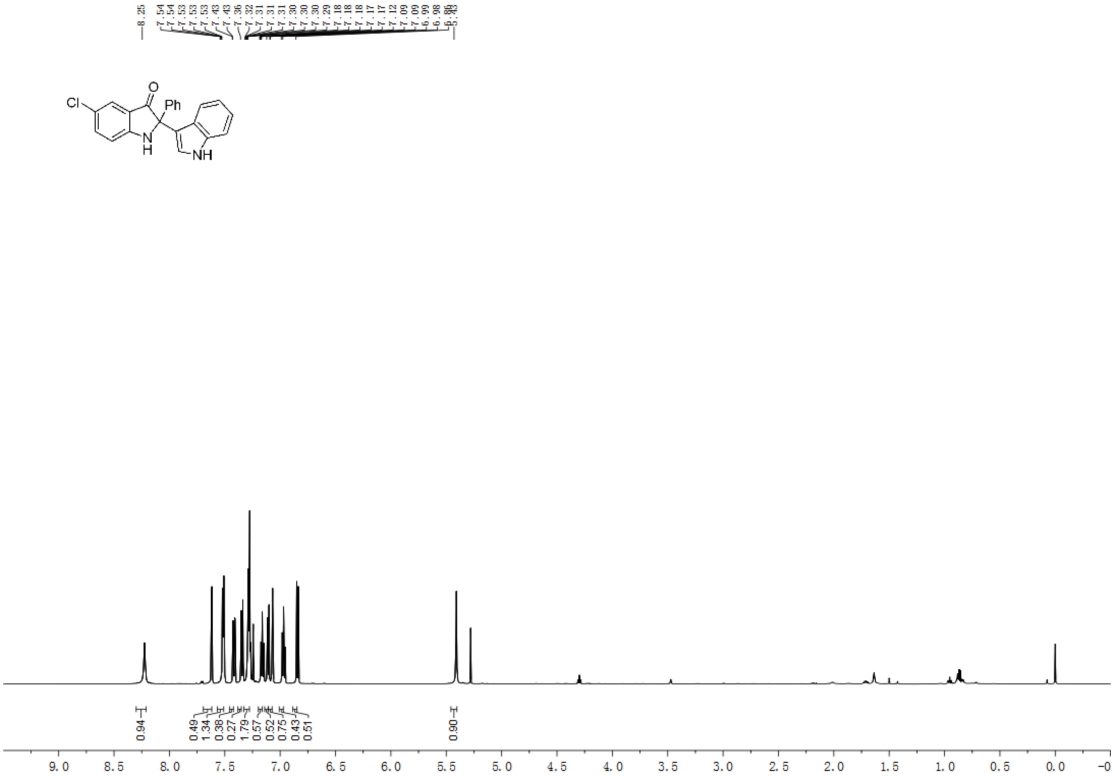

^{13}C NMR spectrum of compound 5b in $CDCl_3$ (151 MHz)

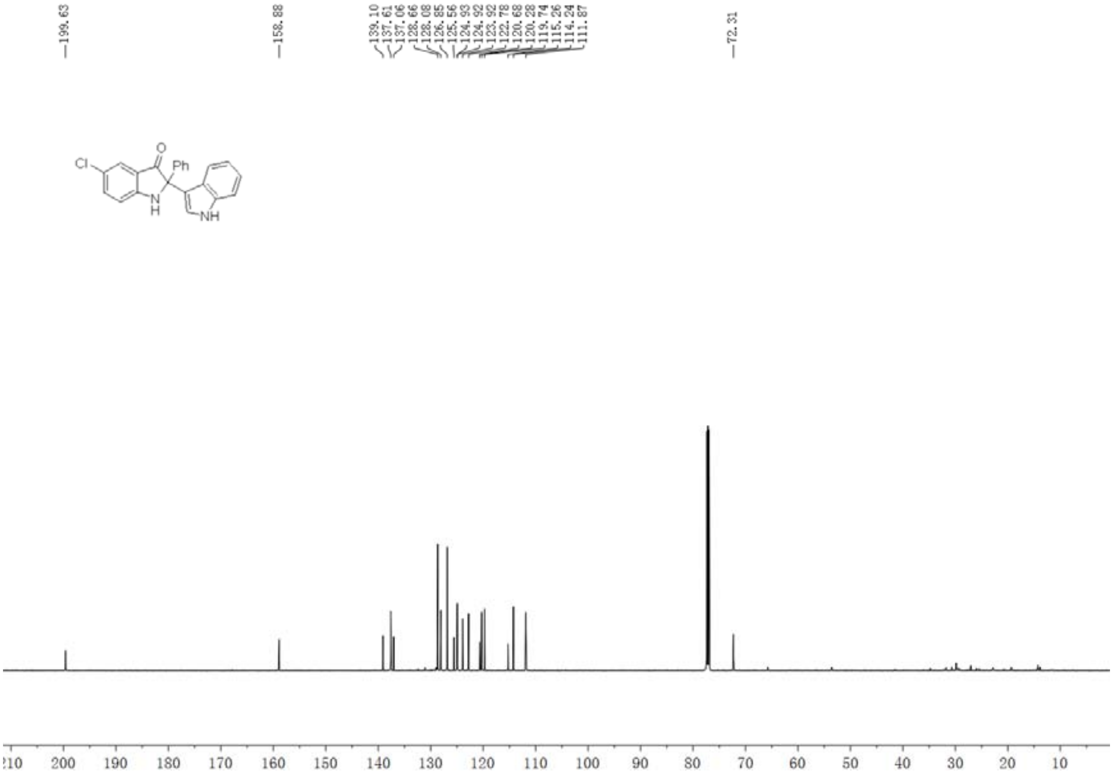

^{1}H NMR spectrum of compound 5c in $CDCl_3$ (600 MHz)

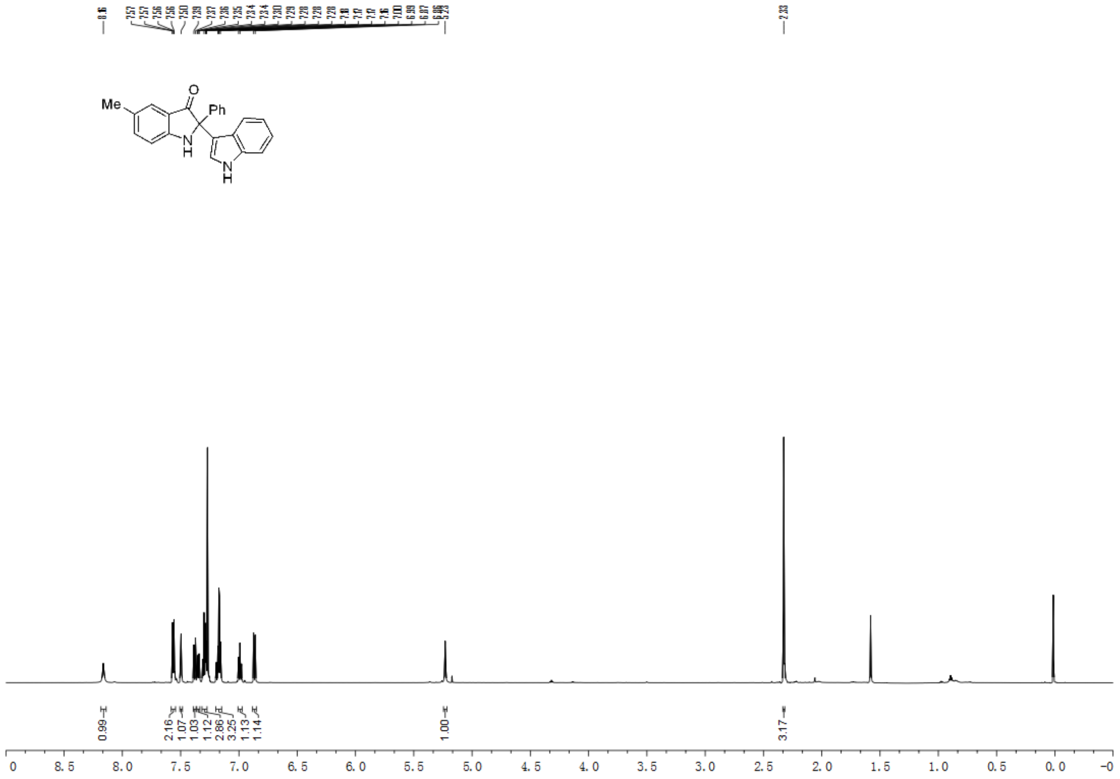

^{13}C NMR spectrum of compound 5c in $CDCl_3$ (151 MHz)

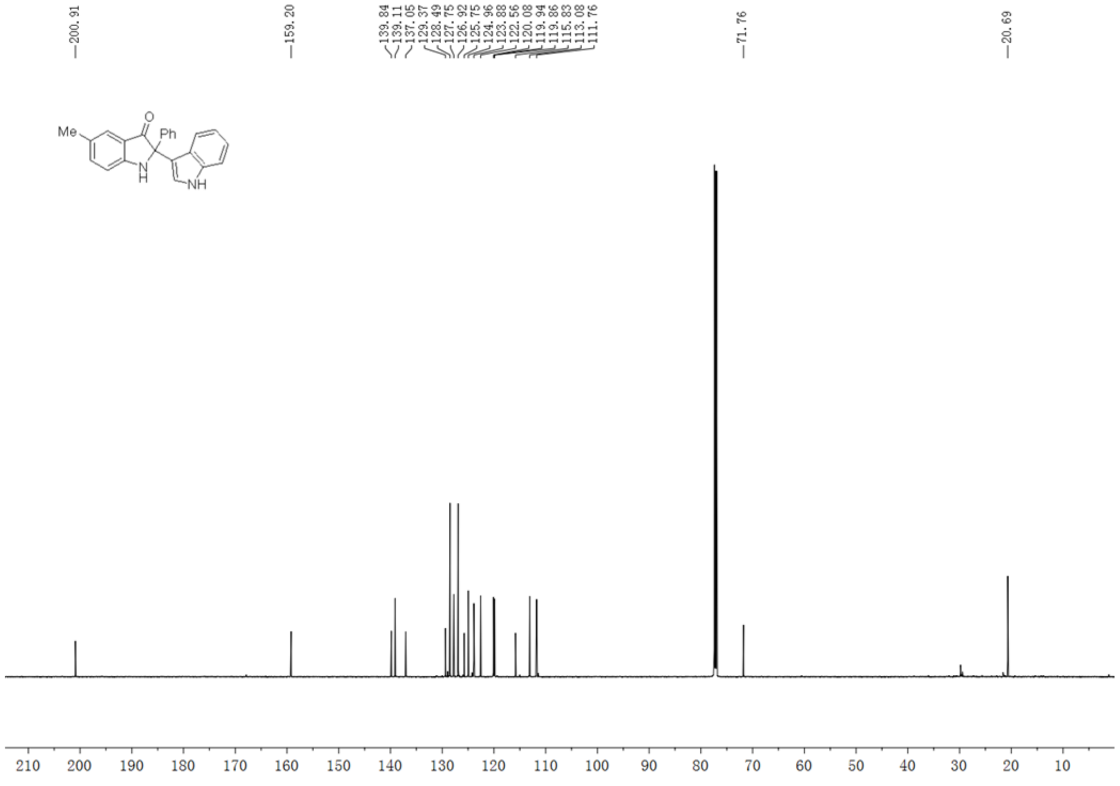

^{1}H NMR spectrum of compound 5d in $CDCl_3$ (600 MHz)

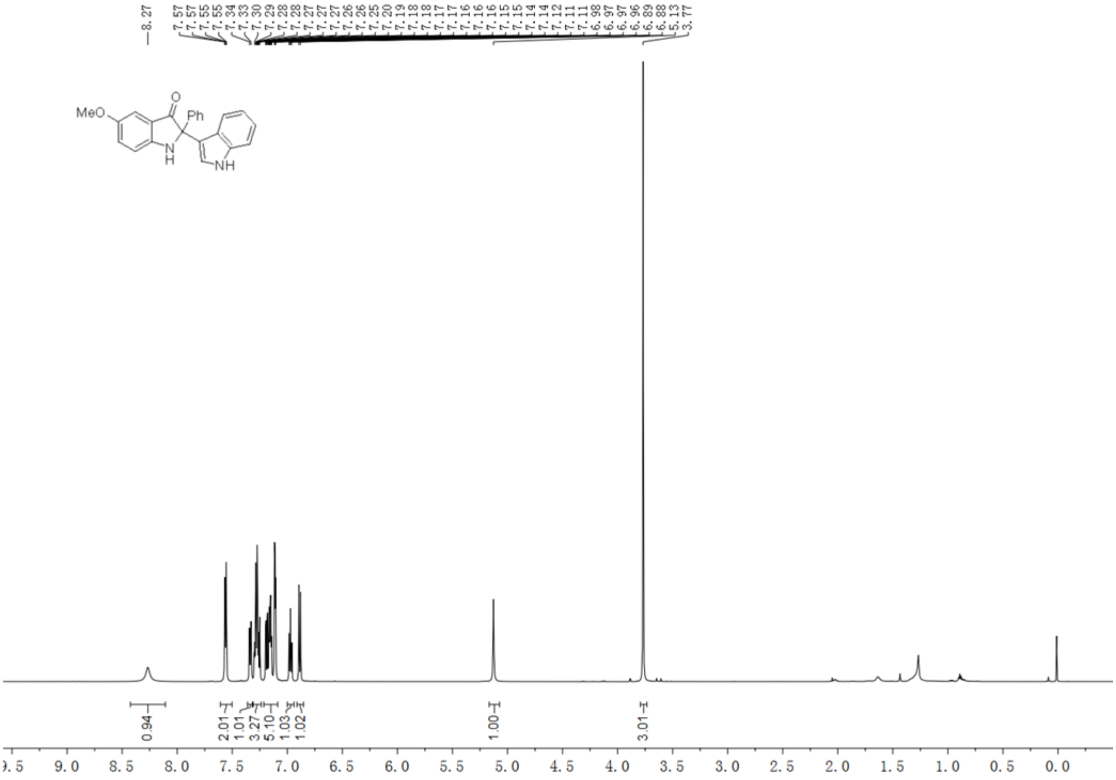

^{13}C NMR spectrum of compound 5d in $CDCl_3$ (151 MHz)

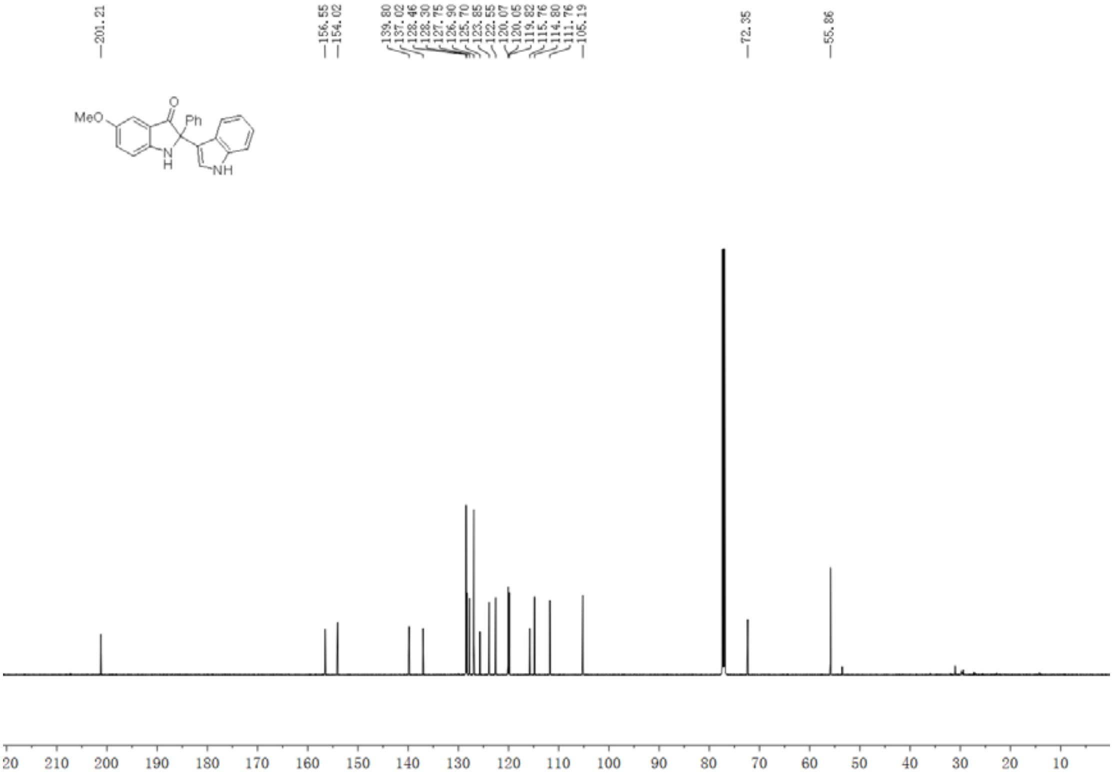

^{1}H NMR spectrum of compound 5e in acetone-d_6 (600 MHz)

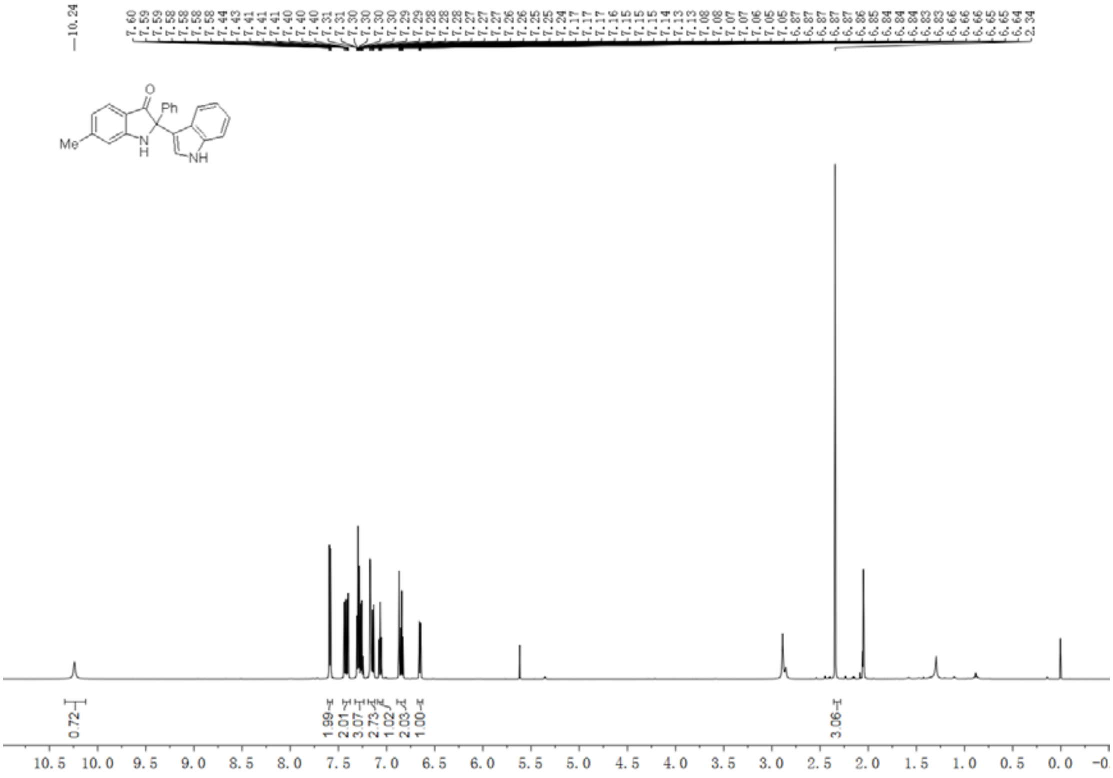

^{13}C NMR spectrum of compound 5e in acetone-d_6 (151 MHz)

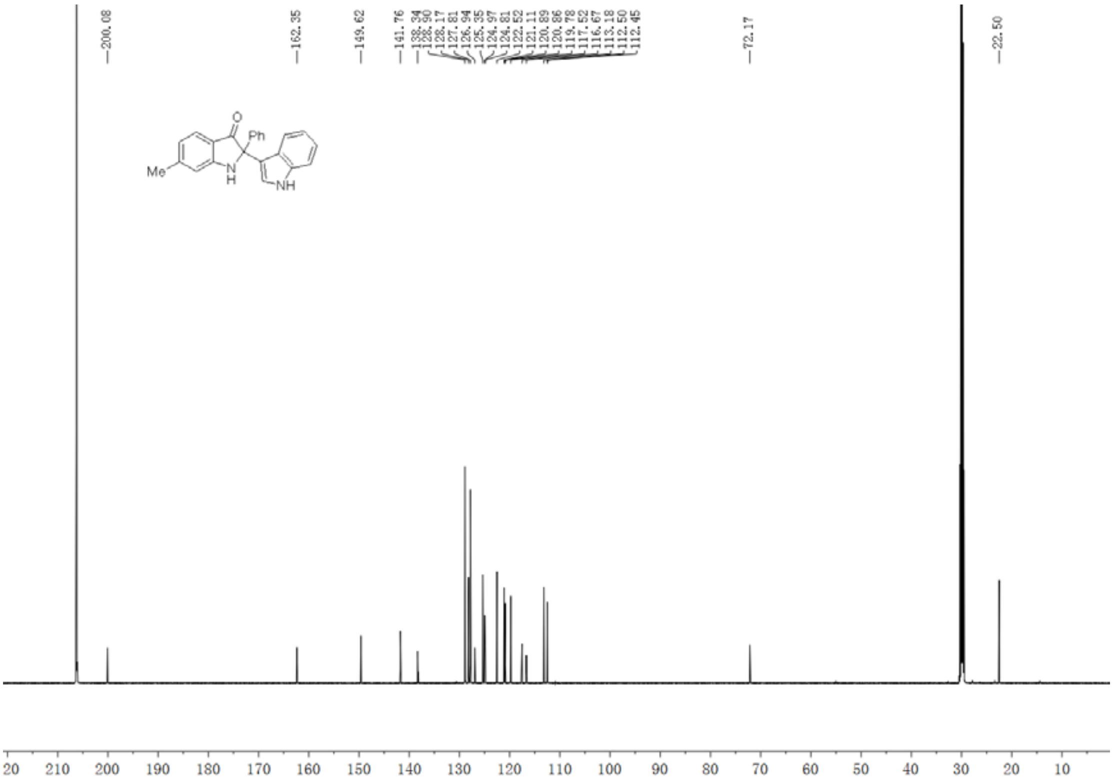

^{1}H NMR spectrum of compound 5f in $CDCl_3$ (600 MHz)

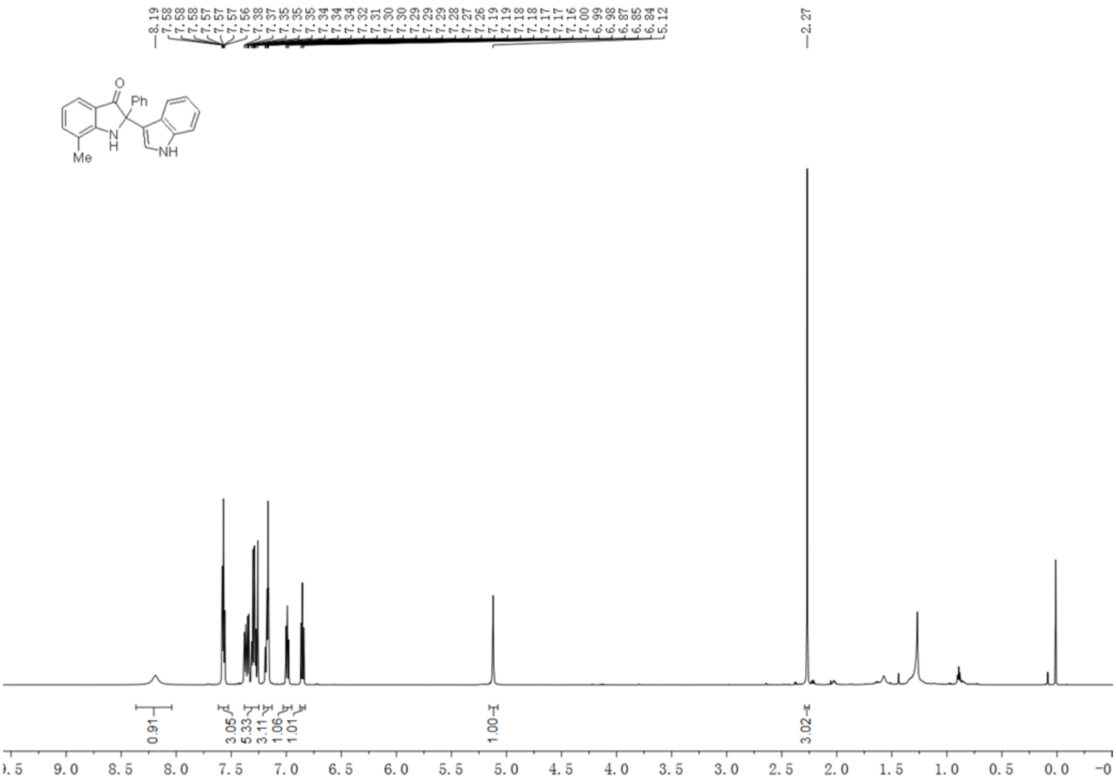

^{13}C NMR spectrum of compound 5f in $CDCl_3$ (151 MHz)

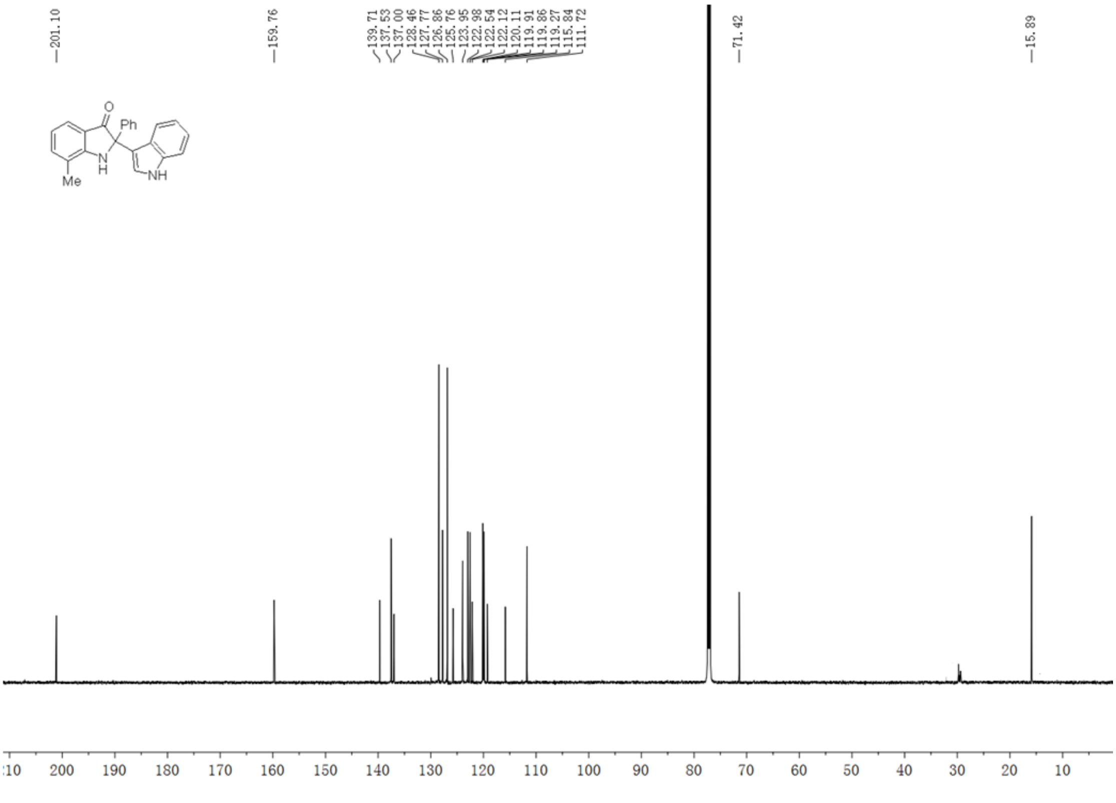

^{1}H NMR spectrum of compound 5g in $CDCl_3$ (600 MHz)

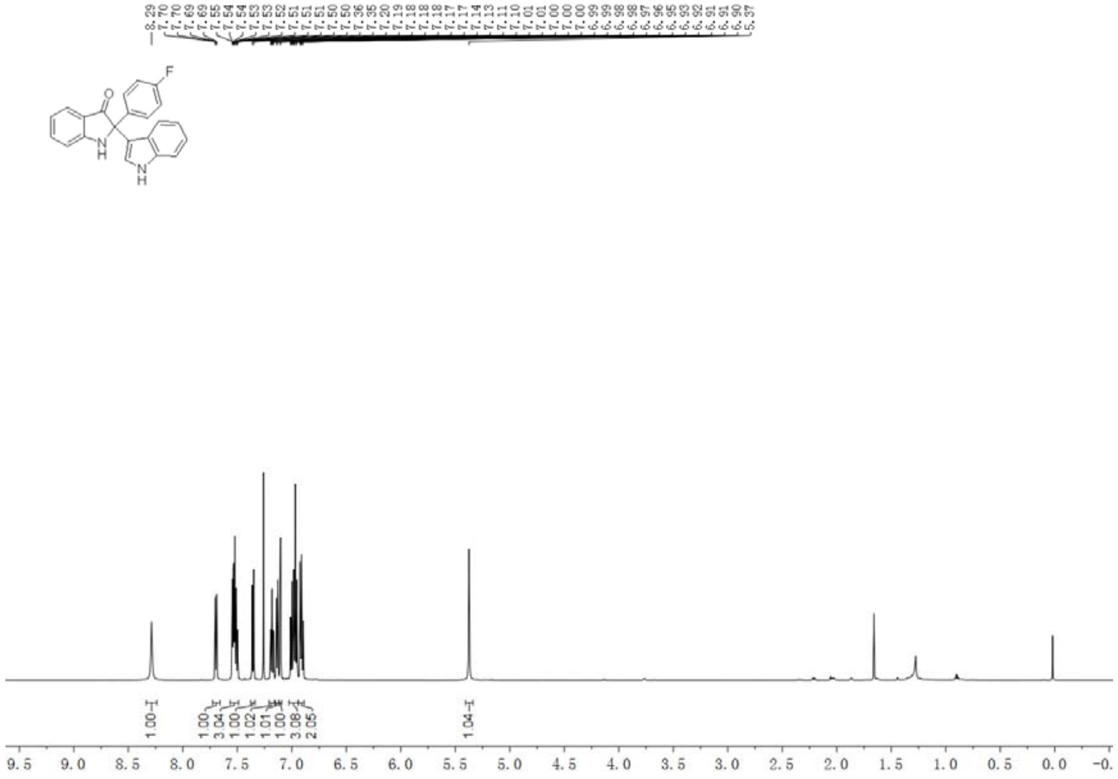

^{13}C NMR spectrum of compound 5g in $CDCl_3$ (151 MHz)

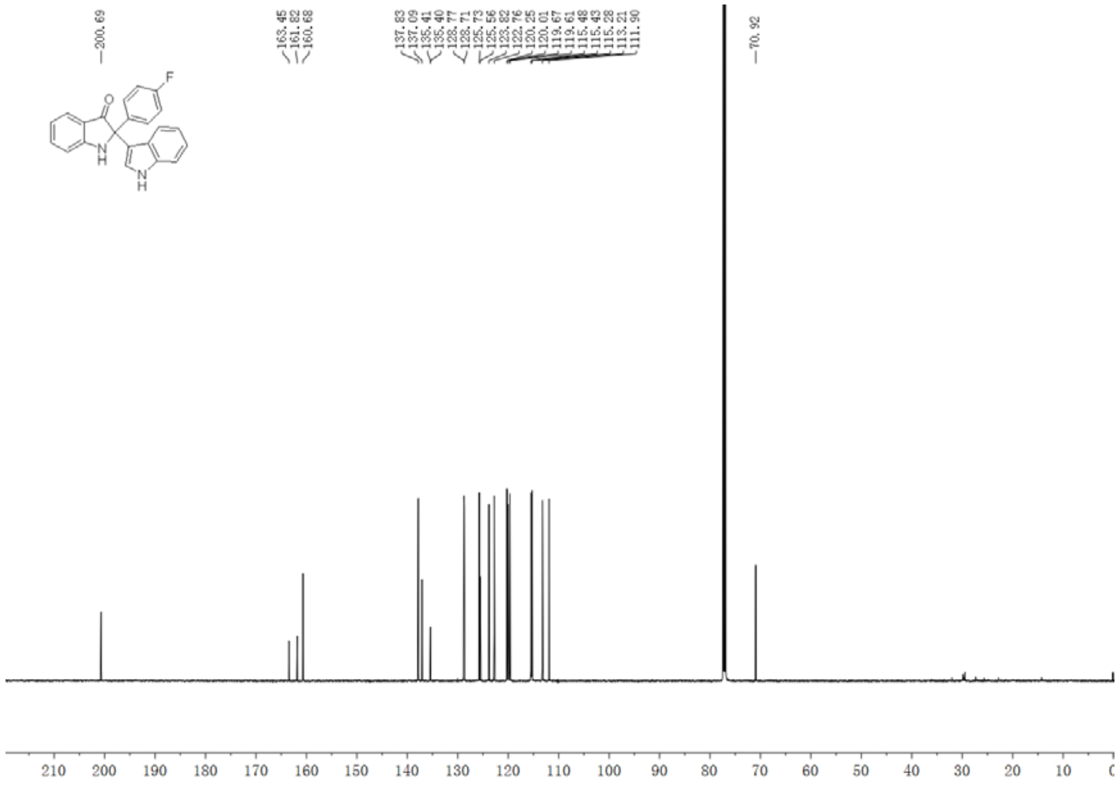

^{1}H NMR spectrum of compound 5h in acetone-d_6 (600 MHz)

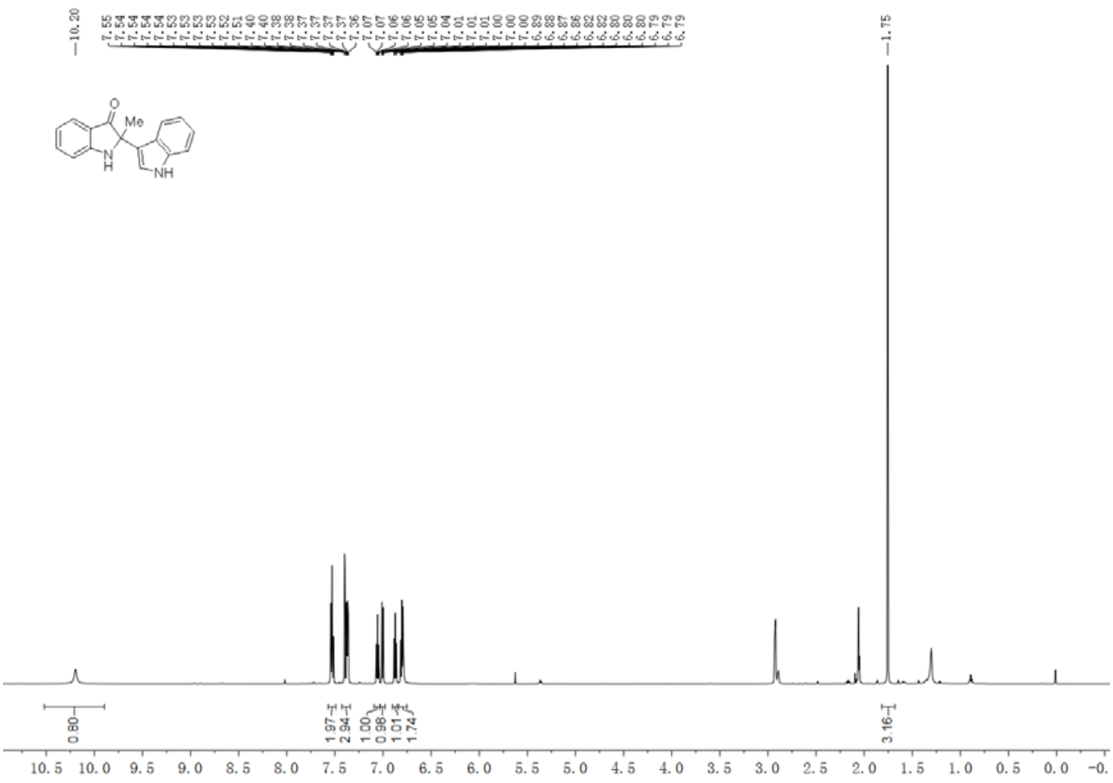

^{13}C NMR spectrum of compound 5h in acetone-d_6 (151 MHz)

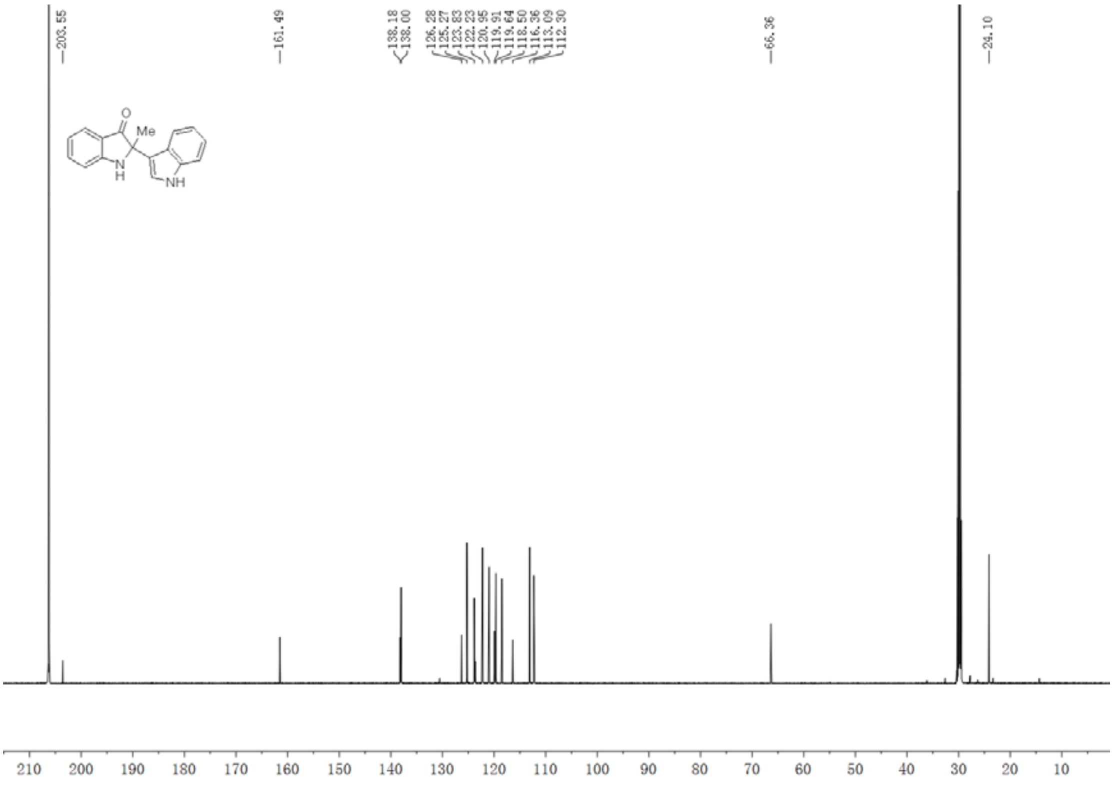

^{1}H NMR spectrum of compound 5i in acetone-d_6 (600 MHz)

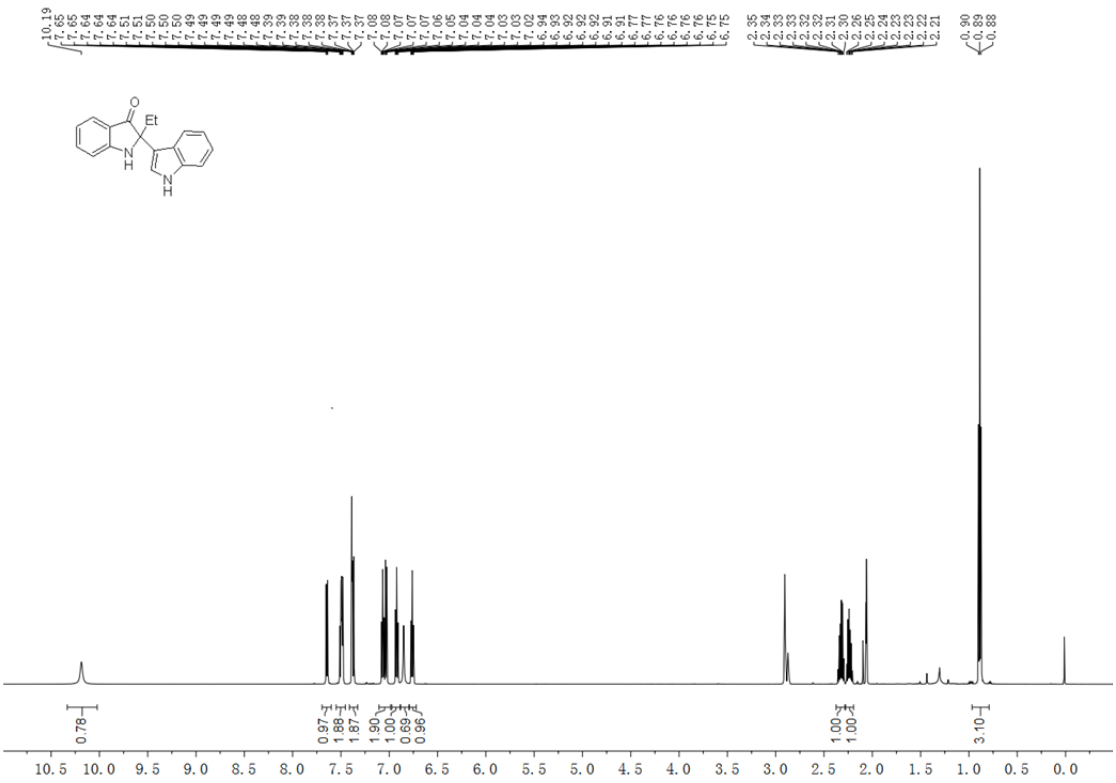

^{13}C NMR spectrum of compound 5i in acetone-d_6 (151 MHz)

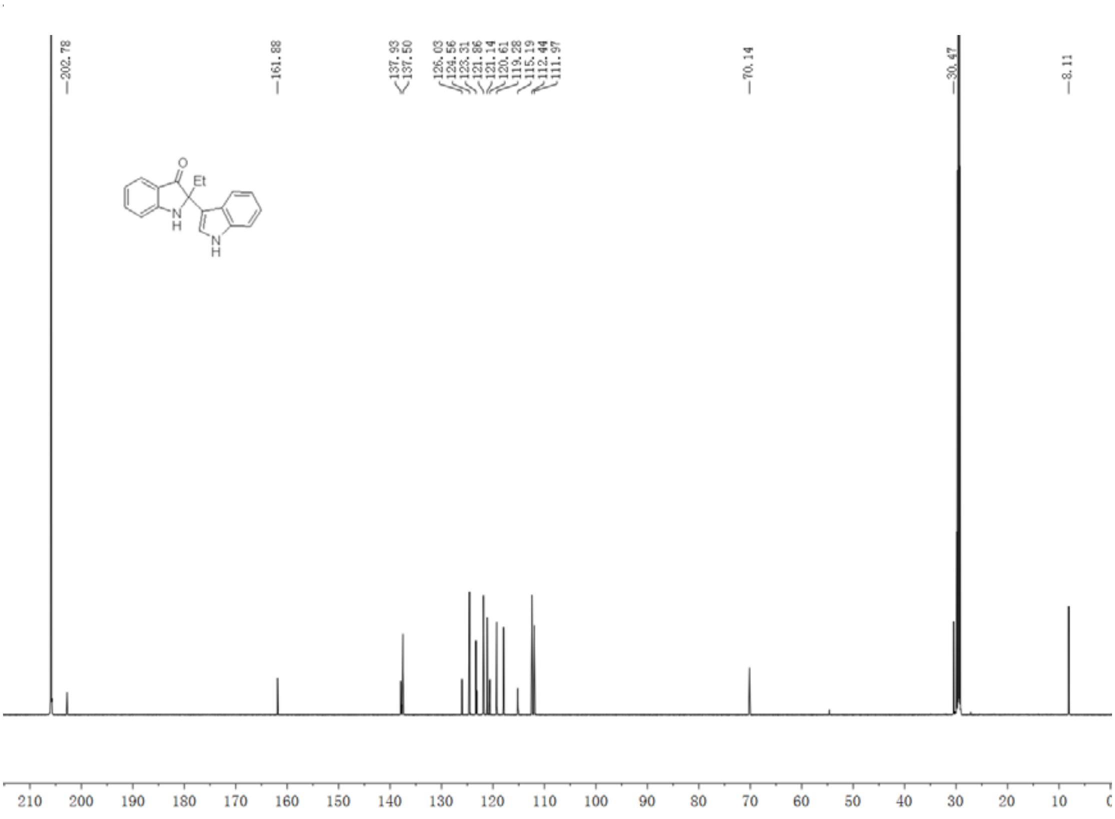

^{1}H NMR spectrum of compound 5j in $CDCl_3$ (600 MHz)

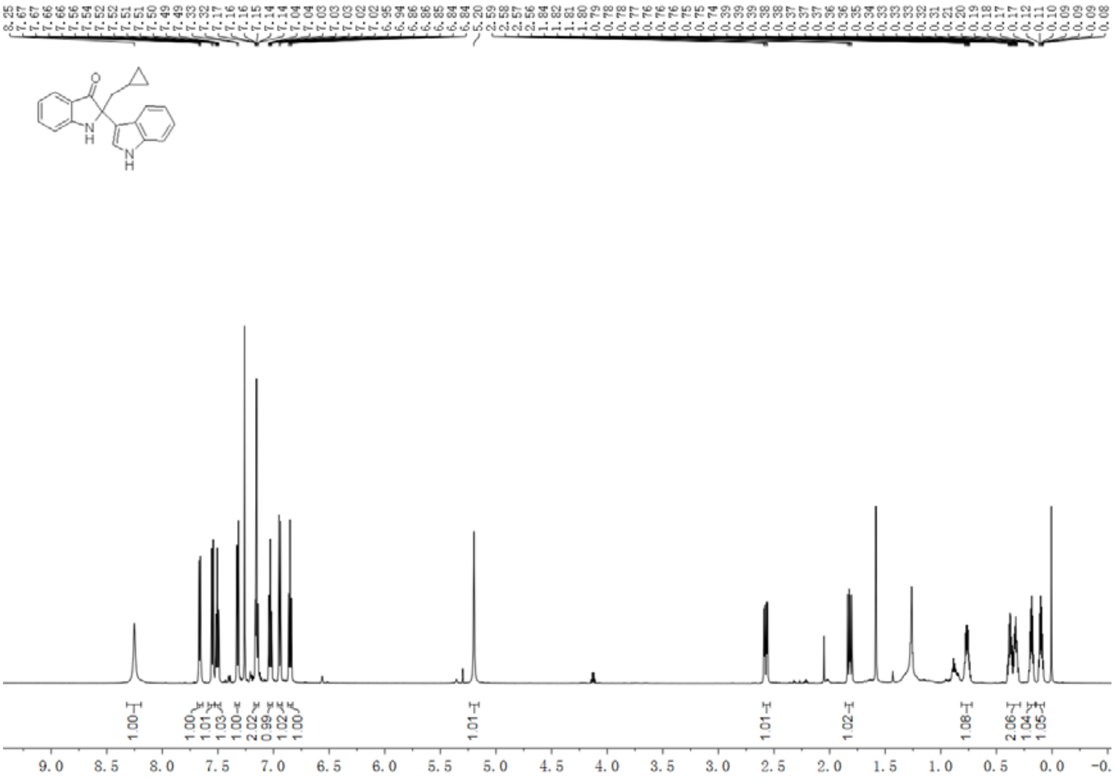

^{13}C NMR spectrum of compound 5j in $CDCl_3$ (151 MHz)

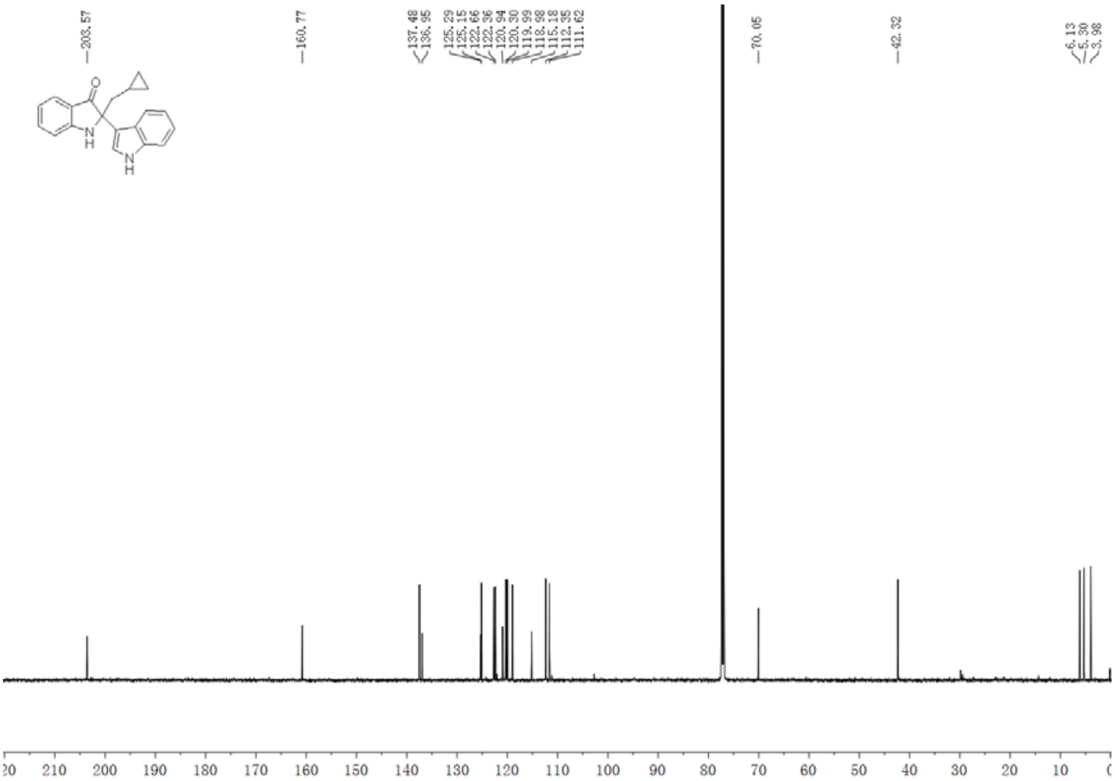

^{1}H NMR spectrum of compound 5k in $CDCl_3$ (600 MHz)

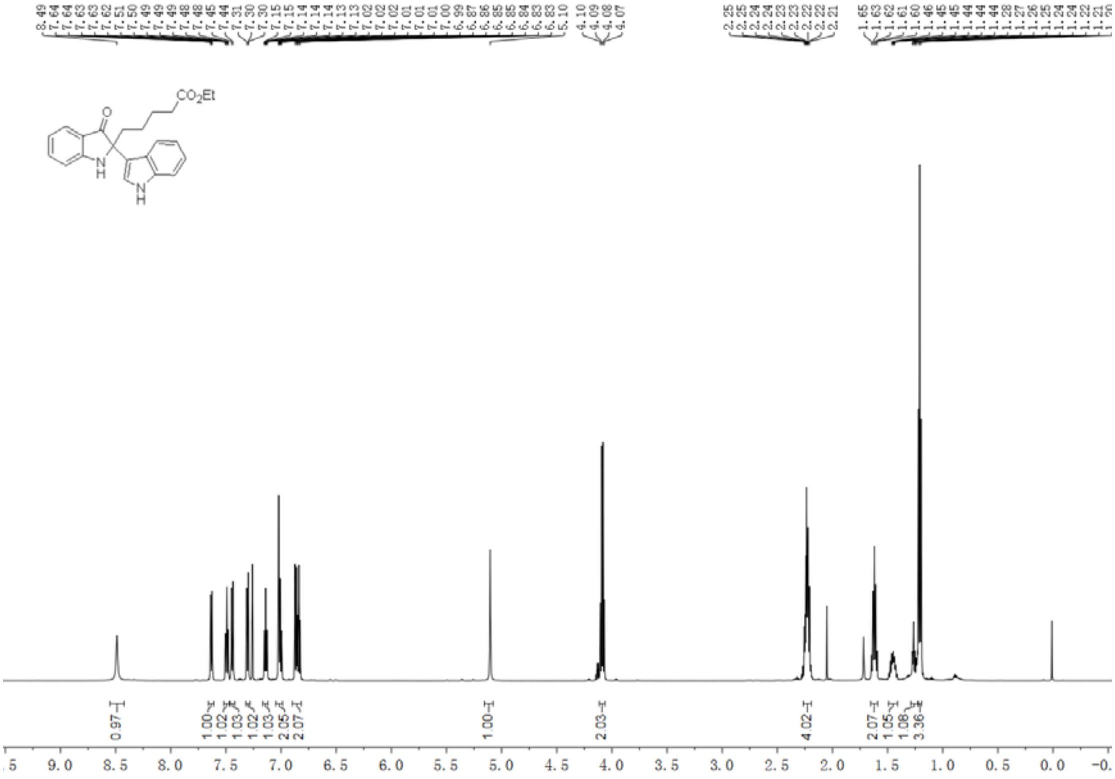

^{13}C NMR spectrum of compound 5k in $CDCl_3$ (151 MHz)

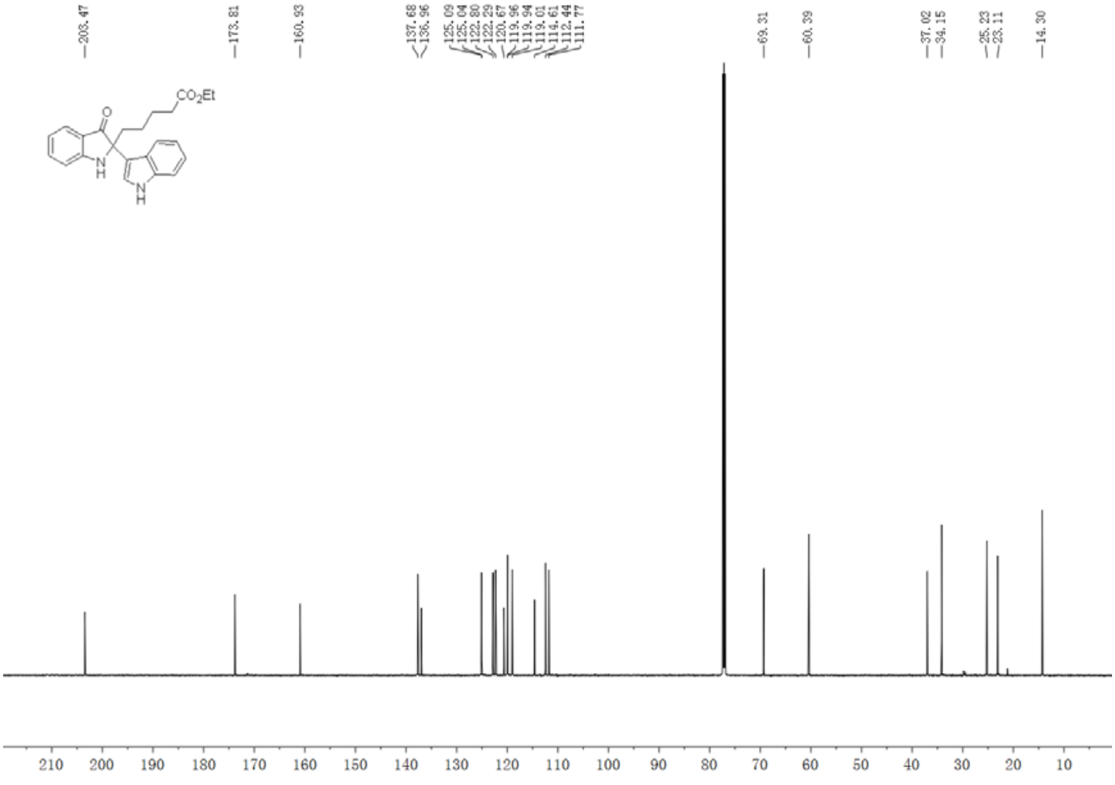

^{1}H NMR spectrum of compound 5l in $CDCl_3$ (600 MHz)

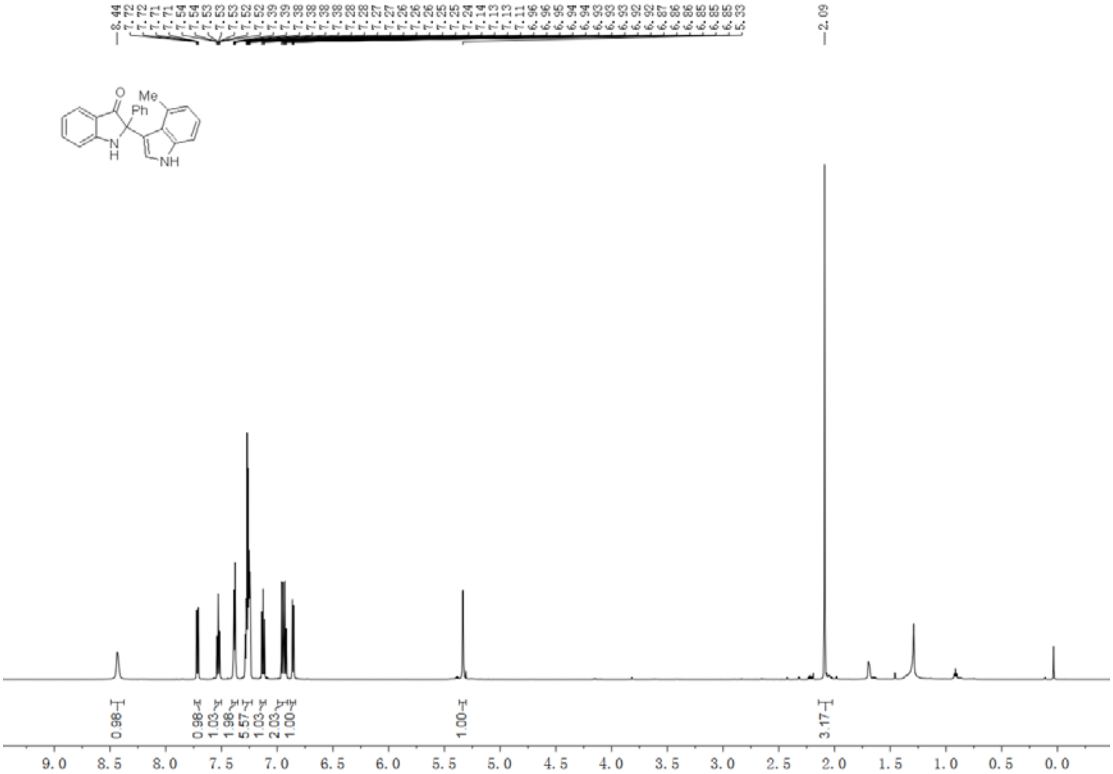

^{13}C NMR spectrum of compound 5l in $CDCl_3$ (151 MHz)

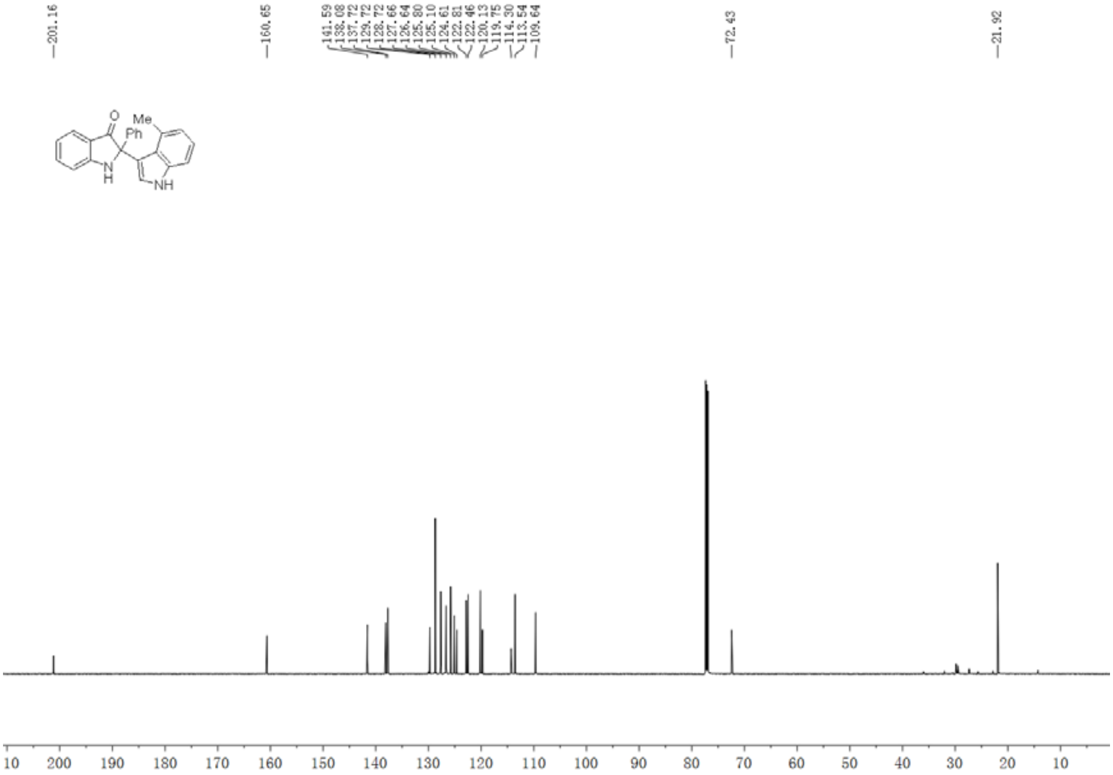

^{1}H NMR spectrum of compound 5m in $CDCl_3$ (600 MHz)

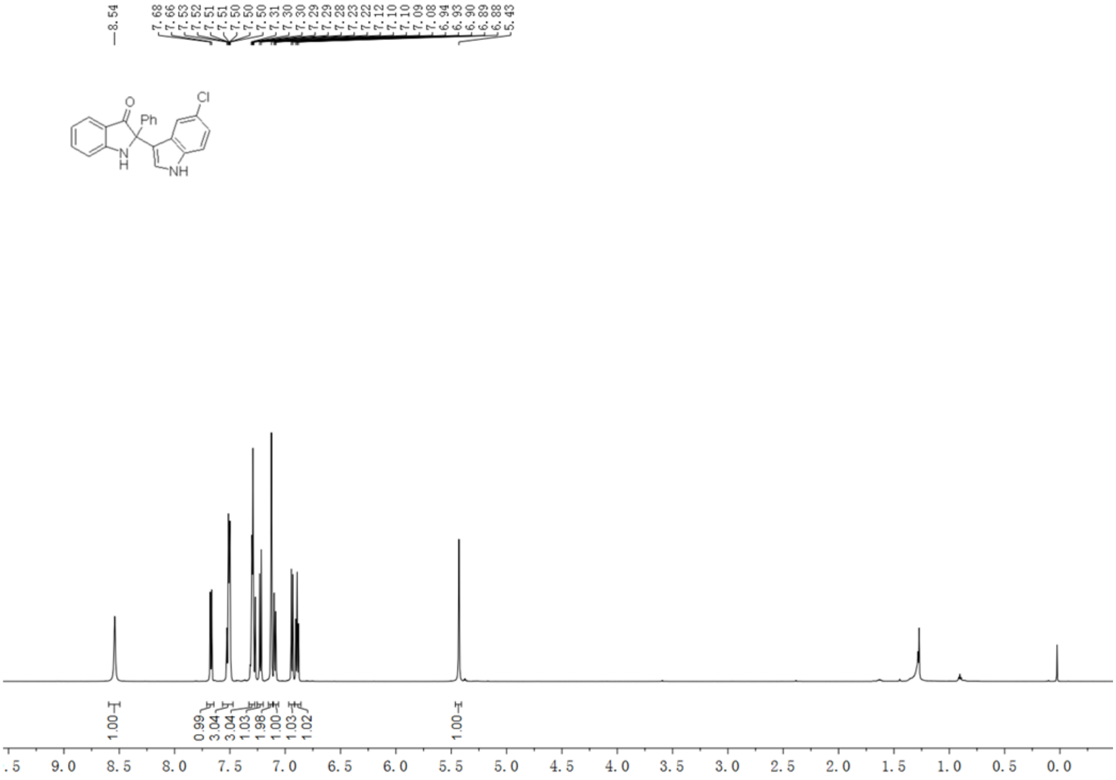

^{13}C NMR spectrum of compound 5m in $CDCl_3$ (151 MHz)

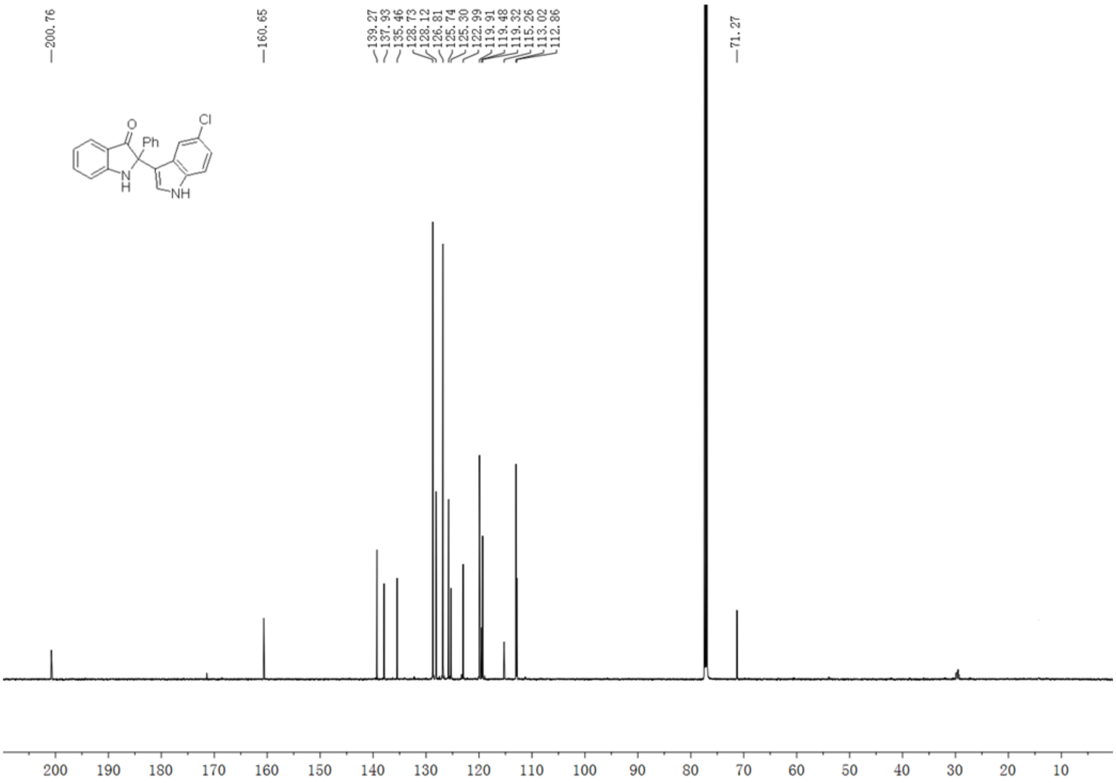

^{1}H NMR spectrum of compound 5n in $CDCl_3$ (600 MHz)

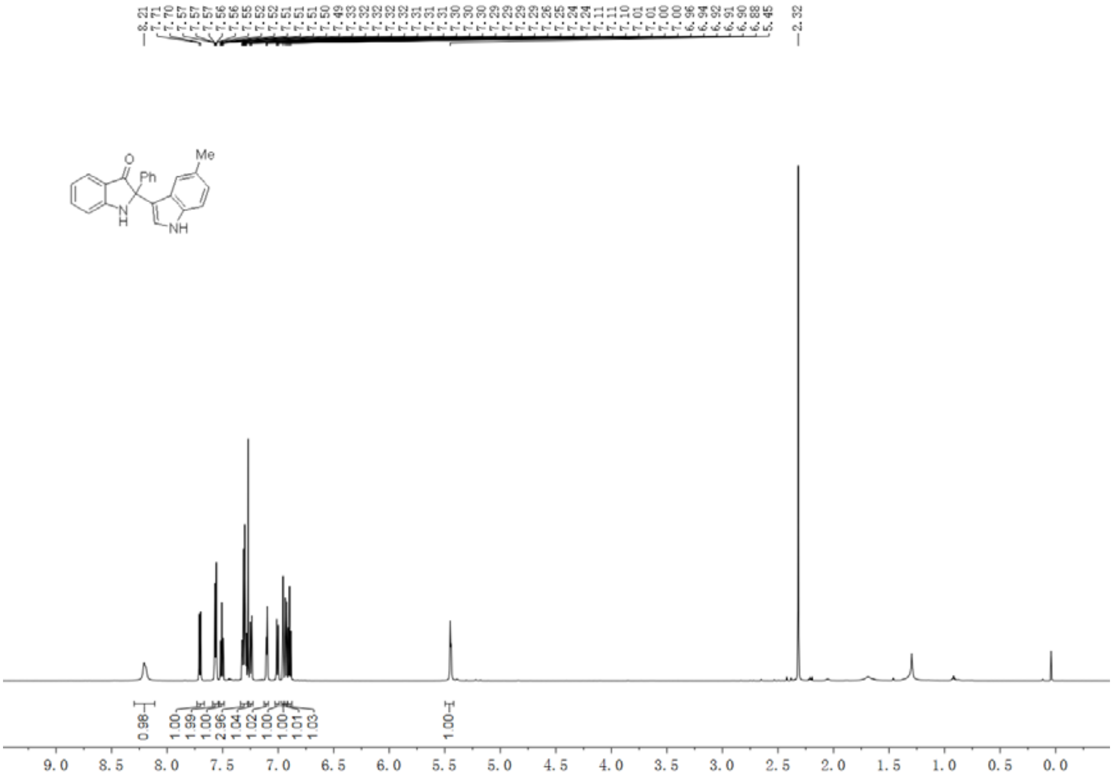

^{13}C NMR spectrum of compound 5n in $CDCl_3$ (151 MHz)

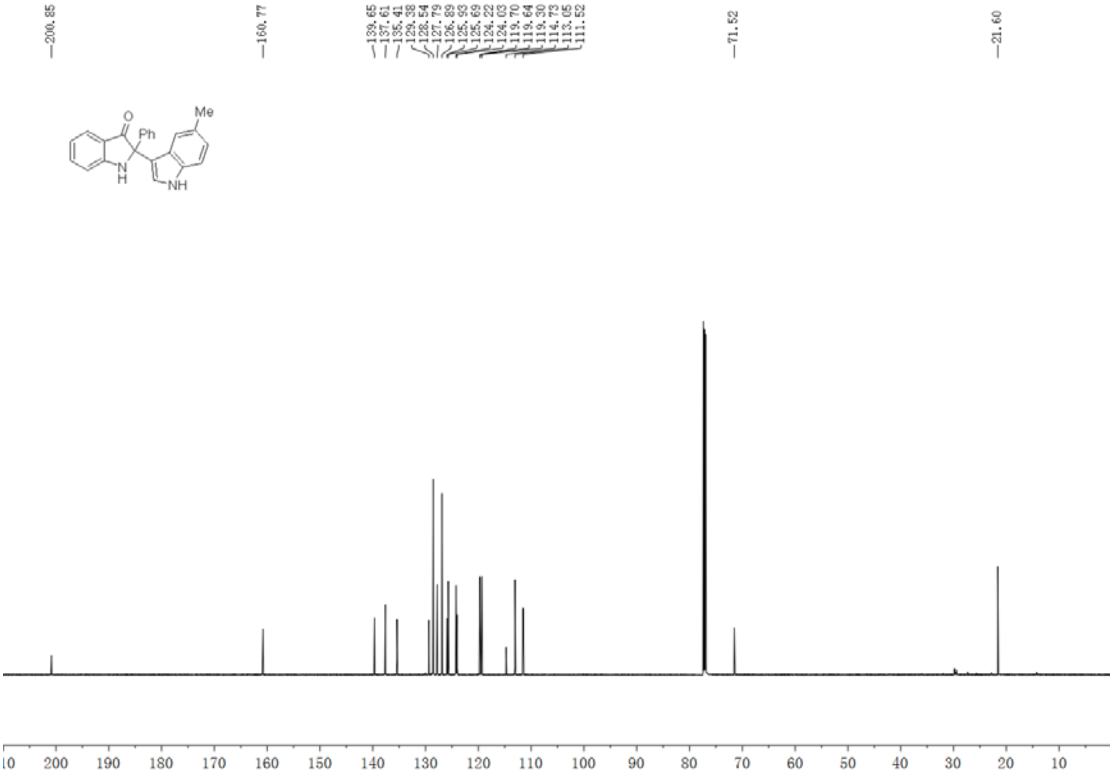

^{1}H NMR spectrum of compound 5o in $CDCl_3$ (600 MHz)

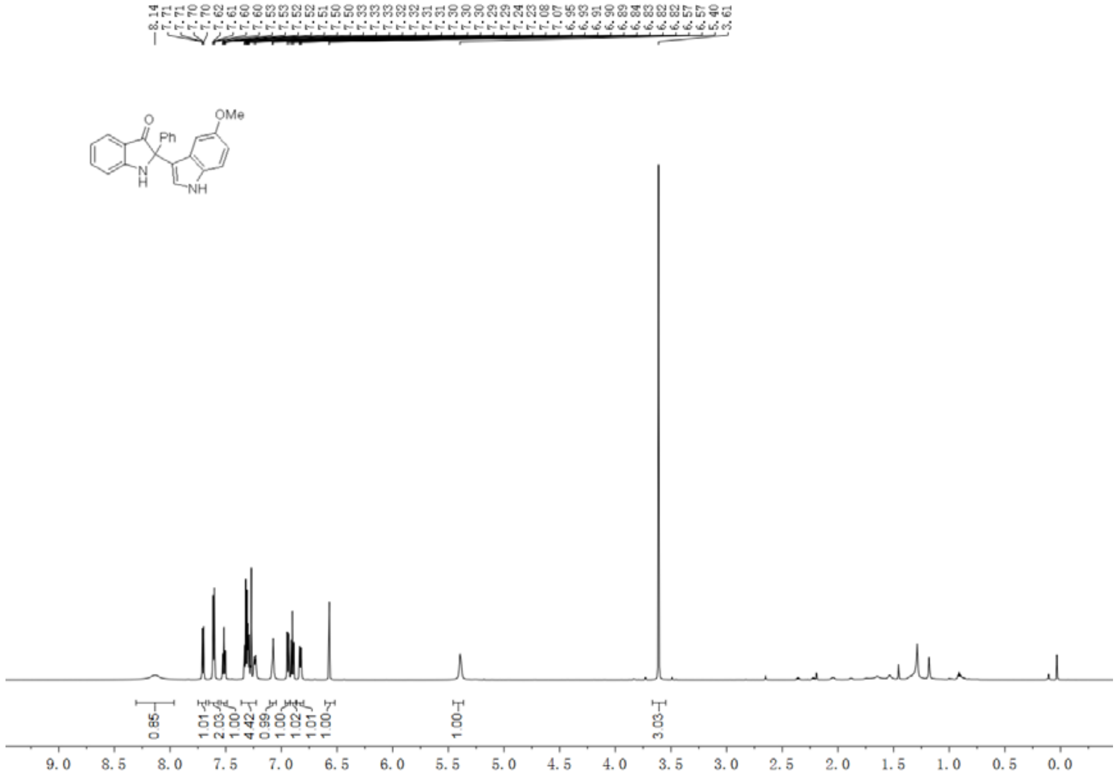

^{13}C NMR spectrum of compound 5o in $CDCl_3$ (151 MHz)

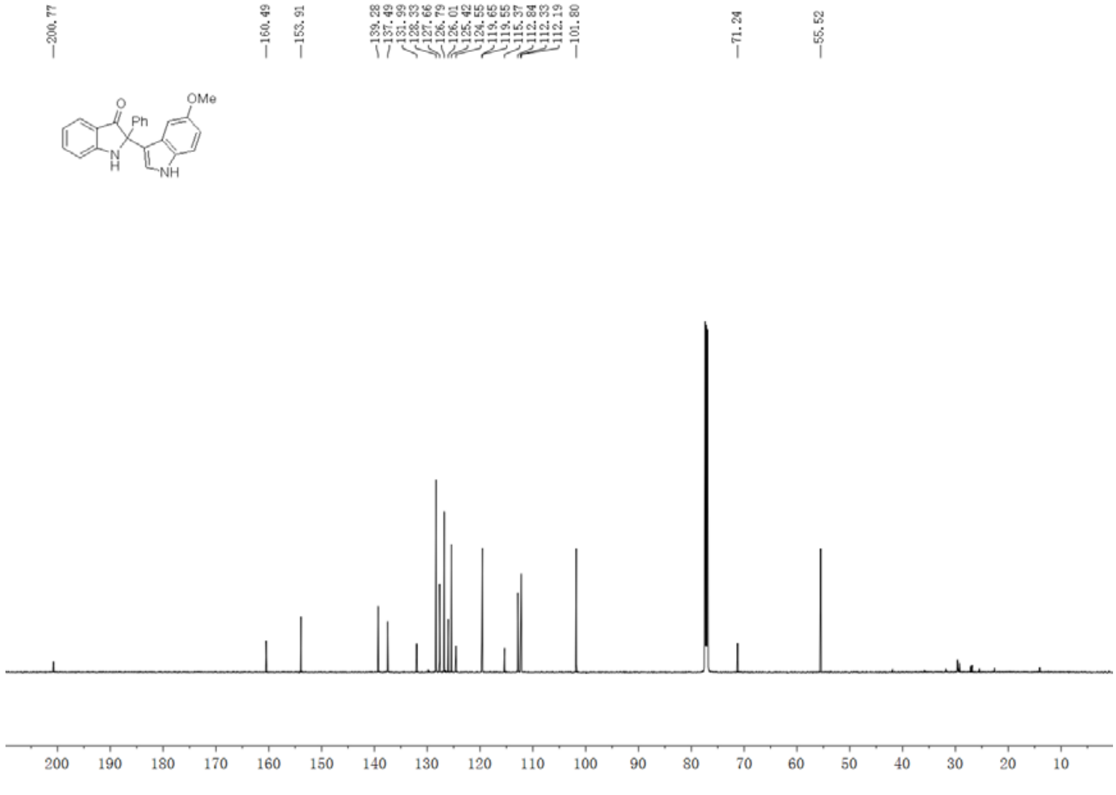

^{1}H NMR spectrum of compound 5p in $CDCl_3$ (600 MHz)

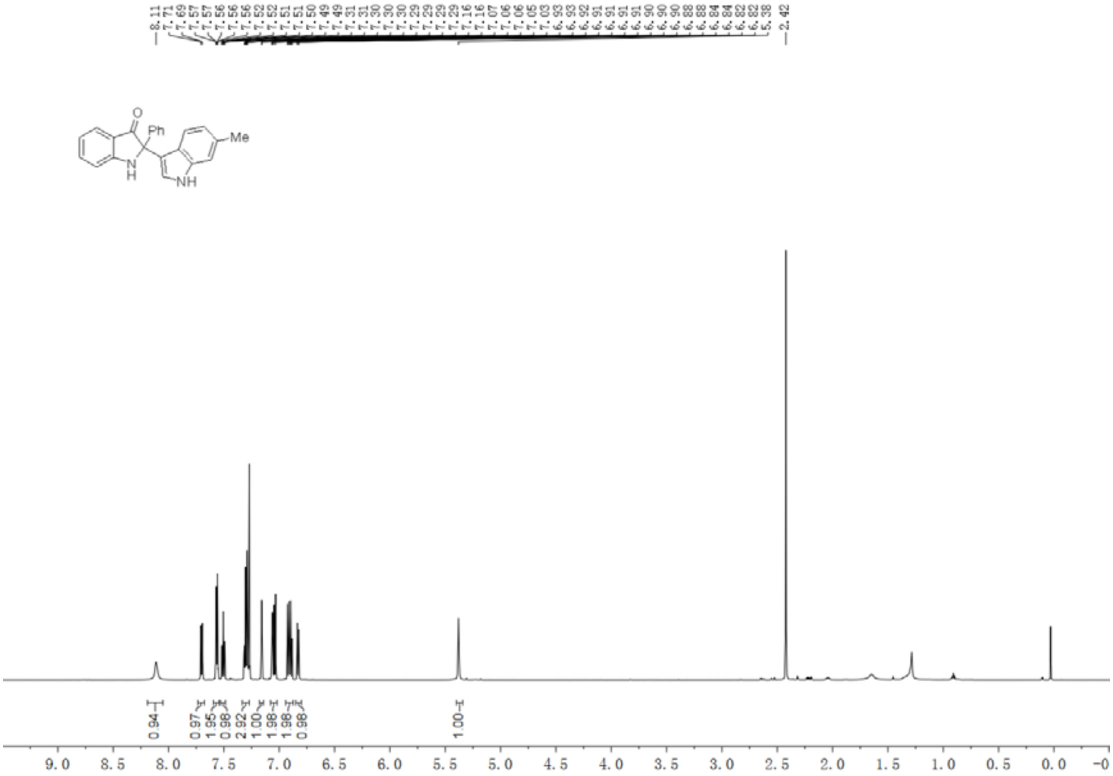

^{13}C NMR spectrum of compound 5p in $CDCl_3$ (151 MHz)

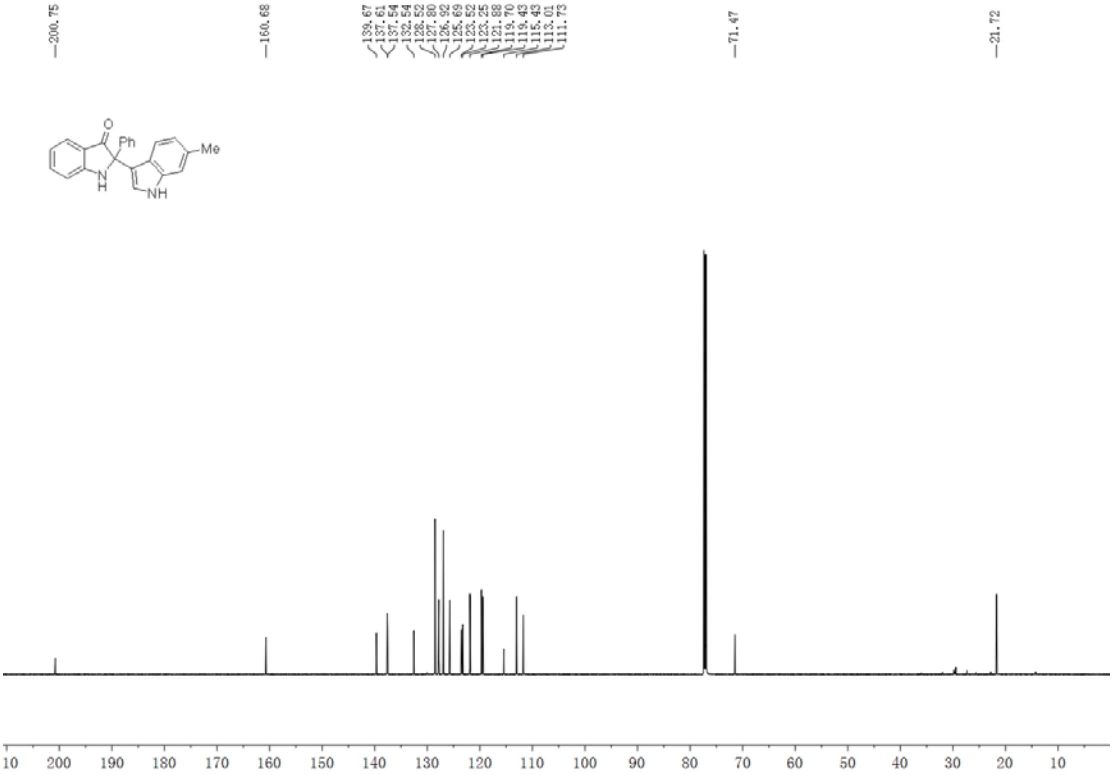

^{1}H NMR spectrum of compound 5q in $CDCl_3$ (600 MHz)

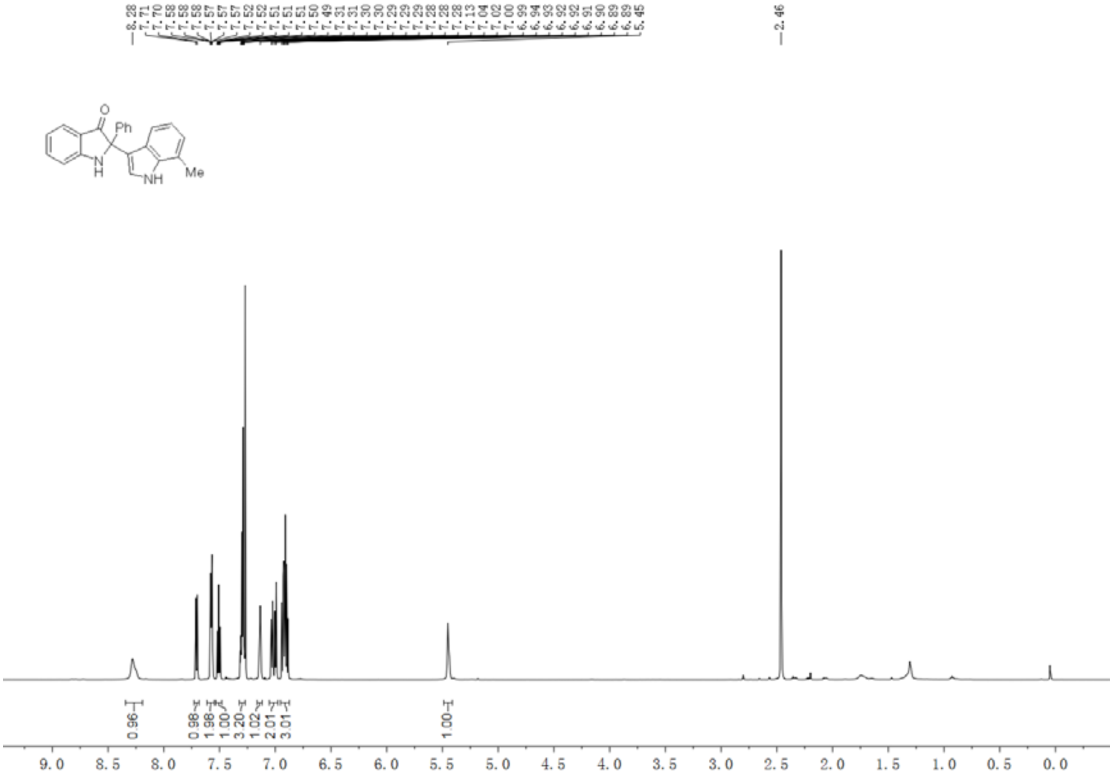

^{13}C NMR spectrum of compound 5q in $CDCl_3$ (151 MHz)

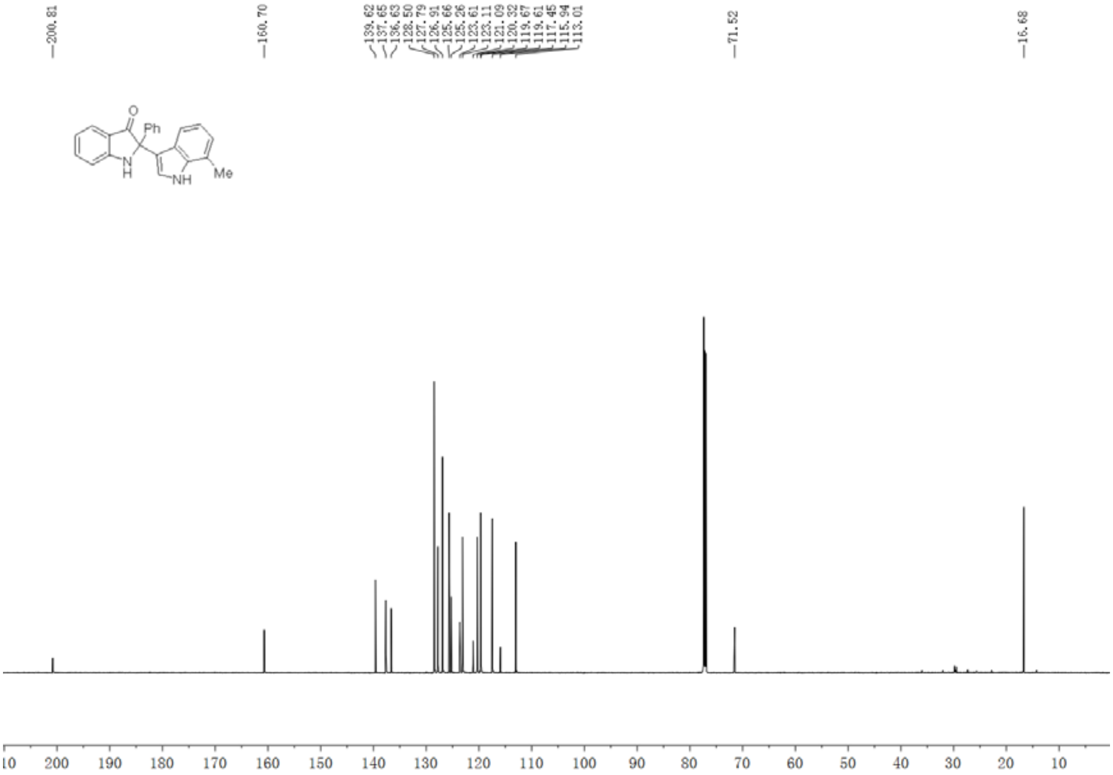

^{1}H NMR spectrum of compound 5r in $CDCl_3$ (600 MHz)

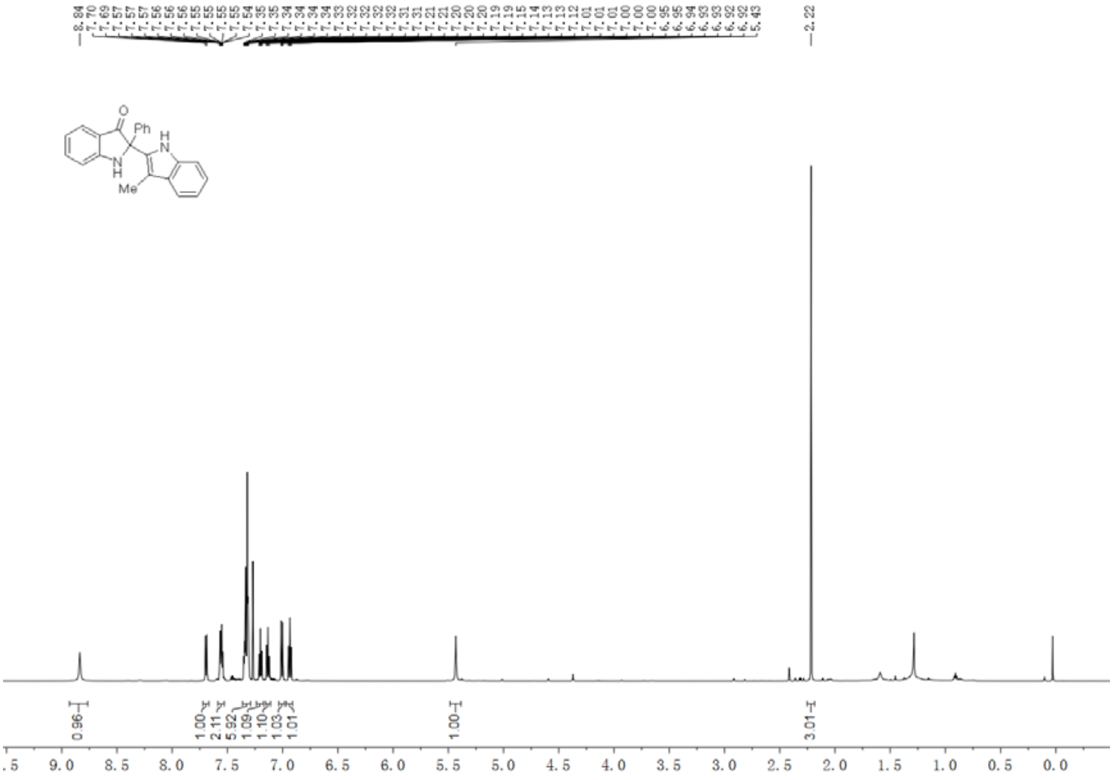

^{13}C NMR spectrum of compound 5r in $CDCl_3$ (151 MHz)

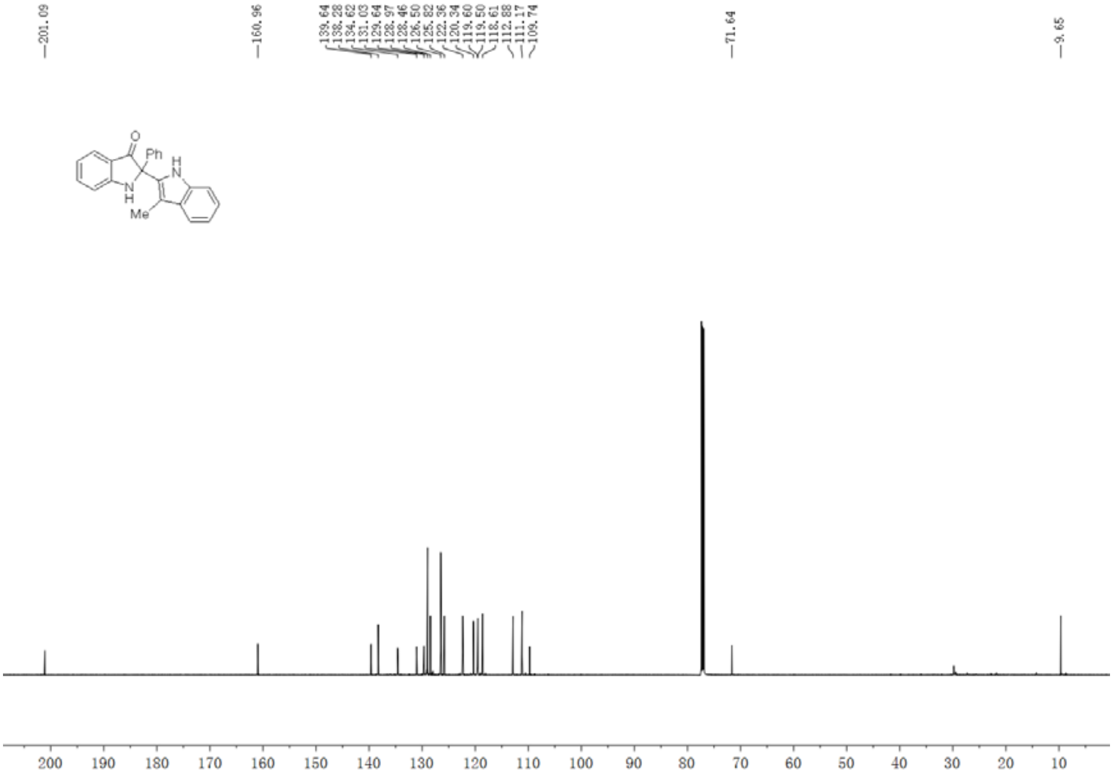

^{1}H NMR spectrum of compound 5s in $CDCl_3$ (600 MHz)

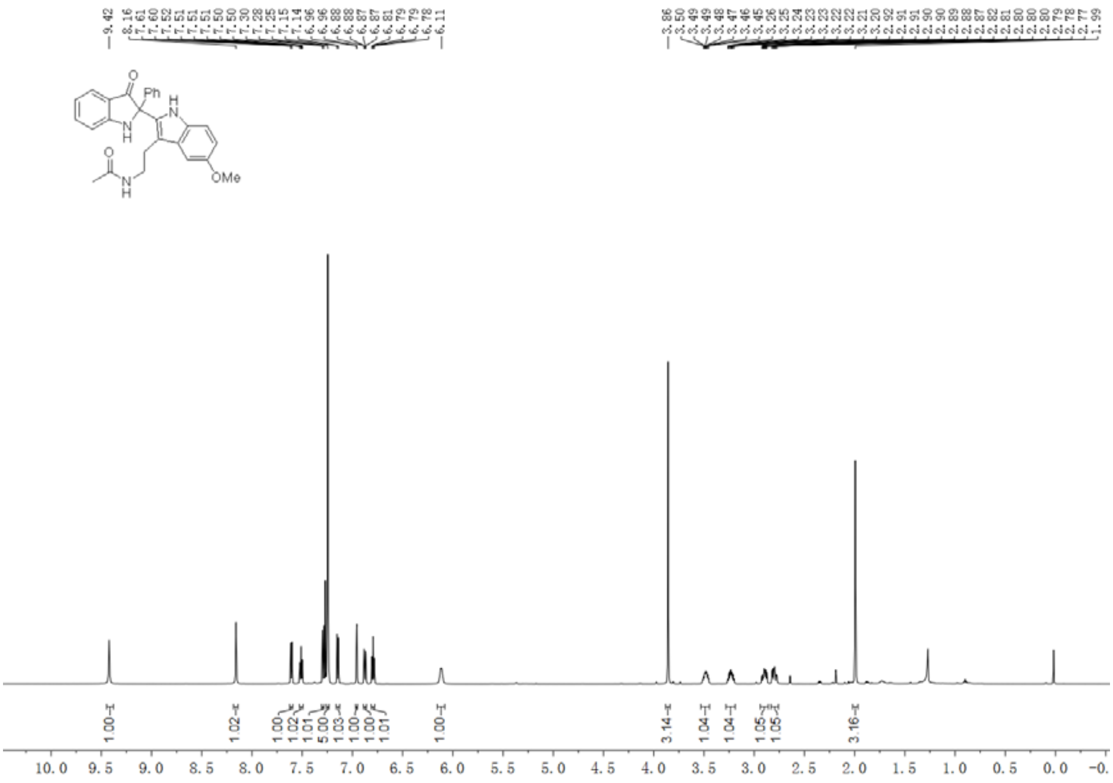

^{13}C NMR spectrum of compound 5s in $CDCl_3$ (151 MHz)

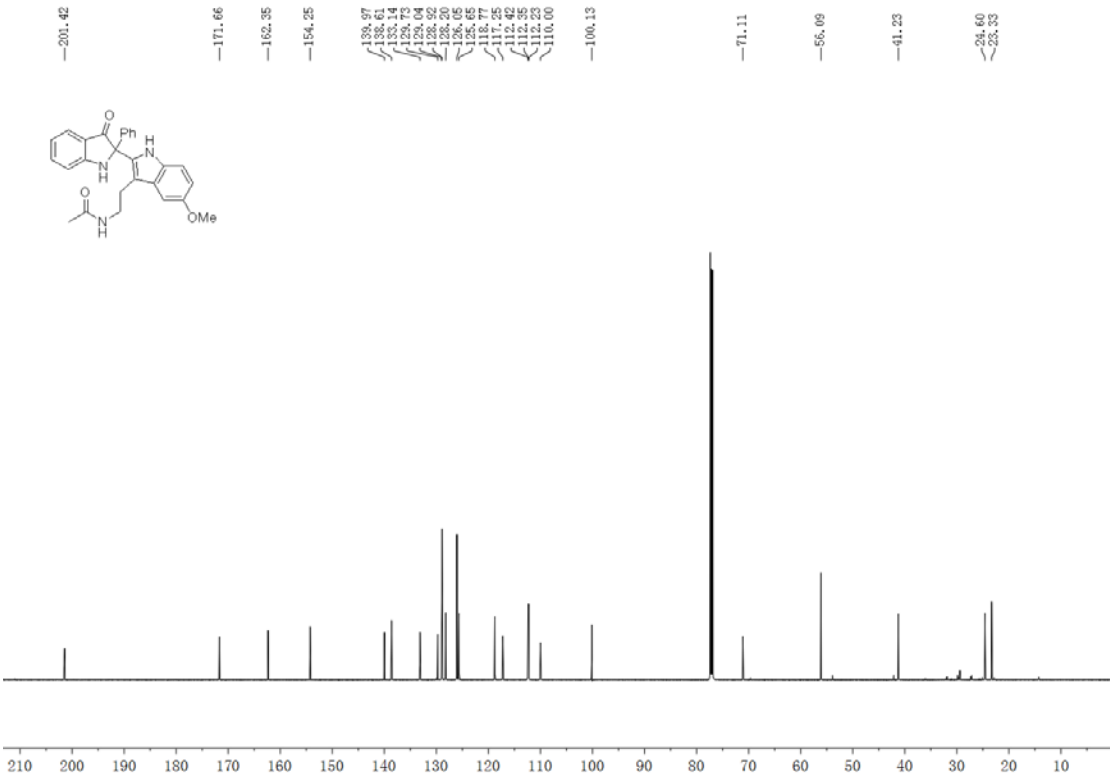

^{1}H NMR spectrum of compound 5t in $CDCl_3$ (600 MHz)

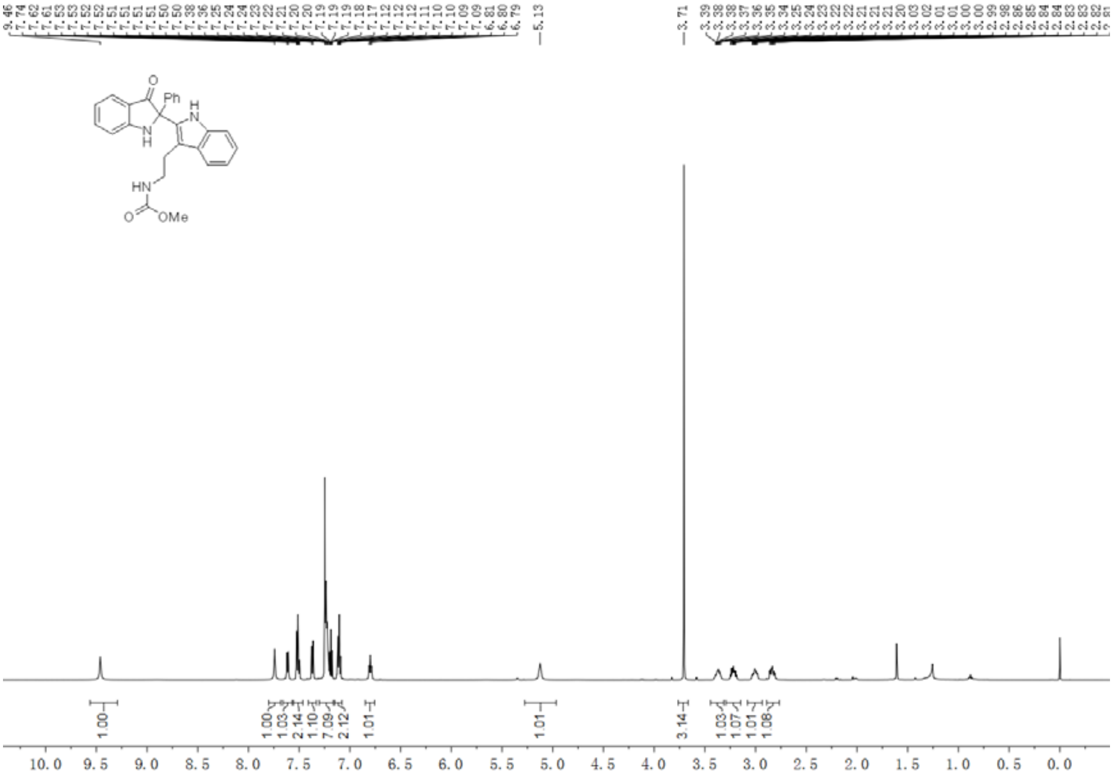

^{13}C NMR spectrum of compound 5t in $CDCl_3$ (151 MHz)

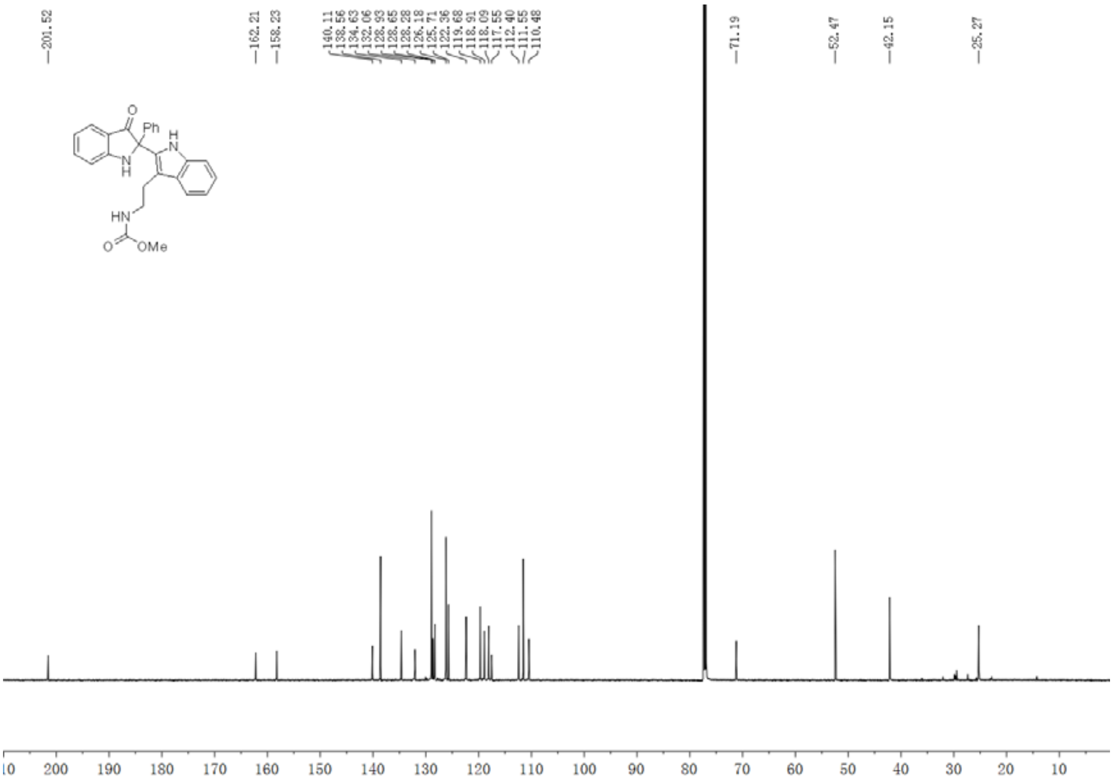

^{1}H NMR spectrum of compound 6a in acetone-d_6 (600 MHz)

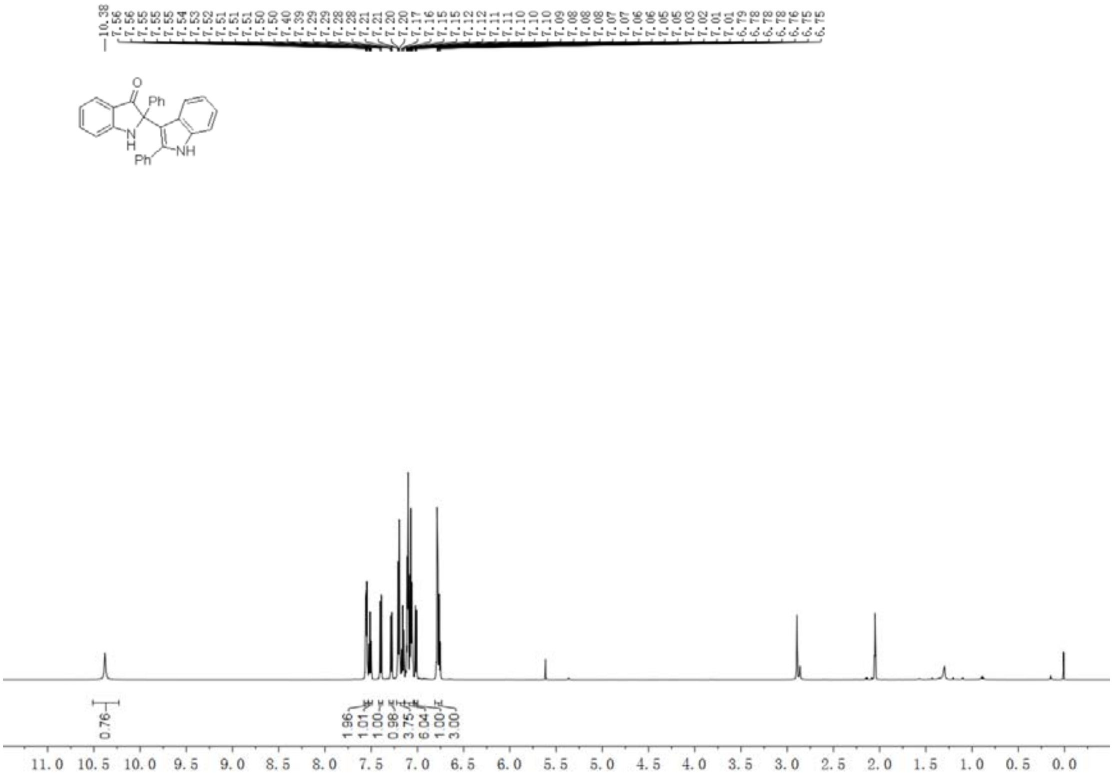

^{13}C NMR spectrum of compound 6a in acetone-d_6 (151 MHz)

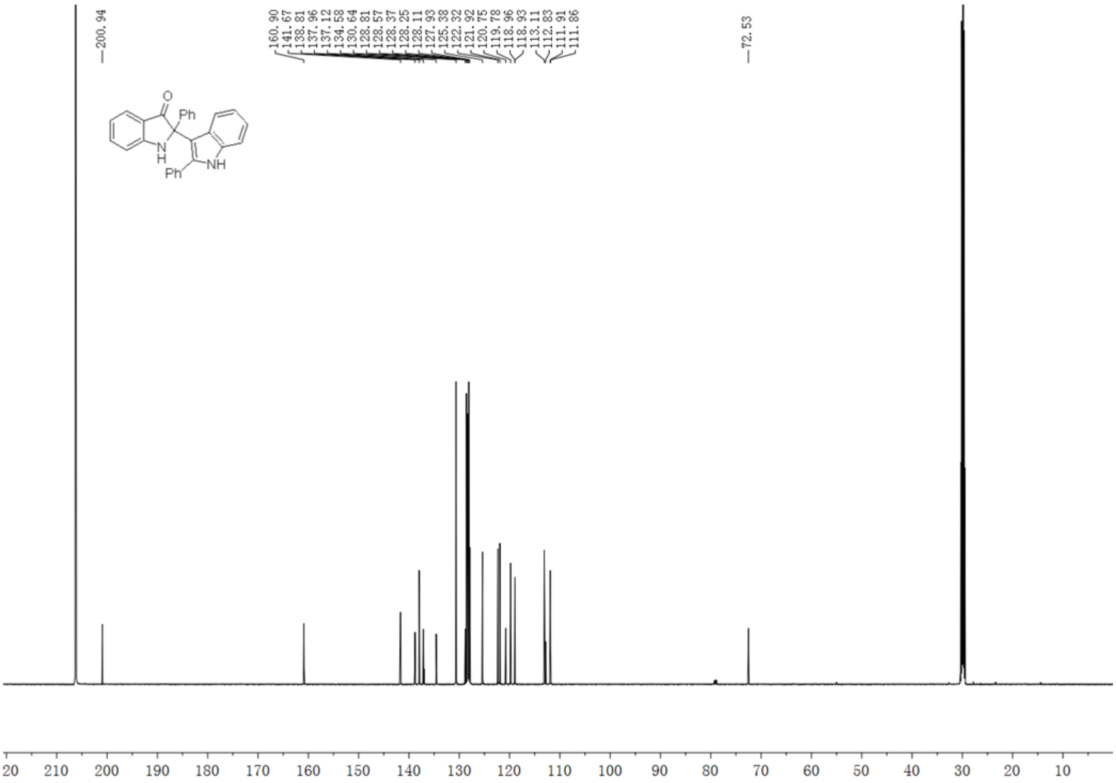

^{1}H NMR spectrum of compound 6b in $CDCl_3$ (600 MHz)

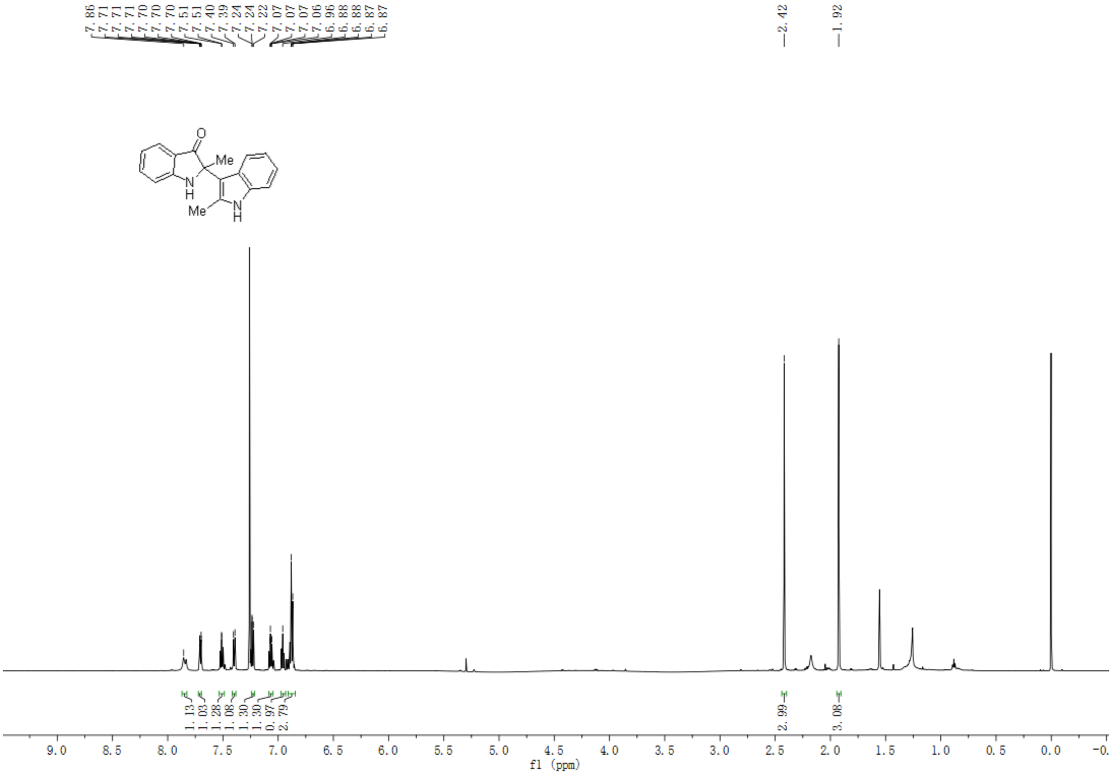

^{13}C NMR spectrum of compound 6b in $CDCl_3$ (151 MHz)

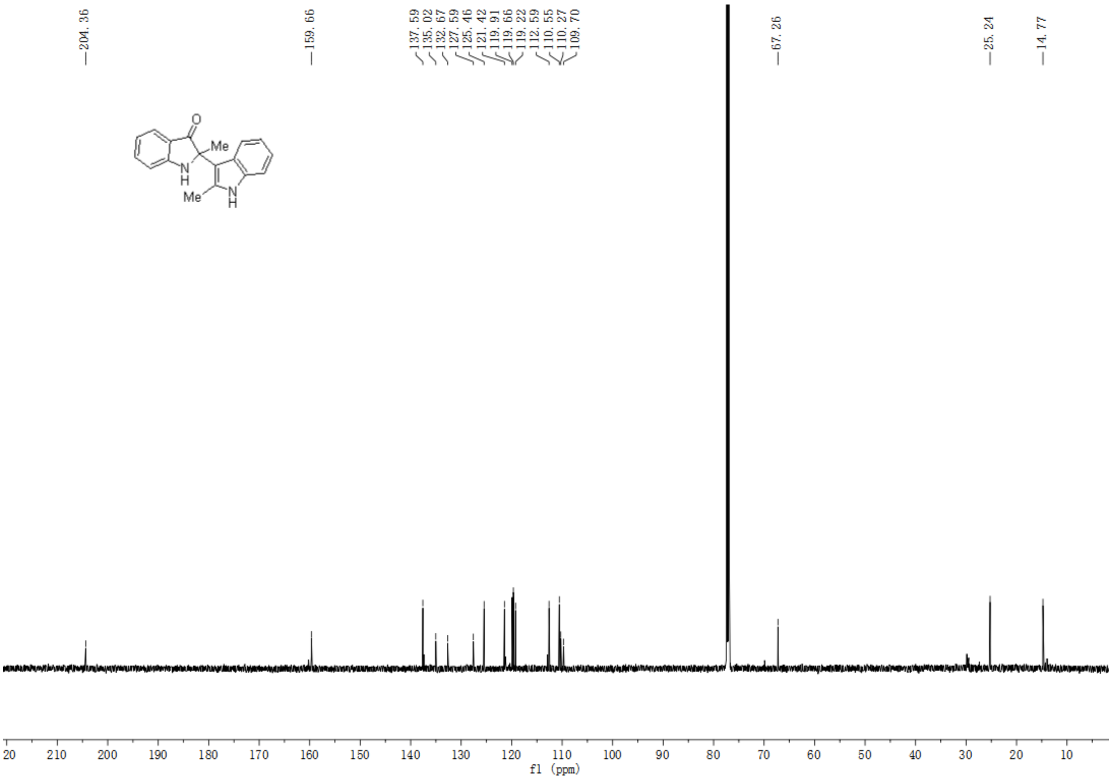

^{1}H NMR spectrum of compound 6c in DMSO-d_6 (600 MHz)

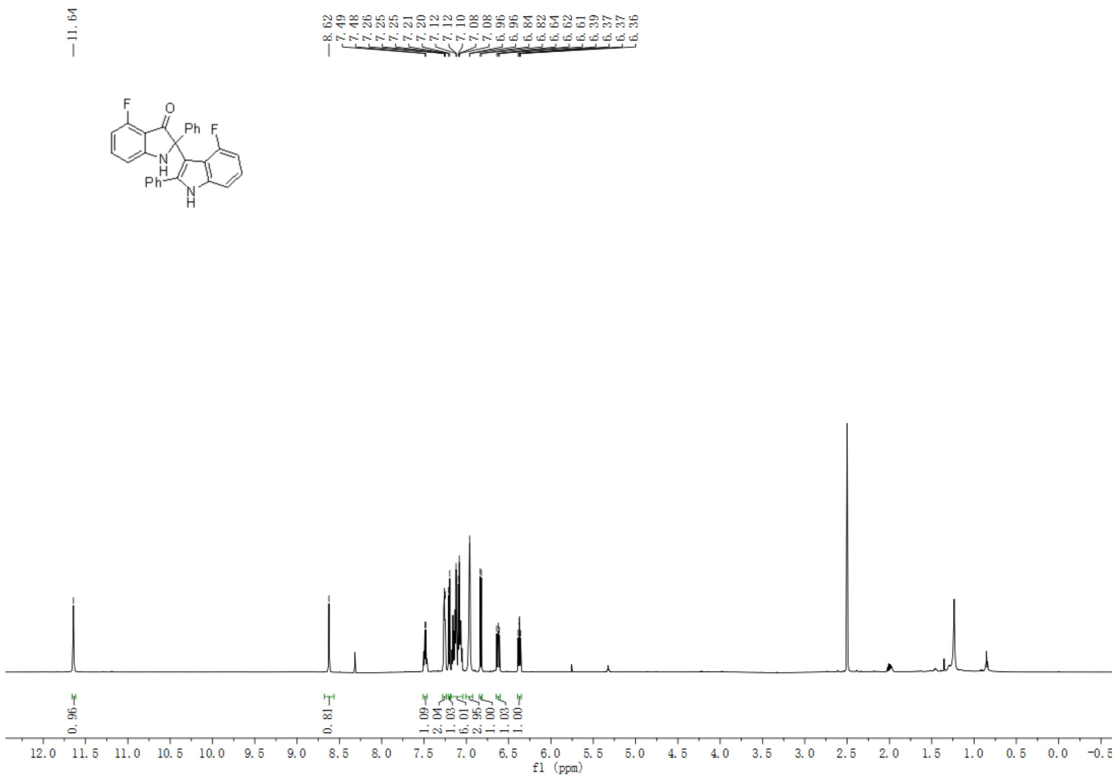

^{13}C NMR spectrum of compound 6c in DMSO-d_6 (151 MHz)

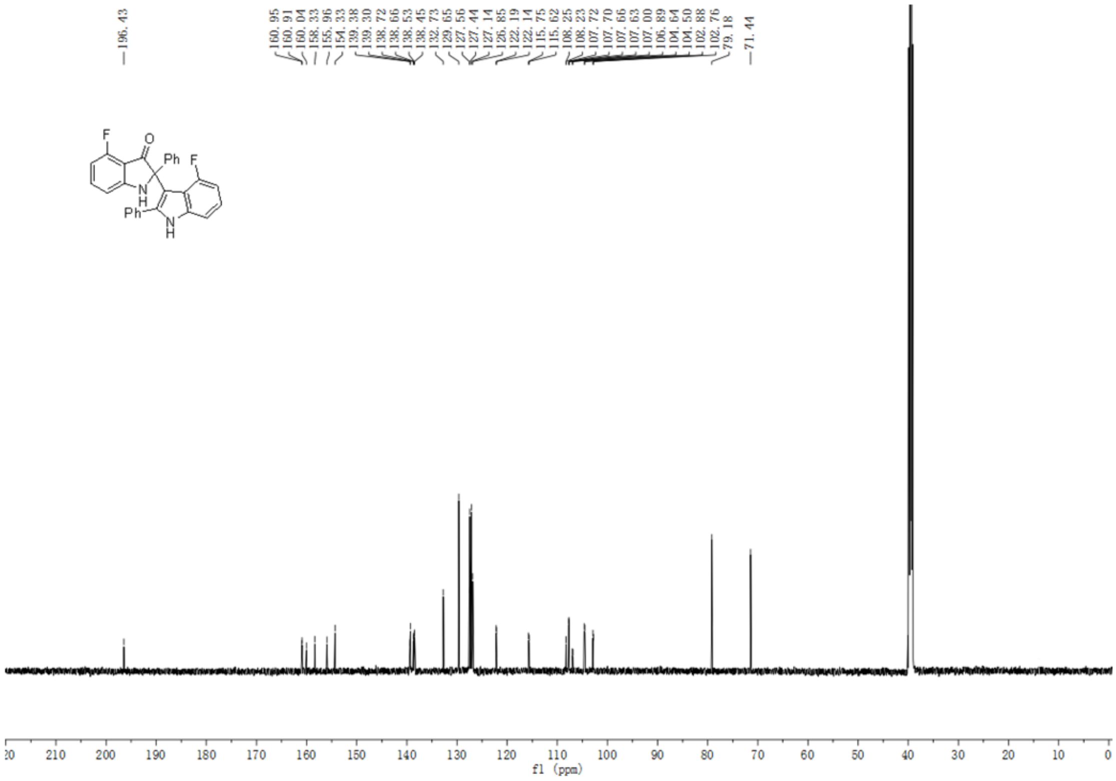

^{1}H NMR spectrum of compound 6d in $CDCl_3$ (600 MHz)

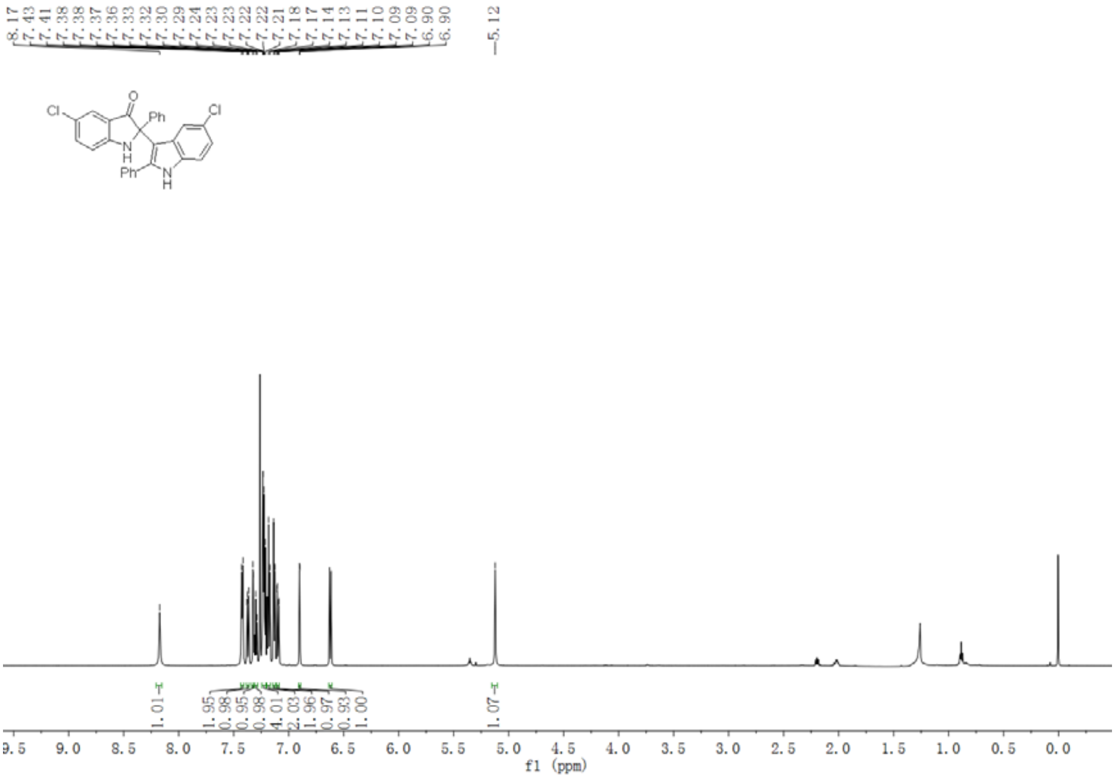

^{13}C NMR spectrum of compound 6d in $CDCl_3$ (151 MHz)

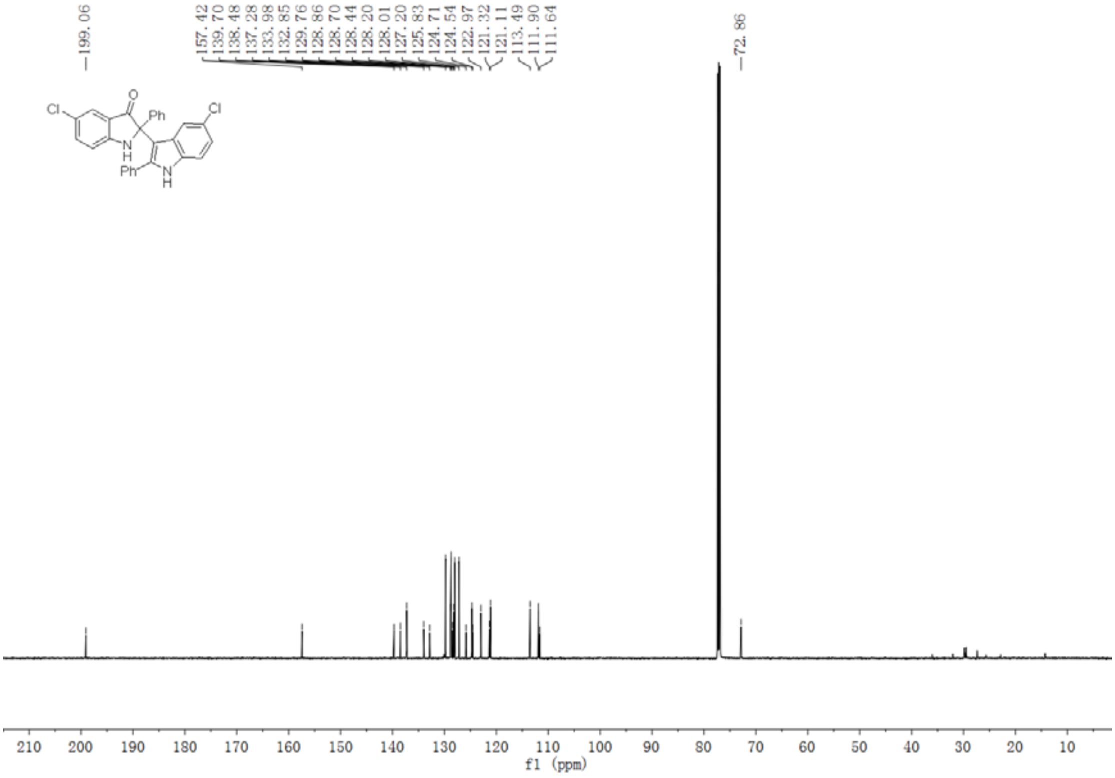

^{1}H NMR spectrum of compound 6e in $CDCl_3$ (600 MHz)

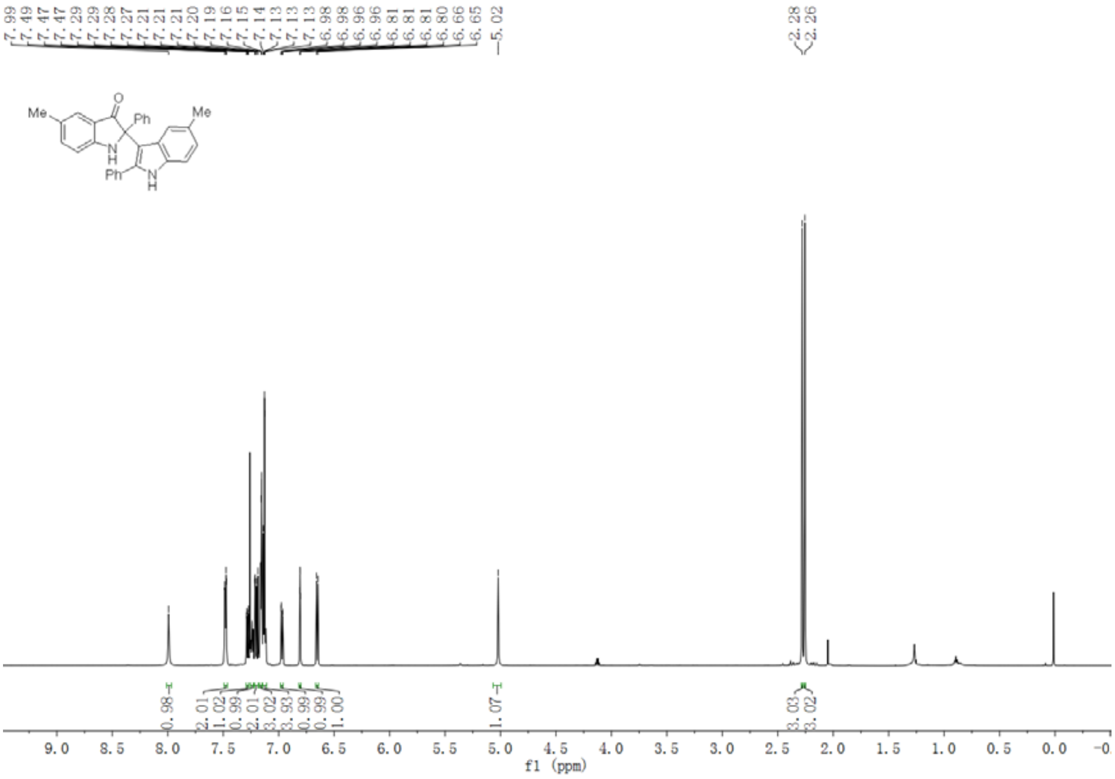

^{13}C NMR spectrum of compound 6e in $CDCl_3$ (151 MHz)

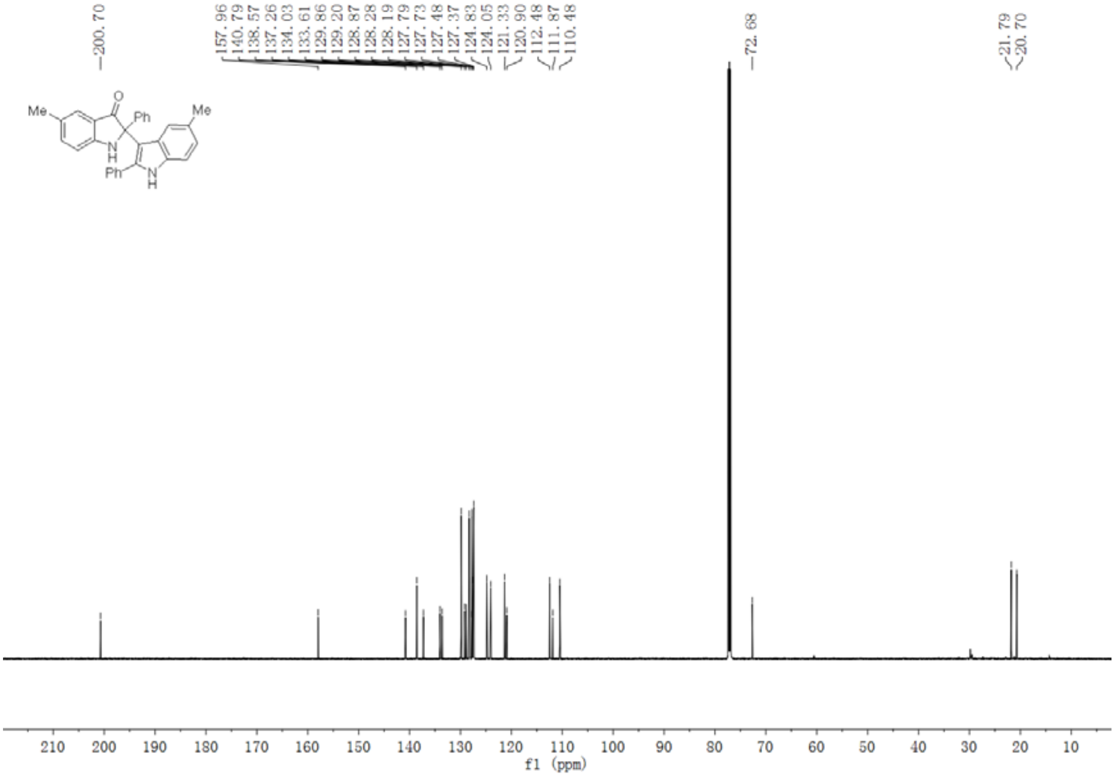

^{1}H NMR spectrum of compound 6f in $CDCl_3$ (600 MHz)

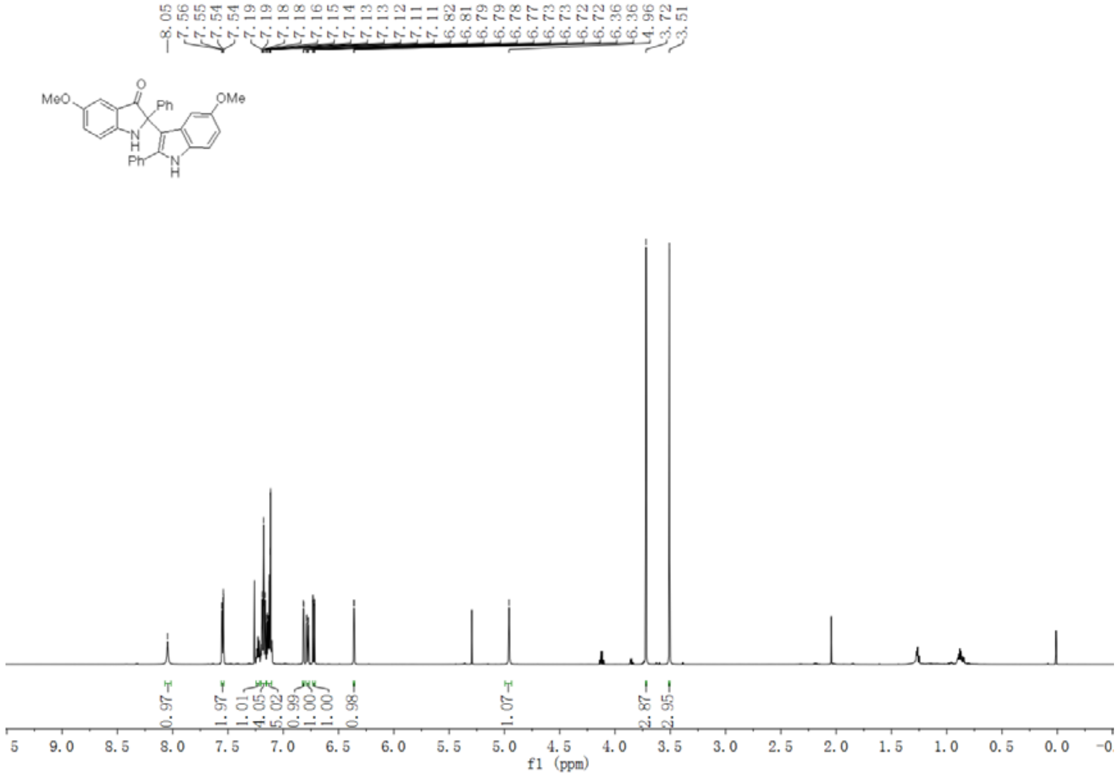

^{13}C NMR spectrum of compound 6f in $CDCl_3$ (151 MHz)

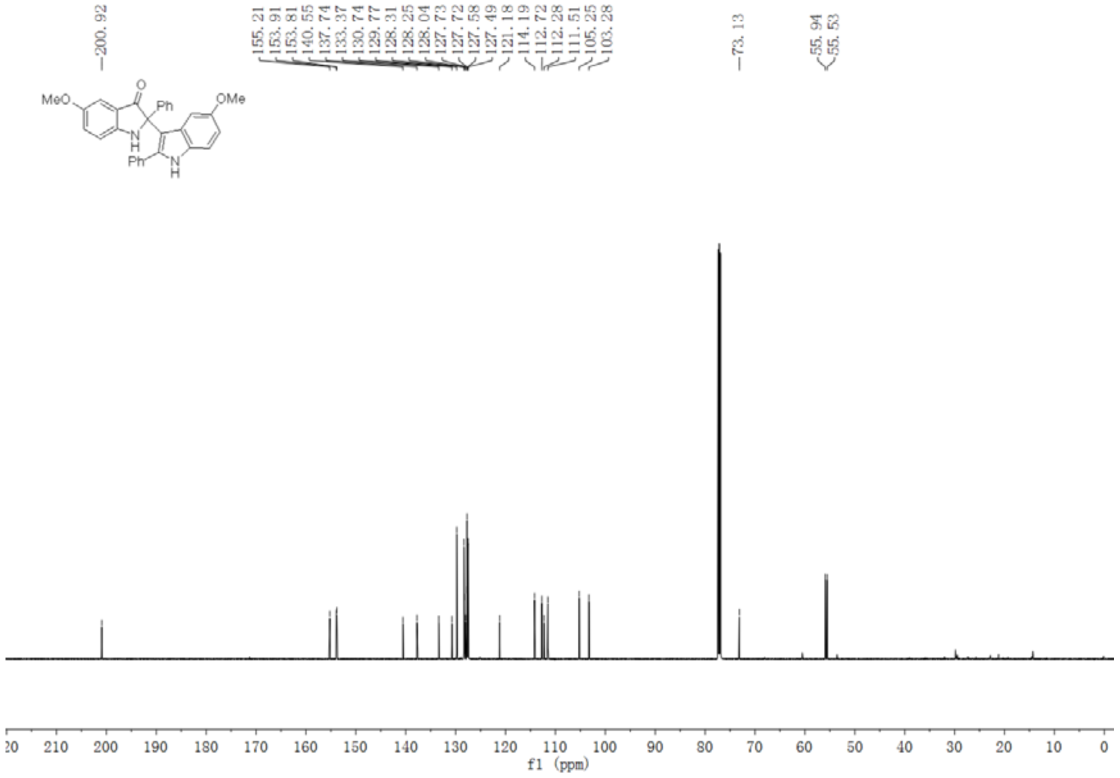

^{1}H NMR spectrum of compound 6g in acetone-d_6 (600 MHz)

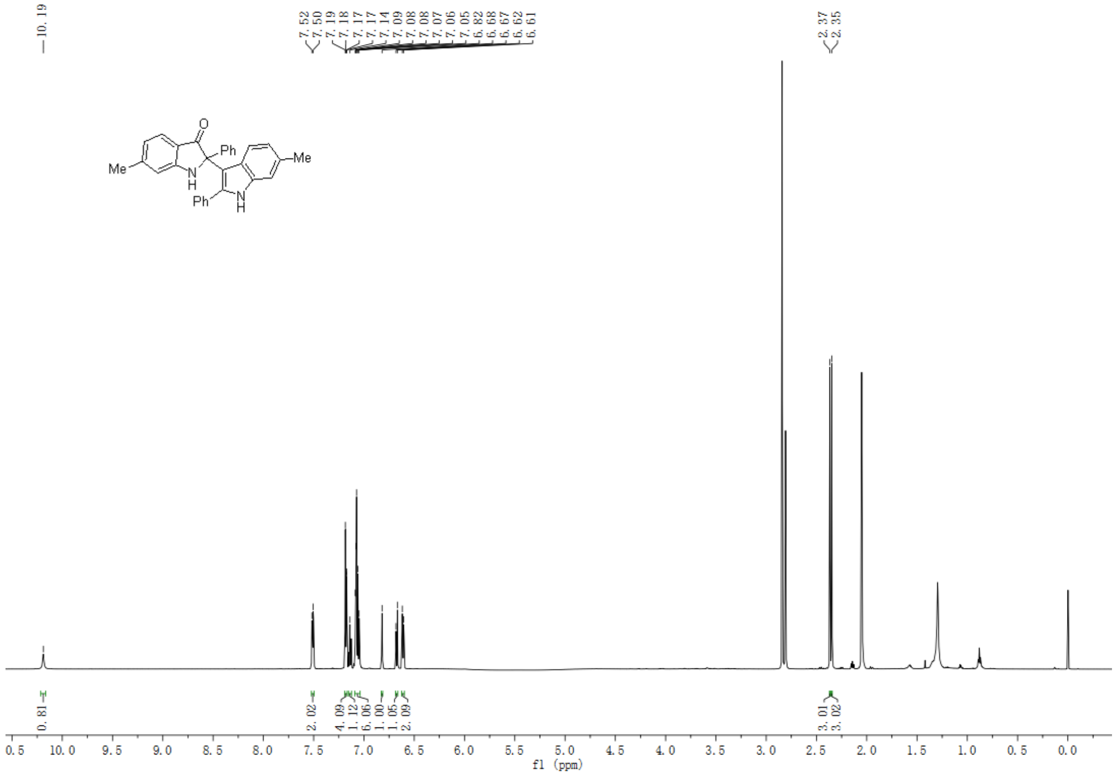

^{13}C NMR spectrum of compound 6g in acetone-d_6 (151 MHz)

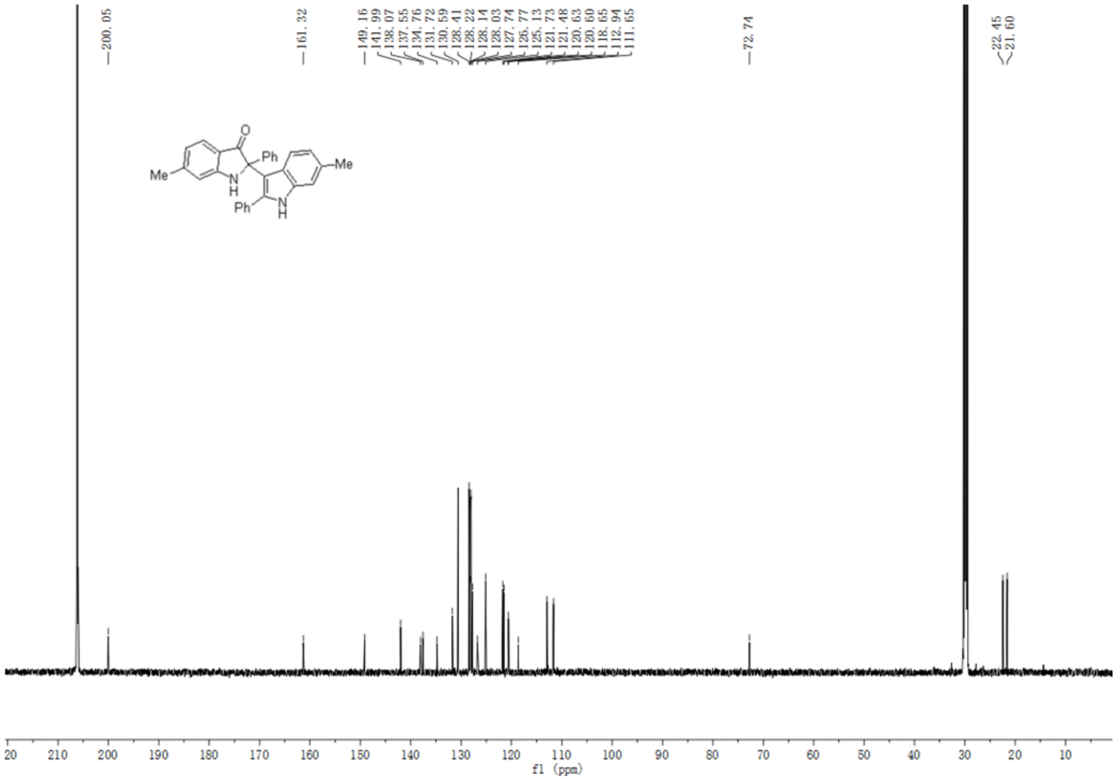

^{1}H NMR spectrum of compound 6h in $CDCl_3$ (600 MHz)

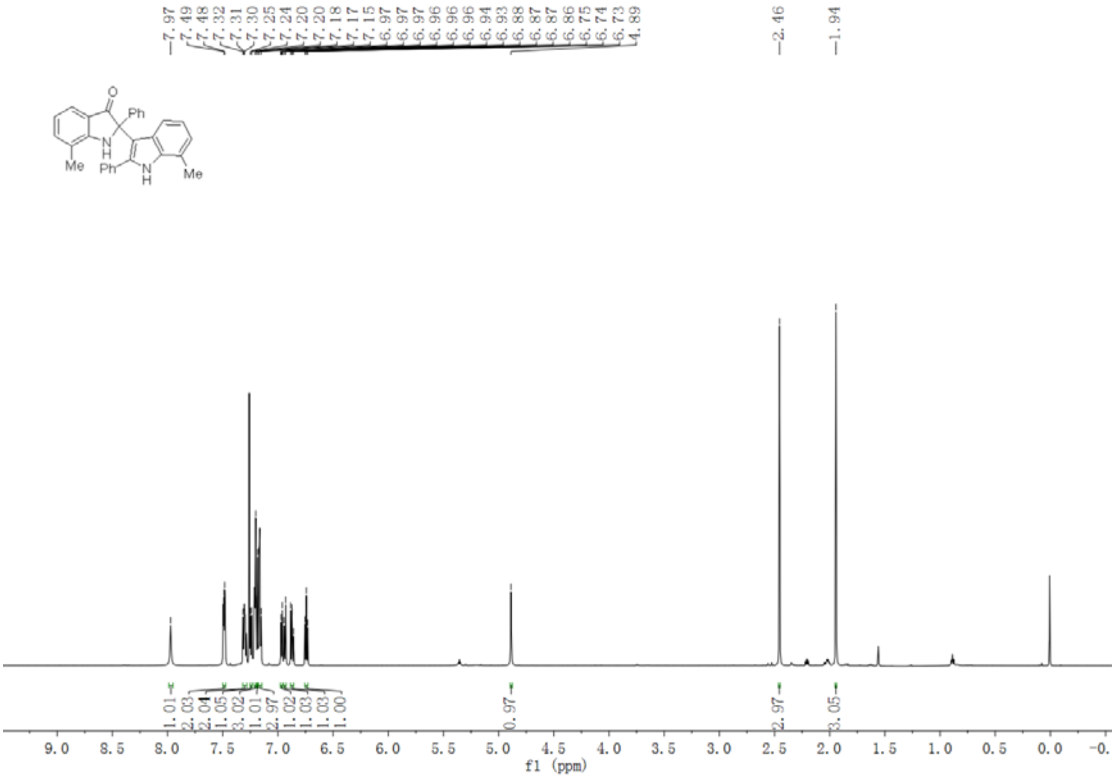

^{13}C NMR spectrum of compound 6h in $CDCl_3$ (151 MHz)

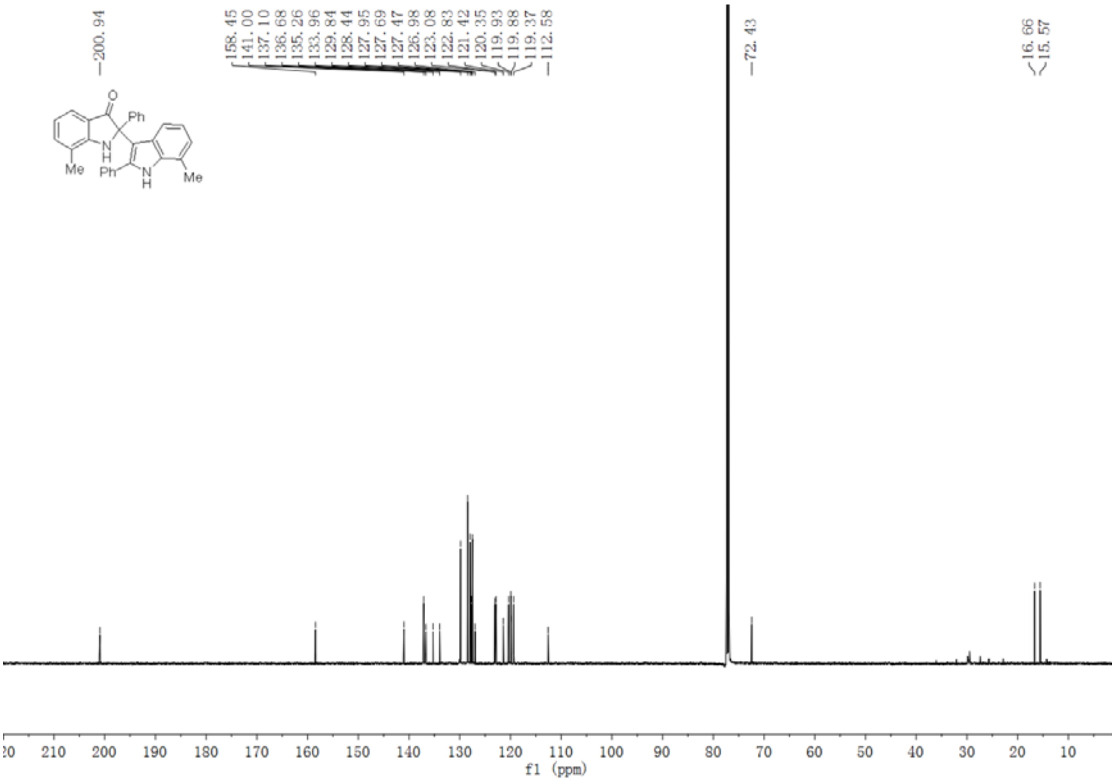

^{1}H NMR spectrum of compound 7a in acetone-d_6 (600 MHz)

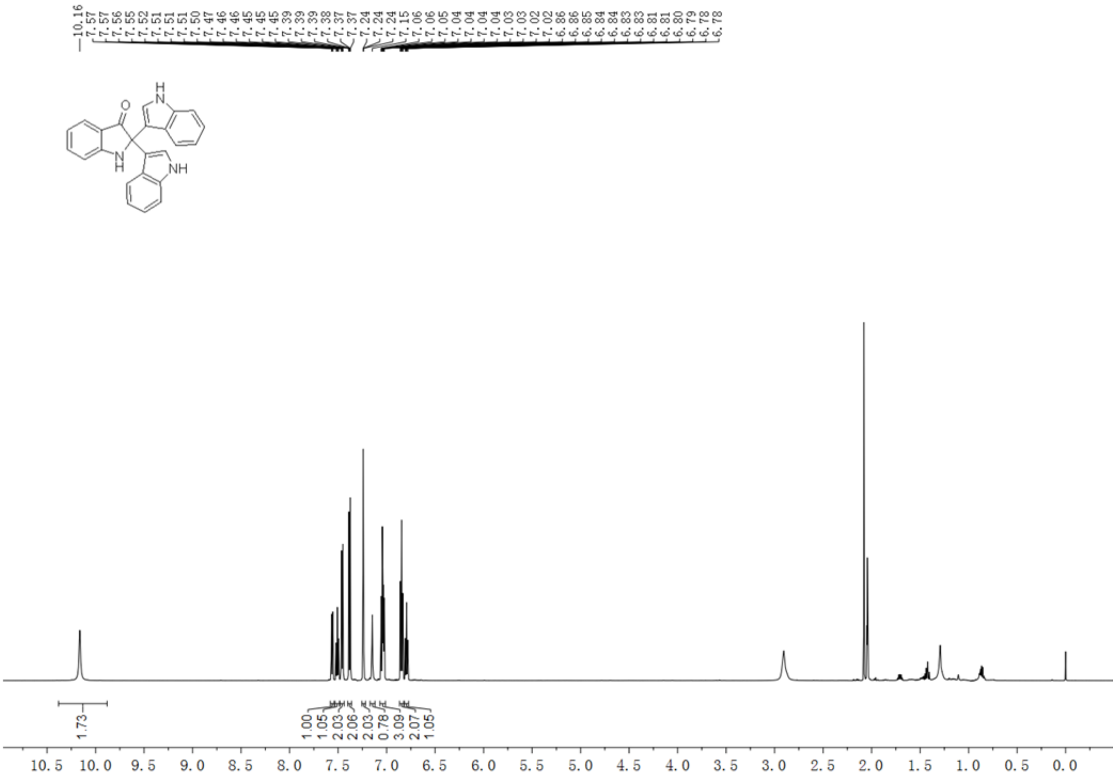

^{13}C NMR spectrum of compound 7a in acetone-d_6 (151 MHz)

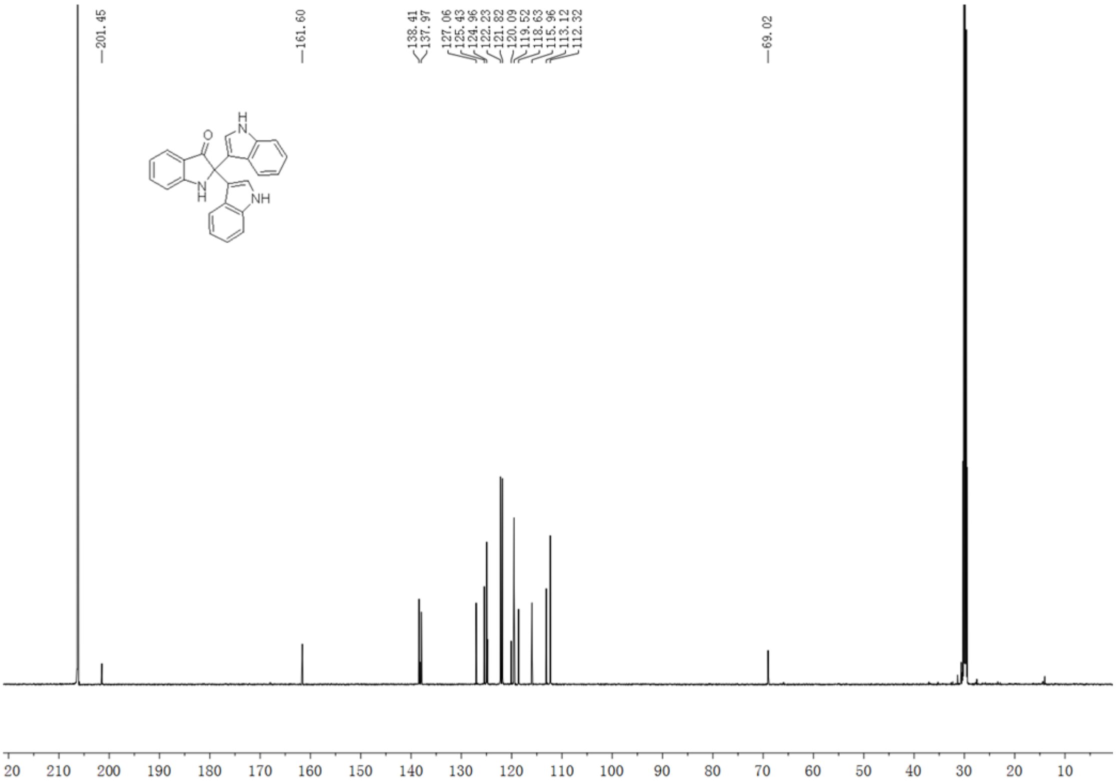

^{1}H NMR spectrum of compound 7b in DMSO-d_6 (600 MHz)

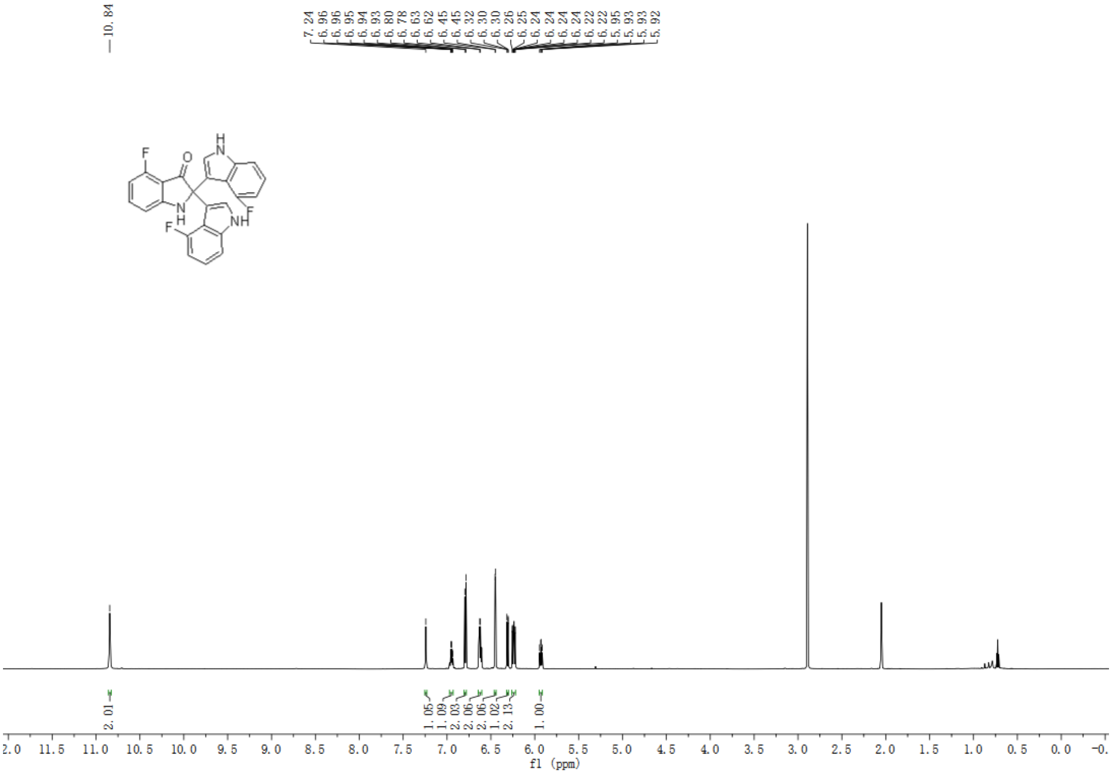

^{13}C NMR spectrum of compound 7b in DMSO-d_6 (151 MHz)

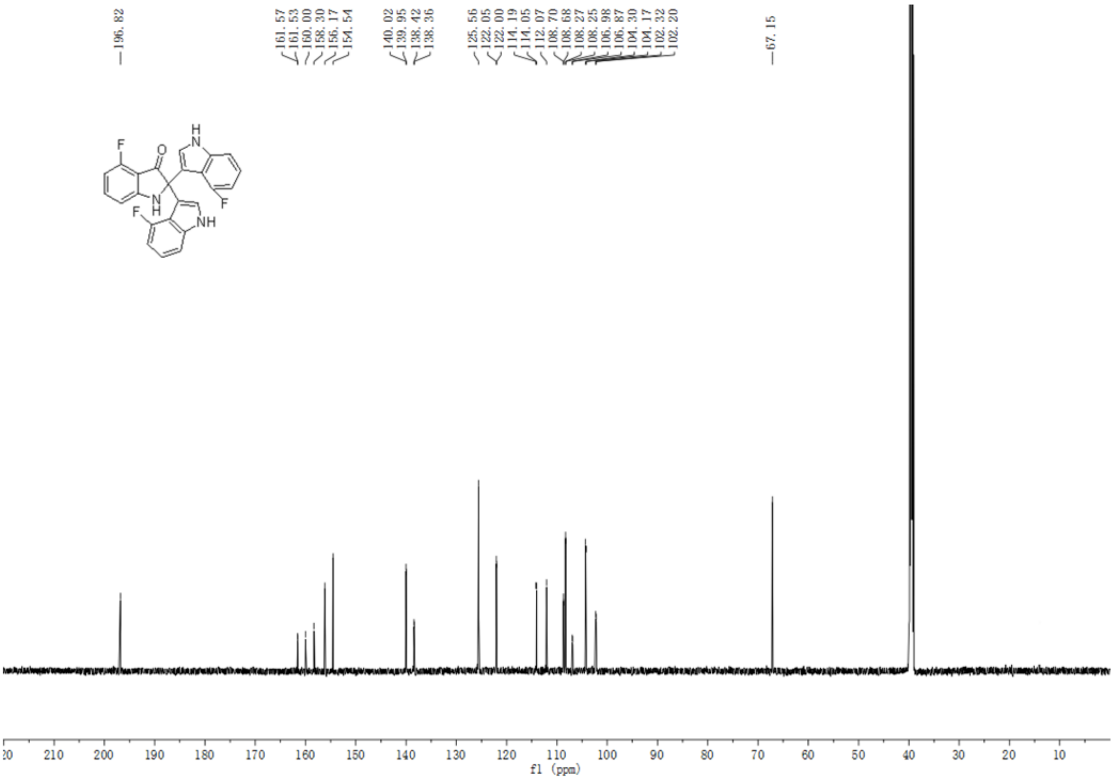

^{1}H NMR spectrum of compound 7c in $CDCl_3$ (600 MHz)

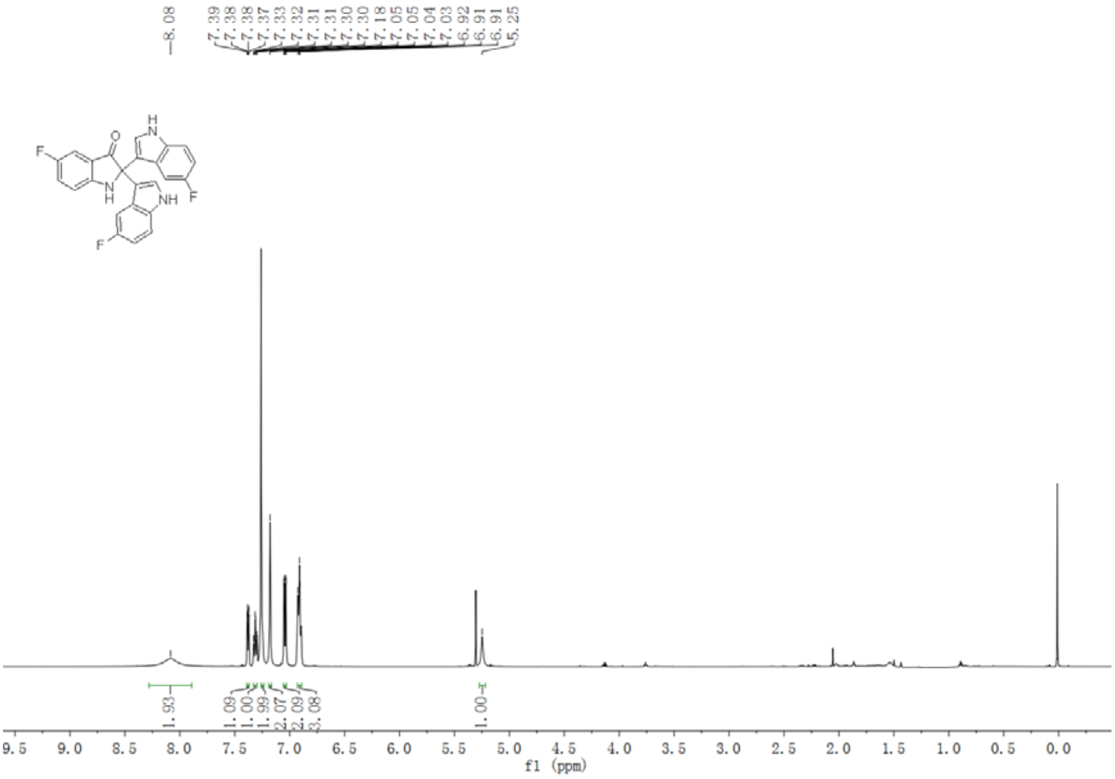

^{13}C NMR spectrum of compound 7c in $CDCl_3$ (151 MHz)

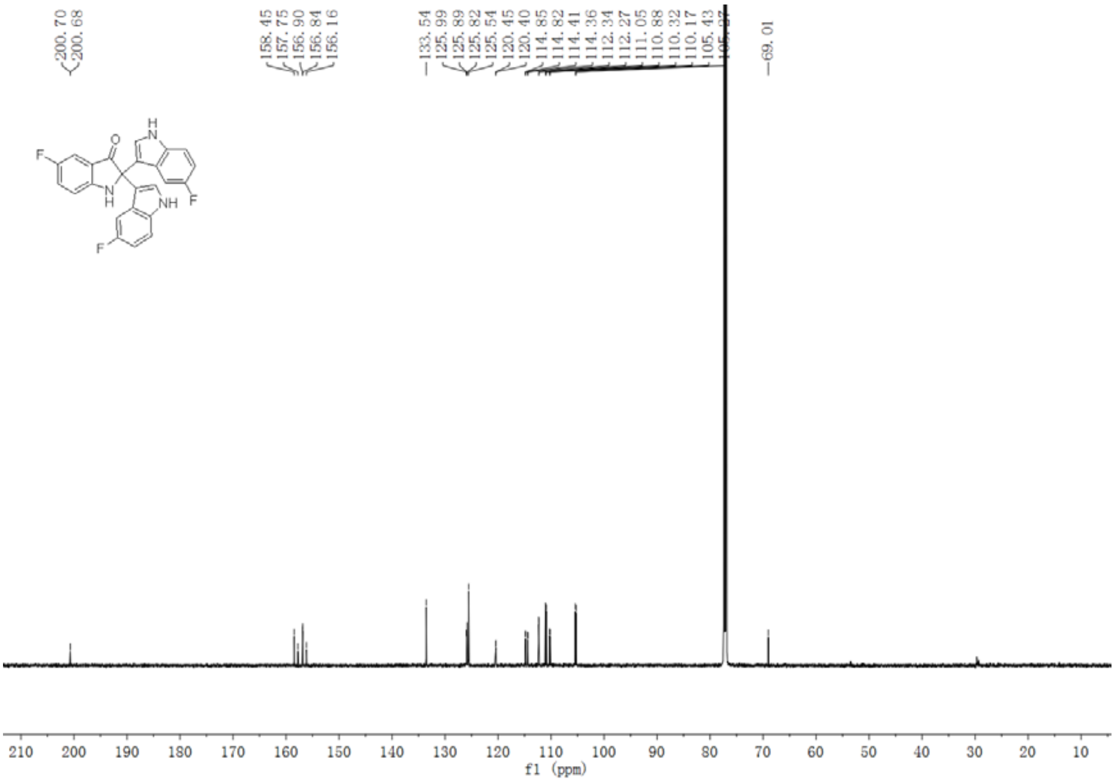

^{1}H NMR spectrum of compound 7d in acetone-d_6 (600 MHz)

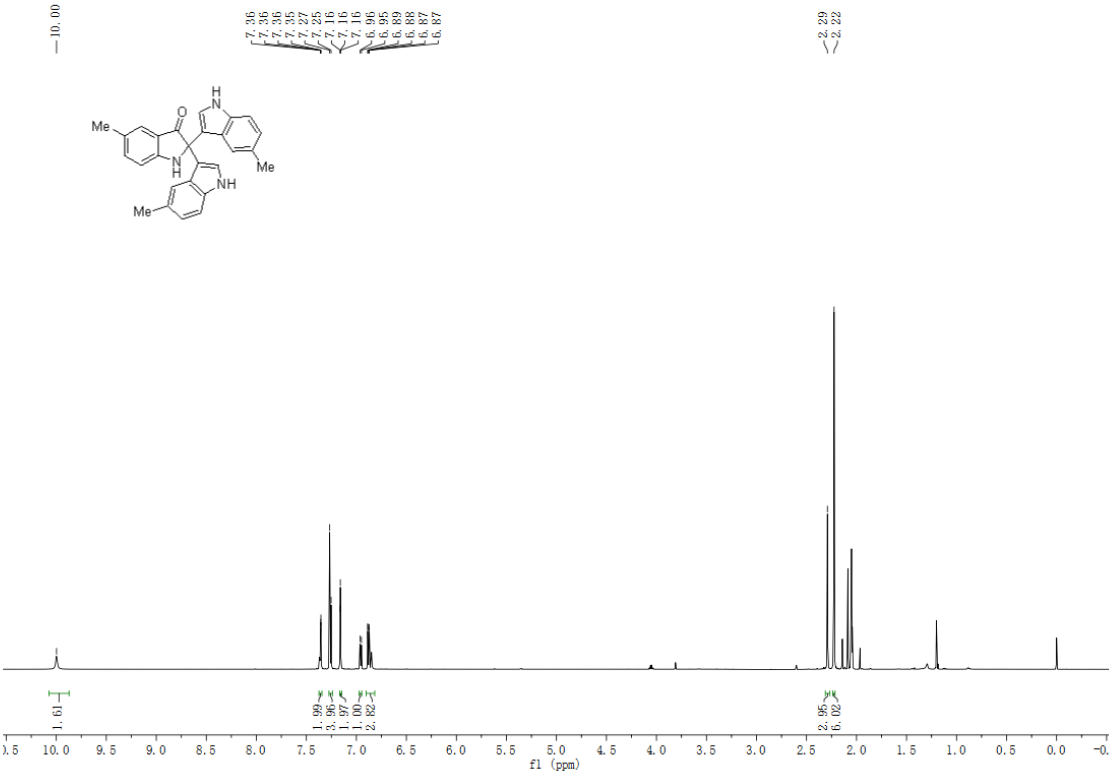

^{13}C NMR spectrum of compound 7d in acetone-d_6 (151 MHz)

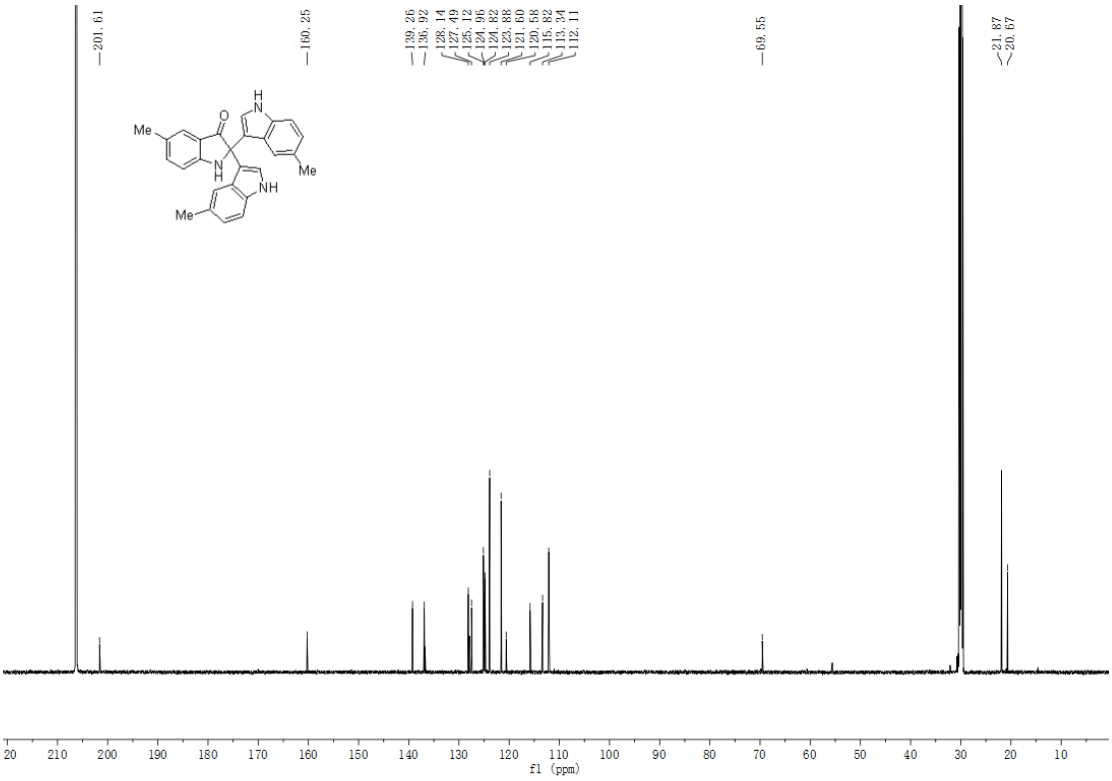

^{1}H NMR spectrum of compound 7e in DMSO-d_6 (600 MHz)

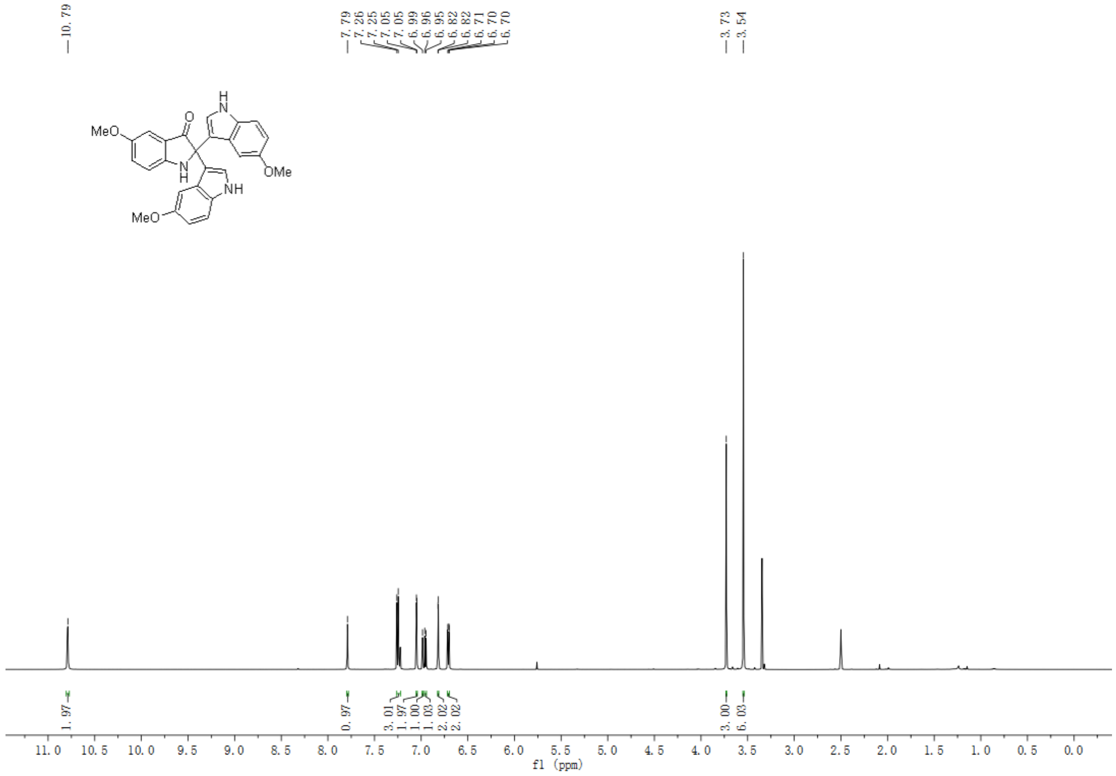

^{13}C NMR spectrum of compound 7e in DMSO-d_6 (151 MHz)

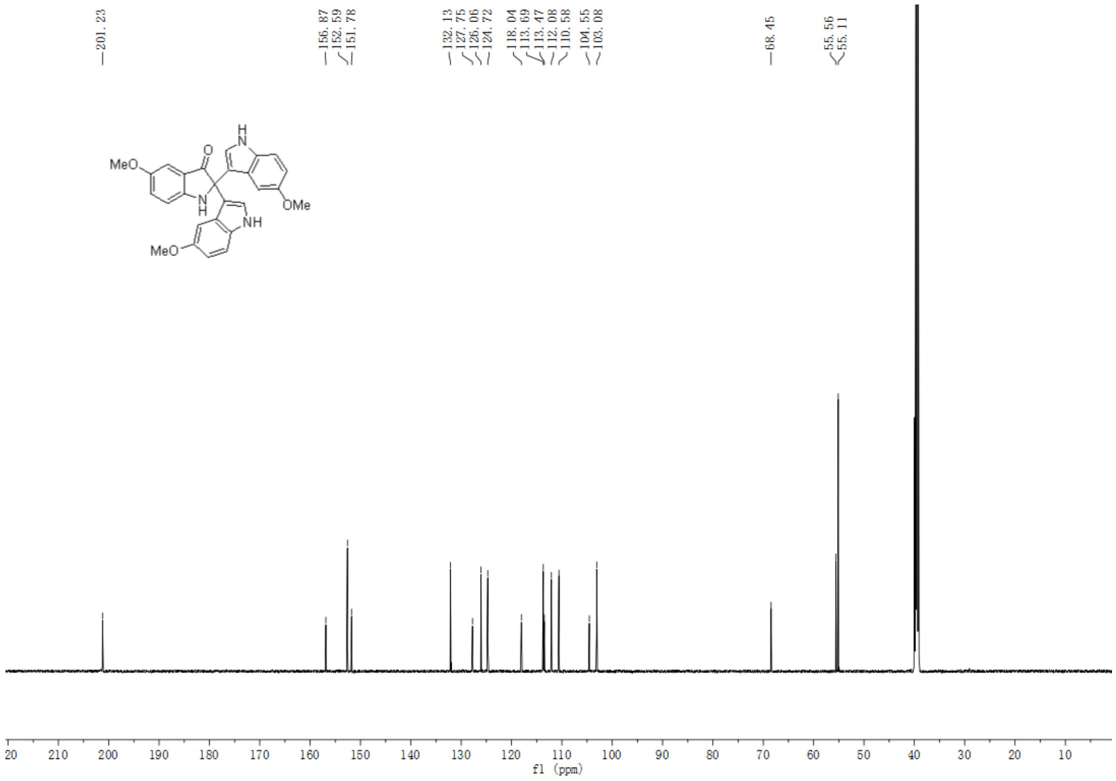

^{1}H NMR spectrum of compound 7f in acetone-d_6 (600 MHz)

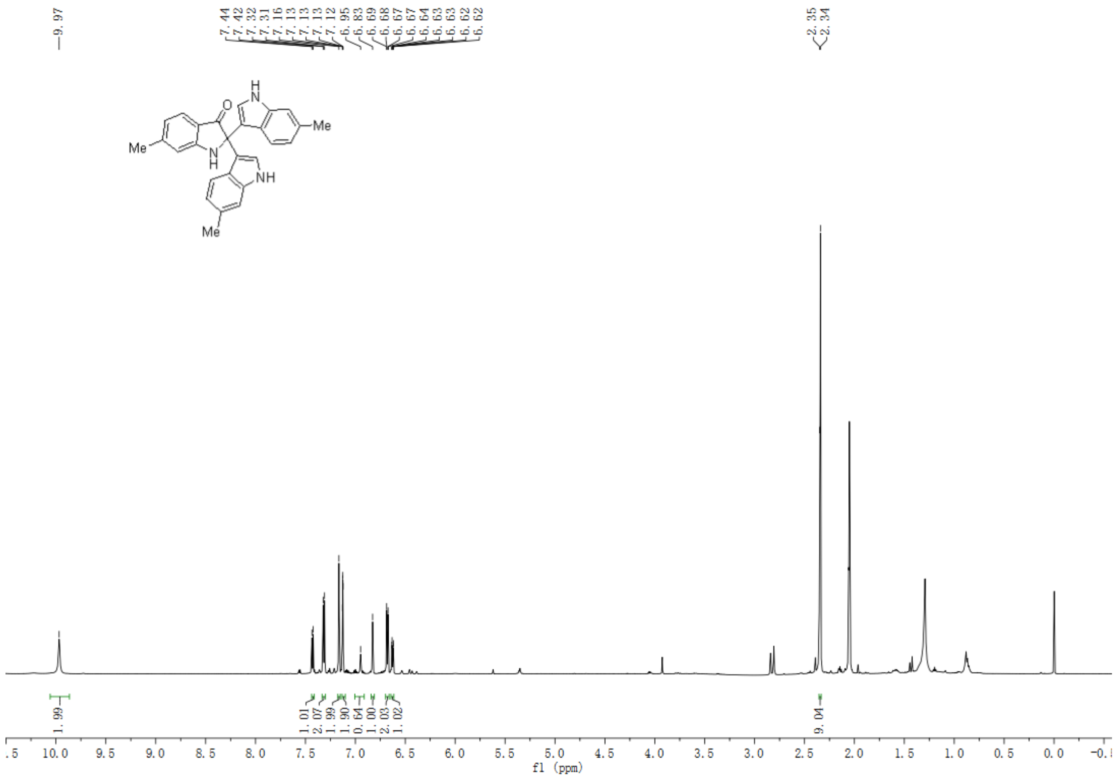

^{13}C NMR spectrum of compound 7f in acetone-d_6 (151 MHz)

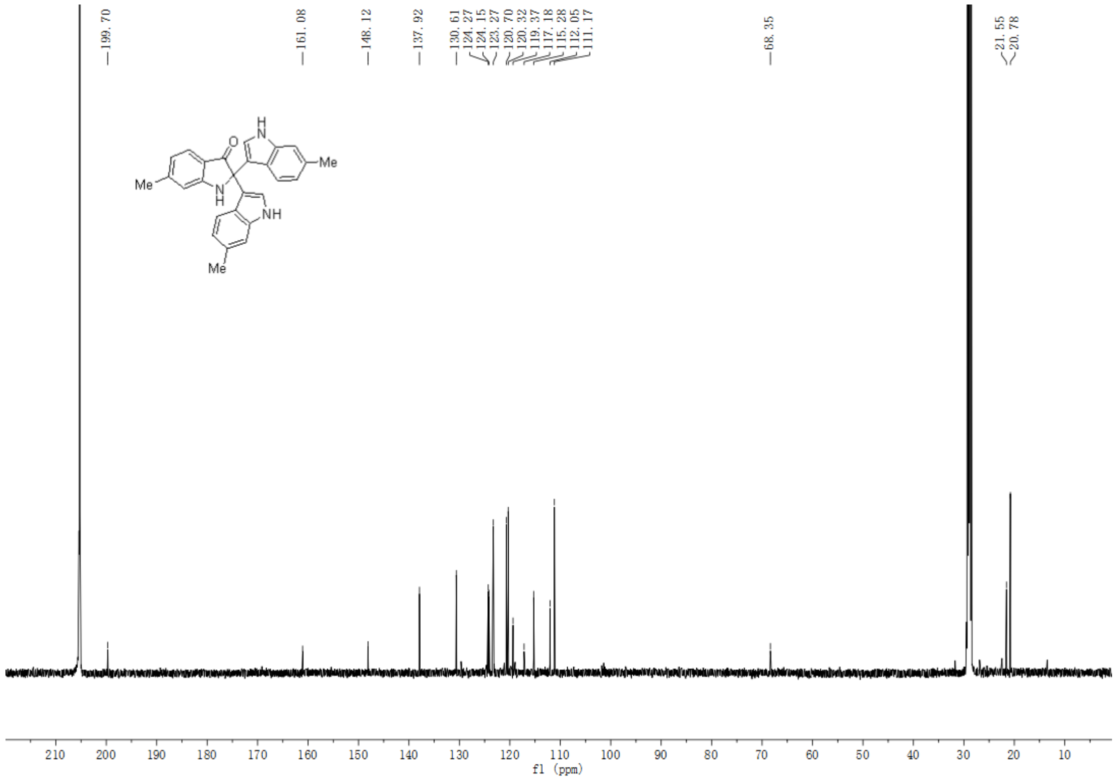

^{1}H NMR spectrum of compound 7g in acetone-d_6 (600 MHz)

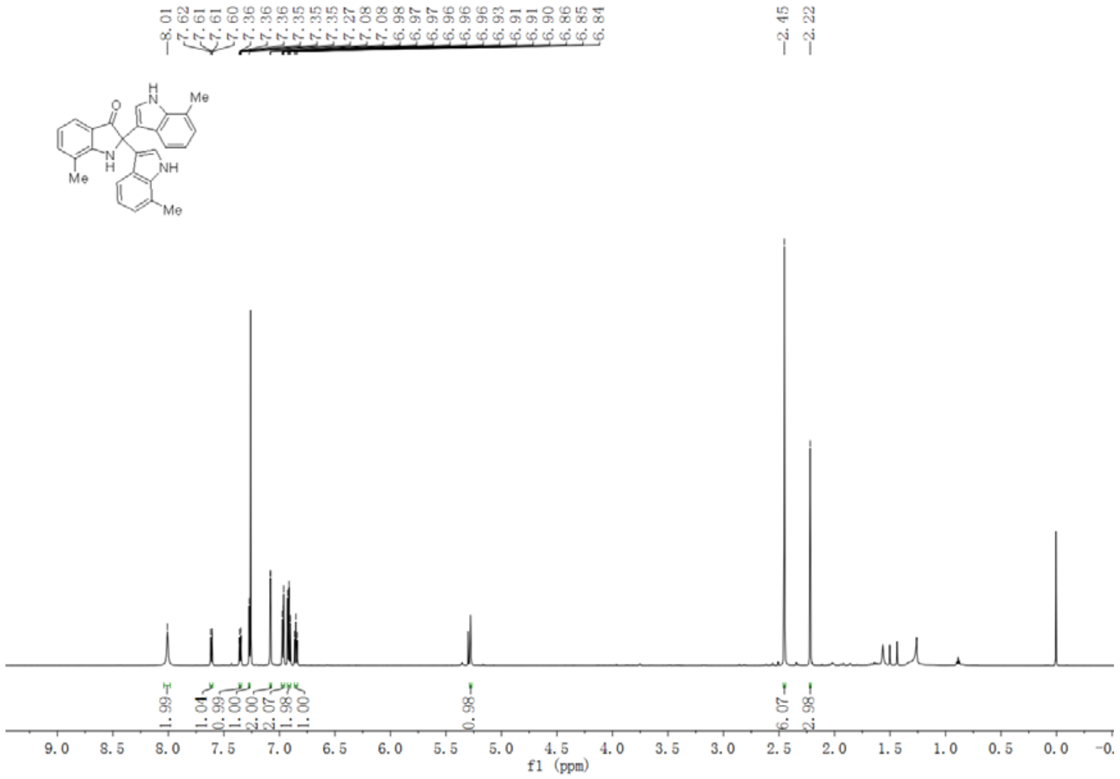

^{13}C NMR spectrum of compound 7g in acetone-d_6 (151 MHz)

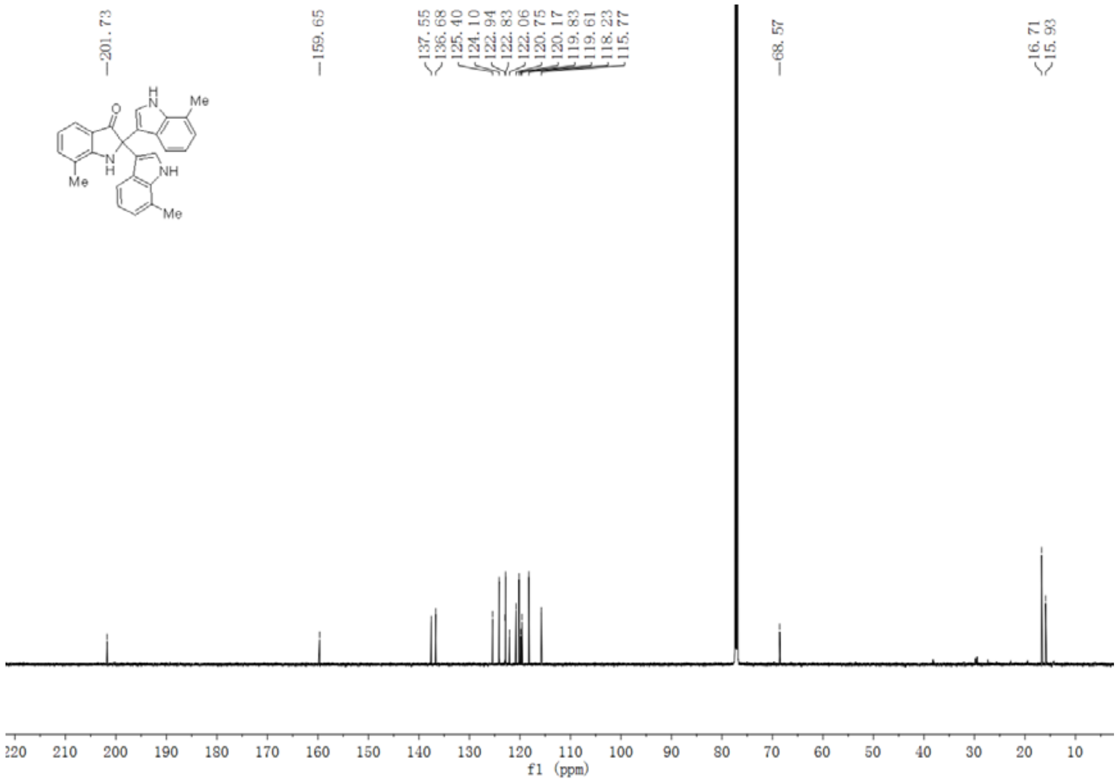

^{1}H NMR spectrum of compound 8 in $CDCl_3$ (600 MHz)

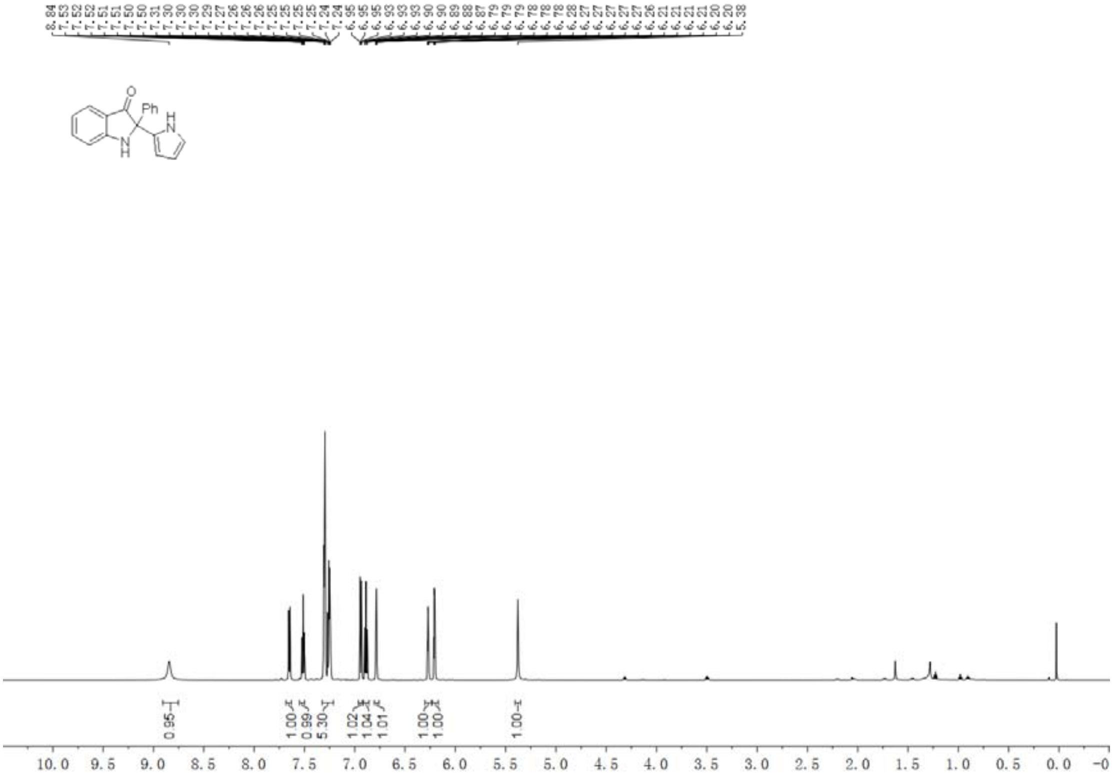

^{13}C NMR spectrum of compound 8 in $CDCl_3$ (151 MHz)

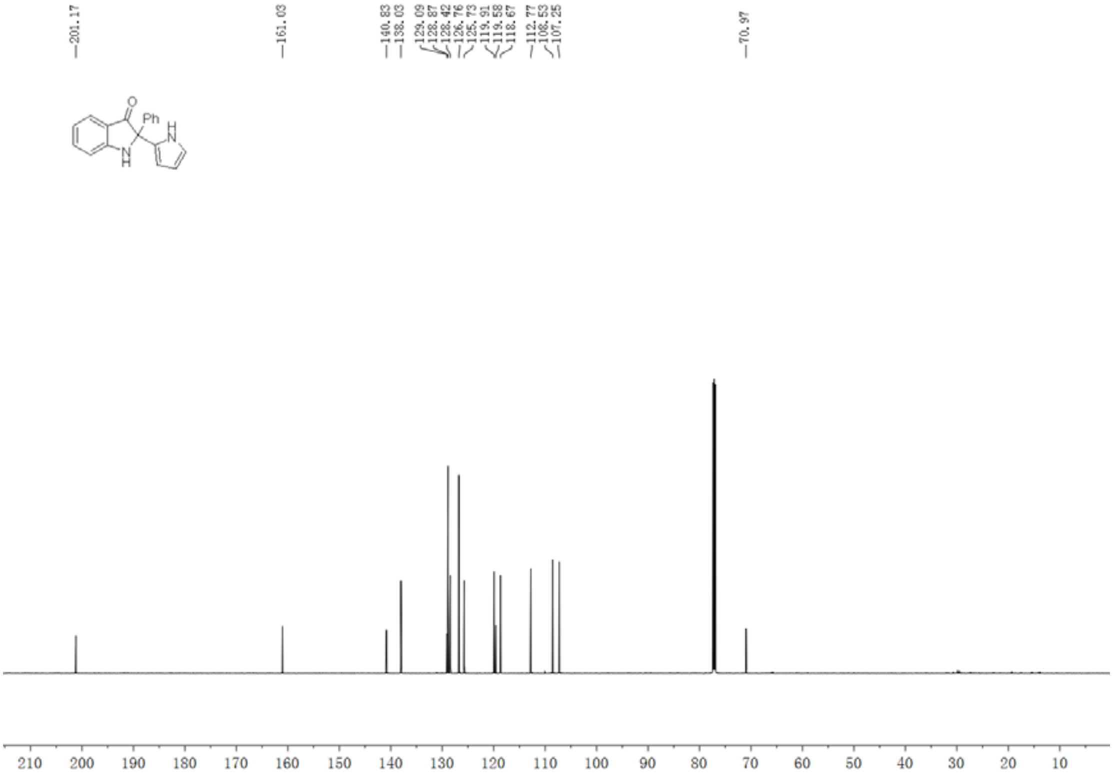

^{1}H NMR spectrum of compound 9 in $CDCl_3$ (600 MHz)

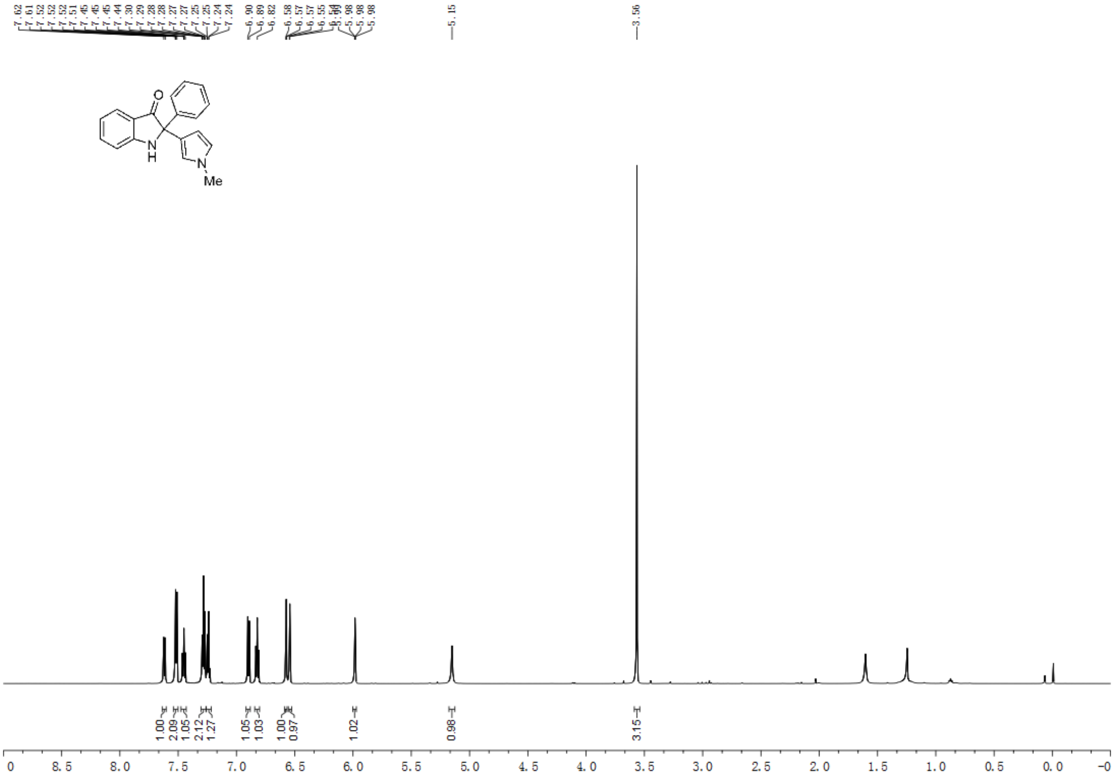

^{13}C NMR spectrum of compound 9 in $CDCl_3$ (151 MHz)

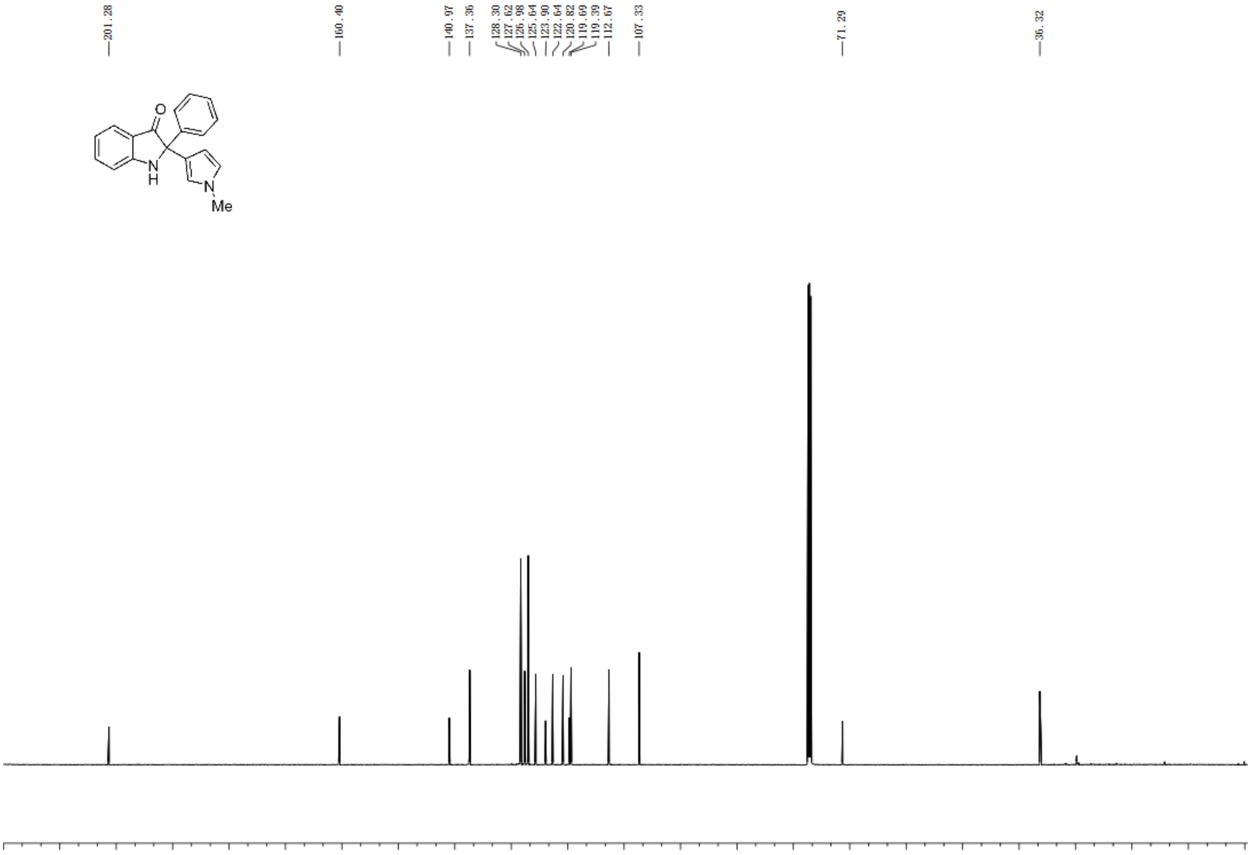

^{1}H NMR spectrum of compound 10 in $CDCl_3$ (600 MHz)

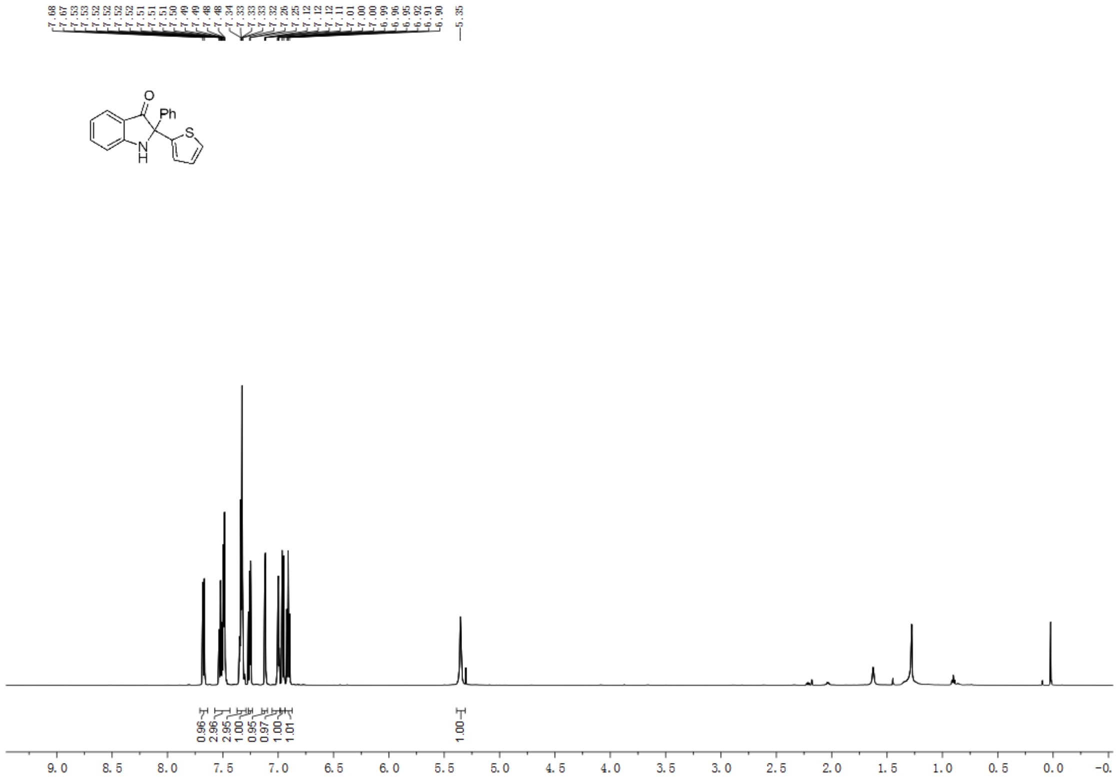

^{13}C NMR spectrum of compound 10 in $CDCl_3$ (151 MHz)

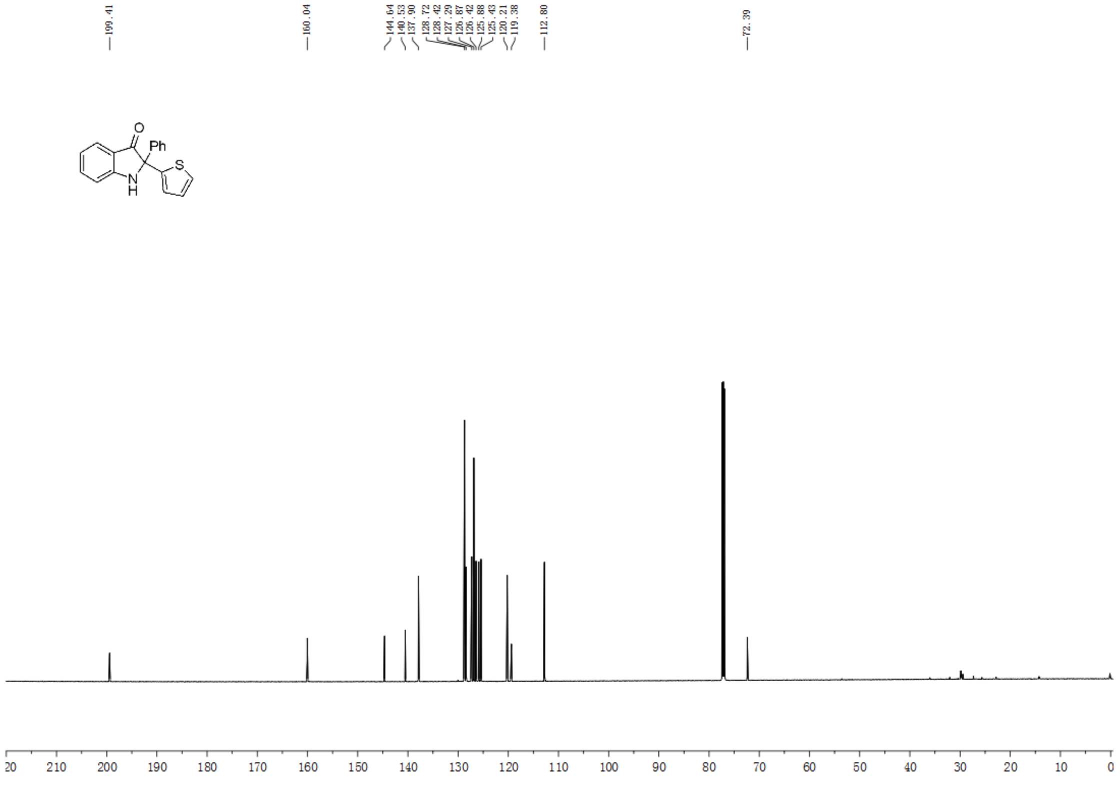

^{1}H NMR spectrum of compound 11 in $CDCl_3$ (600 MHz)

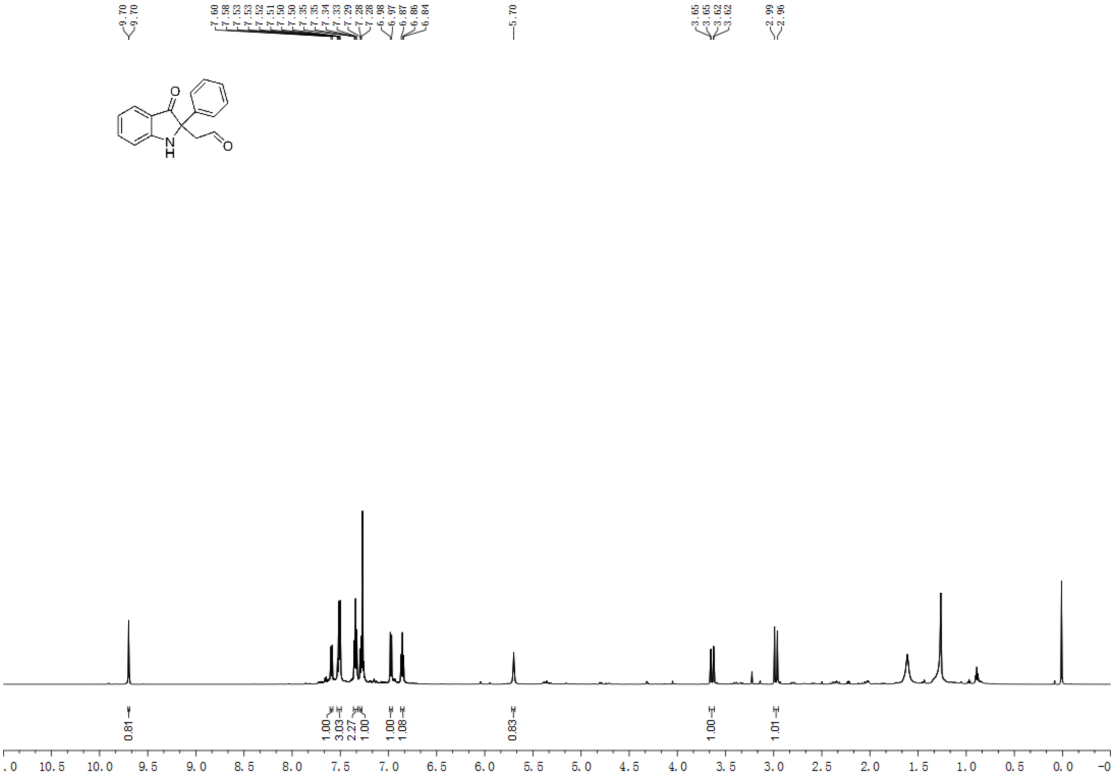

^{13}C NMR spectrum of compound 11 in $CDCl_3$ (151 MHz)

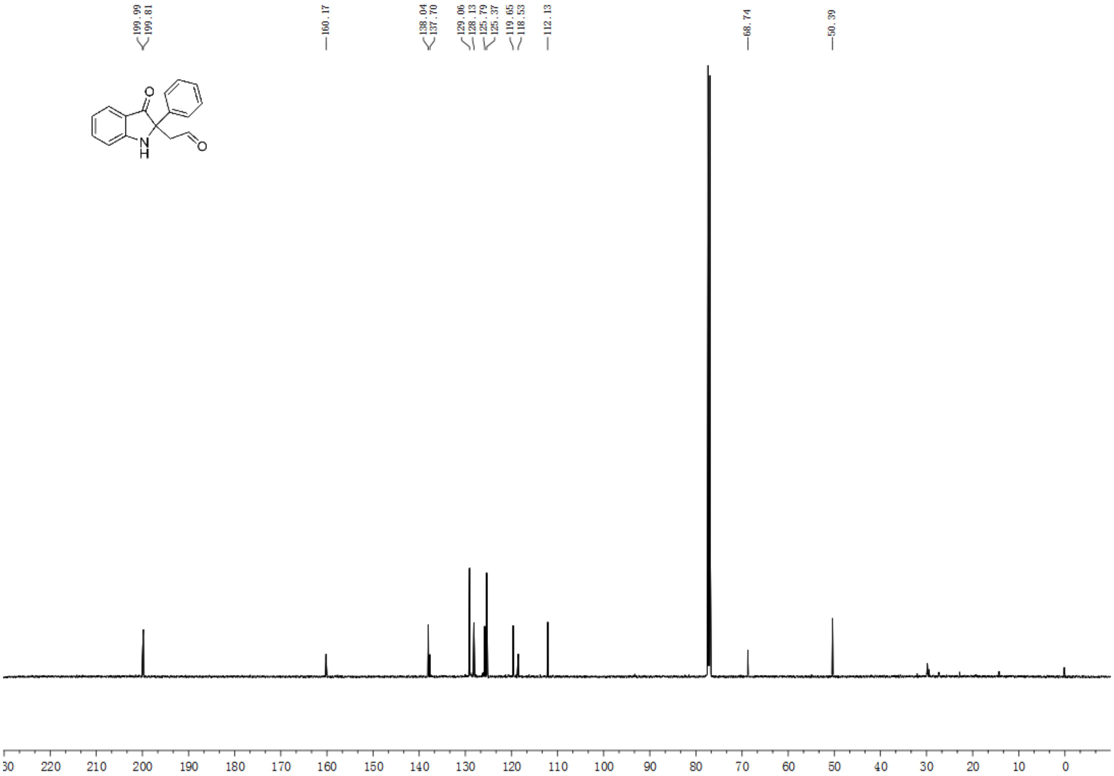

^{1}H NMR spectrum of compound 12 in $CDCl_3$ (600 MHz)

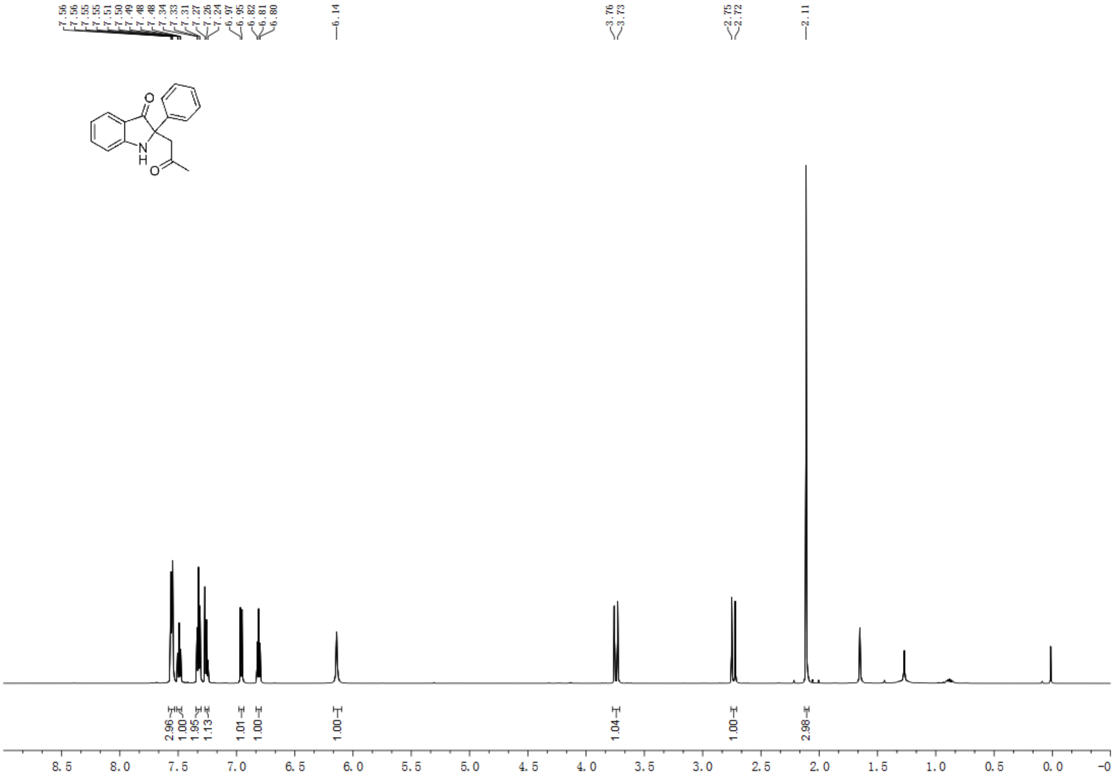

^{13}C NMR spectrum of compound 12 in $CDCl_3$ (151 MHz)

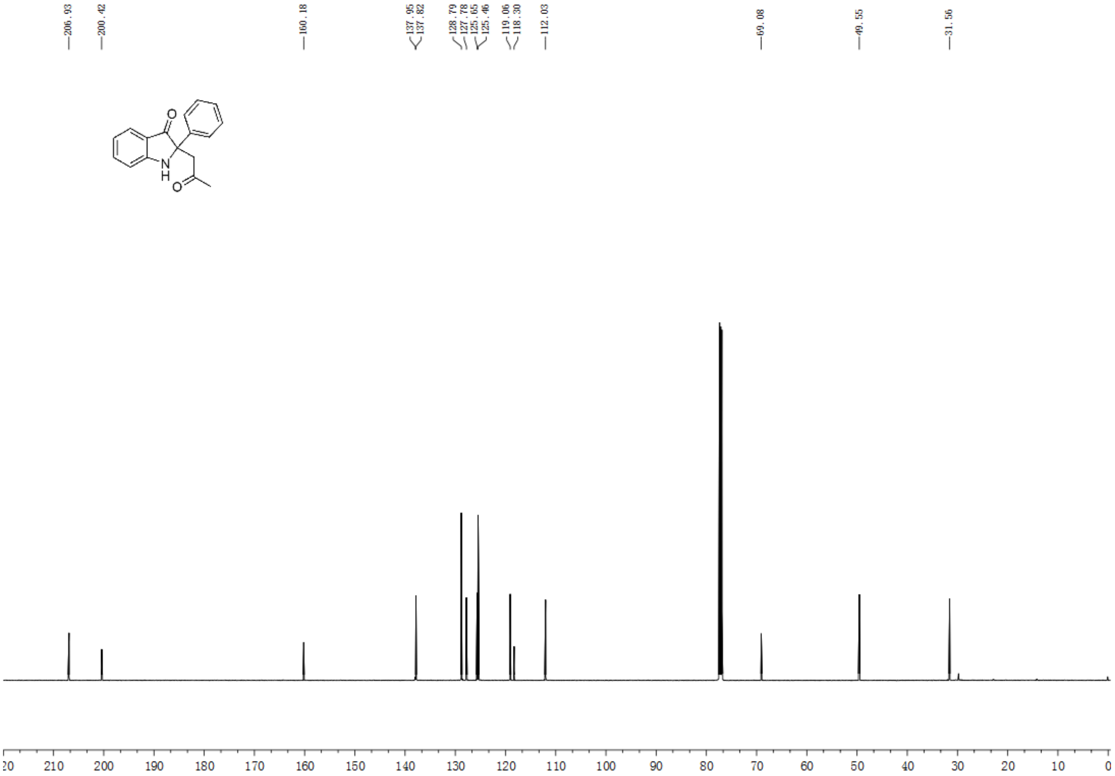

Supplement: Supplementary file 1 [file molecules-25-00419-s001.pdf]
